# Increased Interstitial Concentrations of Glutamate and Pyruvate in Vastus Lateralis of Women with Fibromyalgia Syndrome Are Normalized after an Exercise Intervention – A Case-Control Study

**DOI:** 10.1371/journal.pone.0162010

**Published:** 2016-10-03

**Authors:** Björn Gerdle, Malin Ernberg, Kaisa Mannerkorpi, Britt Larsson, Eva Kosek, Nikolaos Christidis, Bijar Ghafouri

**Affiliations:** 1 Pain and Rehabilitation Centre, and Department of Medical and Health Sciences, Linköping University, Linköping, Sweden; 2 Karolinska Institute, Department of Dental Medicine, Section of Orofacial Pain and Jaw Function and Scandinavian Centre for Orofacial Neuroscience (SCON), Stockholm, Sweden; 3 Section of Physiotherapy, Institute of Neuroscience and Physiology, Sahlgrenska Academy, University of Gothenburg, Gothenburg, Sweden; 4 University of Gothenburg Centre for Person-Centred Care (GPCC), Sahlgrenska Academy, Gothenburg, Sweden; 5 Department of Clinical Neuroscience and Osher Centre for Integrative Medicine, Karolinska Institute, Stockholm, Sweden; Universidad de Sevilla, SPAIN

## Abstract

**Background:**

Fibromyalgia syndrome (FMS) is associated with central alterations, but controversies exist regarding the presence and role of peripheral factors. Microdialysis (MD) can be used *in vivo* to study muscle alterations in FMS. Furthermore for chronic pain conditions such as FMS, the mechanisms for the positive effects of exercise are unclear. This study investigates the interstitial concentrations of algesics and metabolites in the vastus lateralis muscle of 29 women with FMS and 28 healthy women before and after an exercise intervention.

**Methods:**

All the participants went through a clinical examination and completed a questionnaire. In addition, their pressure pain thresholds (PPTs) in their upper and lower extremities were determined. For both groups, MD was conducted in the vastus lateralis muscle before and after a 15-week exercise intervention of mainly resistance training of the lower limbs. Muscle blood flow and interstitial muscle concentrations of lactate, pyruvate, glutamate, glucose, and glycerol were determined.

**Results:**

FMS was associated with significantly increased interstitial concentrations of glutamate, pyruvate, and lactate. After the exercise intervention, the FMS group exhibited significant decreases in pain intensity and in mean interstitial concentrations of glutamate, pyruvate, and glucose. The decrease in pain intensity in FMS correlated significantly with the decreases in pyruvate and glucose. In addition, the FMS group increased their strength and endurance.

**Conclusion:**

This study supports the suggestion that peripheral metabolic and algesic muscle alterations are present in FMS patients and that these alterations contribute to pain. After an exercise intervention, alterations normalized, pain intensity decreased (but not abolished), and strength and endurance improved, all findings that suggest the effects of exercise are partially peripheral.

## Introduction

Fibromyalgia syndrome (FMS) is a common chronic pain condition associated with negative implications [1,2]. FMS is associated with central alterations (e.g., central hyperexcitability with disinhibition [3–7]), but controversies exist regarding the presence and role of peripheral factors. For example, several findings suggest peripheral nociceptive mechanisms such as muscle and nociceptive C-fibre alterations e.g. small fibre neuropathy [8–12] and signs of peripheral nociceptive input [13–17] are at play.

Exercise is beneficial for FMS and chronic widespread pain patients (CWP) [18–21], but the mechanisms for the positive effects of exercise are unclear [22–25].

Microdialysis (MD) is an *in vivo* technique that can be used to sample biochemical substances in the muscle interstitium (i.e., extra cellular fluid), where nociceptor free nerve endings terminate, providing information on the local biochemical milieu. Several MD studies have found increased concentrations of glutamate, serotonin, lactate, and/or pyruvate in the trapezius muscle in regional chronic neck pain conditions [26,27]. MD has also been used to study muscle alterations in FMS and one study found an increased concentration of serotonin in the myalgic masseter muscle of FMS [28]. Two MD studies investigated alterations of the trapezius muscle in FMS: one study found increased interstitial concentrations of lactate and pyruvate–metabolites and products of glycolysis–in the resting trapezius muscle [29] and one study found significantly increased interstitial concentrations of glutamate and lactate from a cohort of mainly FMS patients (i.e., 15 out of the 17 CWP patients)[30]. The latter study also reported that the concentrations of glutamate and lactate correlated with pressure pain thresholds (PPTs) of trapezius and pain intensity in the FMS/CWP group [30]. For the same FMS/CWP cohort was found that endogenous pain inhibitory substances (N-Acylethanolamines; NAE) were mobilized differently than in controls and in chronic regional neck-shoulder pain patients [31]. A relatively small study found no increase in lactate of the vastus lateralis [32]. To develop effective mechanism-based treatments for FMS will require a better understanding of peripheral muscle alterations and how exercise normalizes alterations and influences aspects of pain.

This study investigates the interstitial concentrations of certain metabolites and algesics in the vastus lateralis of women with FMS and in female healthy controls (CON) before and after a 15-week exercise intervention. In addition, this study investigates relationships between concentrations of the biochemical substances and aspects of pain (group and intensity).

## Subjects and Methods

This is a non-randomized sub-study of a multi-centre experimental study comprising women with FMS and healthy women (ClinicalTrials.gov identification number: NCT01226784). The three participating centers were the Pain- and Rehabilitation Medicine, Department of Medical and Health Sciences (IMH), Faculty of Health Sciences, Linköping University, Pain- and Rehabilitation Centre, County Council of Östergötland, Linköping; the Department of Rheumatology and Inflammation Research, Institute of Medicine, Sahlgrenska Academy, University of Gothenburg, Göteborg; and the Department of Clinical Sciences, Karolinska Institutet at Danderyd University Hospital, Stockholm, all in Sweden.

### Subjects

Recruitment procedures have been described in detail in previous articles [33,34]. The following inclusion criteria for women with FMS were used: between 20 and 65 years old and meeting the American College of Rheumatology (ACR) 1990 classification criteria for FMS. Even though there exist newer criteria these are the criteria generally used in clinical practice and in most research studies both in Sweden and in other countries. Moreover, in clinical practise in Sweden these criteria have to be fulfilled in order to obtain the ICD-10 code for FMS (i.e. M79.7). The following exclusion criteria were used: high blood pressure (>160/90 mmHg), osteoarthritis in hip or knee, other severe somatic or psychiatric disorders, primary causes of pain other than FMS, high consumption of alcohol (i.e. Audit >6 according to the recommendations for women), participation in a rehabilitation program within the past year, regular resistance exercise training or relaxation exercise training more than twice a week, inability to understand or speak Swedish, and not being able to refrain from analgesics, NSAID, or hypnotics for 48 hours before examinations.

**Fig 1:**
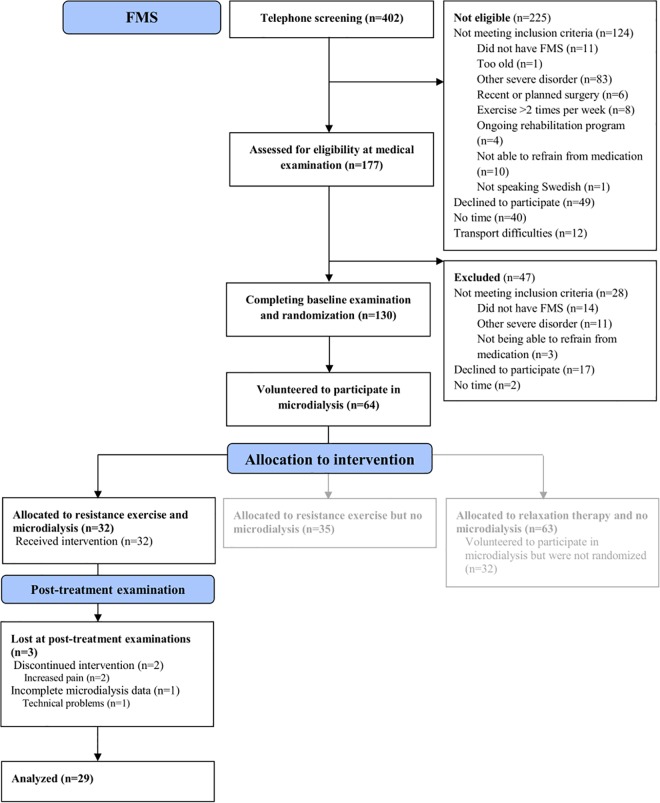
Flow diagram of the recruitment process and participation in the group of patients with fibromyalgia (FMS).

The patients were recruited by newspaper advertisement in the local newspapers of three cities in Sweden (Gothenburg, Stockholm, and Linköping) (**Fig 1**). In response to these advertisements, 402 women expressed an interest to participate in the study. All 402 were telephone screened for possible eligibility. Out of these women, 225 were not eligible for enrolment. The remaining 177 women were then assessed for eligibility during a medical examination. As a result of this examination, 44 additional women were excluded, leaving 133 women with FMS eligible for a multicentre experimental study, which will be reported elsewhere.

**Fig 2:**
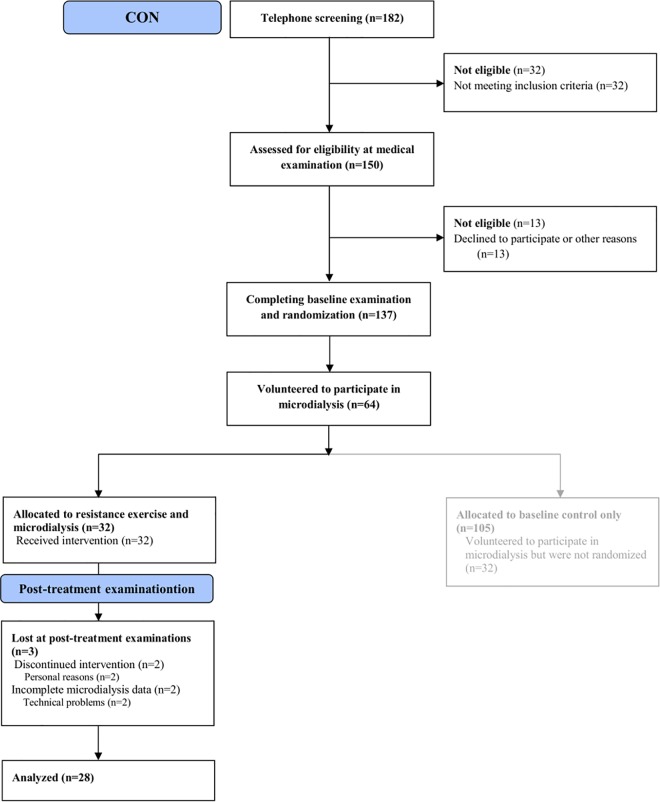
Flow diagram of the recruitment process and participation in the group of healthy controls (CON).

Twenty-eight female controls (CON) participated, approximately coinciding the mean age of the patients with FMS. They were also recruited through advertisements in the local newspapers of the three participating cities (Gothenburg, Stockholm, and Linköping) and formed a subgroup of the 32 controls that participated in the above mentioned trial (**Fig 2**). The inclusion criteria were age between 20 and 65 years, and female sex. Exclusion criteria were any pain condition, high blood pressure (>160/90 mmHg), osteoarthritis (OA) in hip or knee, other severe somatic or psychiatric disorders, high consumption of alcohol (see above), participation in a rehabilitation program within the past year, regular resistance exercise or relaxation exercise twice a week or more, inability to understand or speak Swedish, and not being able to refrain from analgesics, NSAID or hypnotics for 48 hours prior to examination.

This sub-study reports results from microdialysis of the vastus lateralis of the dominant side before and after a progressive resistance exercise training intervention of the lower limbs (15 weeks) for both the FMS group and for the CON group. This sub-study included data from subjects that took part in both MD sessions i.e. 29 patients with FMS and 28 healthy subjects (CON). Research subjects were recruited and followed up from October 2010 to September 2013.

When estimating the sample size for analyzing differences in the interstitial concentration of glutamate (μmol l^-1^) between the groups, we assumed based on a recent study [30] that the mean difference should have a standard deviation of 30 μmol l^-1^. Expectation of a mean difference of 25 μmol l^-1^, required a sample size of 24 subjects in each group to reject the null hypothesis with a power of 0.80 and a probability of <0.05 (two tailed).

The estimation of sample size for analyzing changes within groups the interstitial concentration of glutamate (μmol l^-1^) was based on a recent study [35] and we assumed that the mean difference should have a standard deviation of 25 μmol l^-1^. Expectation of a mean change of 15 μmol l^-1^, required a sample size of 24 pairs of subjects to reject the null hypothesis with a power of 0.80 and a probability of <0.05 (two tailed).

The Sample size calculations were made using the computer program Power and Sample Size Calculations (v. 3.0.43, http://biostat.mc.vanderbilt.edu/wiki/Main/PowerSampleSize).

Due to the fact that this study was an intervention study with risk for drop-outs approximately 30 subjects in each group was considered as sufficient.

### Methods

After receiving verbal and written information about the study, all subjects signed a consent form in accordance with the Declaration of Helsinki. The study, including the economical compensation, was granted ethical clearances by the Regional ethics committee in Stockholm (Dnr: 2010/1121-31/3).

#### Procedures

CON and FMS participated in a 15-week exercise intervention program that mainly trained the lower limbs. Before the exercise intervention anthropometric data, blood pressures, psychological distress and aspects of quality of life were collected. At this time, the FMS participants were also examined for the number of tender points and pain duration. Before and after the exercise intervention, pressure pain threshold (PPT) and physical capacity (i.e., functional tests for strength of upper and lower limbs and six-minute walking distance) data were collected. In addition, MD was performed of the vastus lateralis muscle. During both these MD sessions, which included a 20 minutes standardised work period, the following data were collected five separate times every 20 minutes: pain intensity, blood flow, and the interstitial muscle concentrations of glucose, lactate, pyruvate, glycerol, and glutamate from vastus lateralis.

#### Background and Anthropometric data

The age (years), weight (kg), height (cm), Body Mass Index (BMI; kg/m^2^), and systolic and diastolic blood pressures (mm Hg) were collected for all the subjects. The participants completed a brief questionnaire regarding depressive and anxiety symptoms as well as quality of life (**Table 1**). At the clinical examination, the number of tender points (ACR criteria) and pain duration was determined for the subjects of the FMS group.

**Table 1:** Age, anthropometric data, depressive and anxiety symptoms, quality of life and pain duration (only FMS), blood pressure and pressure pain threshold and number of tender points (only FMS) in patients with fibromyalgia syndrome (FMS) and in healthy controls (CON).

*Group*	*FMS*	*N = 29*	*CON*	*N = 28*	*Statistical comparison*
*Variables*	*Mean*	*SD*	*Mean*	*SD*	*p-value*
Age (years)	53.7	8.9	54.8	8.1	.737
Height (m)	1.64	0.07	1.65	0.06	.994
Weight (kg)	73.3	13.4	66.8	10.9	.043*
BMI (kg/m^2^)	27.2	5.2	24.7	4.6	.029*
BP diastolic (mm Hg)	83	8	83	7	.885
BP systolic (mm Hg)	133	19	131	14	.797
PPT-all sites (kPa)	184	73	336	100	< .001*
Nos. tender points	15.9	1.4	NA	NA	NA
Pain duration (years)	13.3	7.9	NA	NA	NA
HAD-Depression	6.5	4.1	1.5	1.8	< .001*
HAD-Anxiety	7.0	4.4	3.3	3.1	.001*
SF36-PSC	31.0	7.6	54.6	4.4	< .001*
SF36-MSC	42.0	11.8	51.4	6.0	.003*

Mean ± one standard deviation (SD) is given. Furthest to the right is the result of the statistical comparison between the two groups (p-value)

* denotes significant group difference.

NA denotes not applicable.

BMI = Body Mass Index; BP = blood pressure; HADS-Anxiety = Hospital Anxiety and Depression Scale—subscale anxiety; HADS-Depression = Hospital Anxiety and Depression Scale—subscale depression; PPT-all sites = pressure pain thresholds–mean for all sites; SF36-PCS = Short Form Health Survey 36-physical summary component; SF36-MSC = Short Form Health Survey 36-mental (psychological) summary component.

*The Hospital Anxiety and Depression Scale* (HADS), a short self-assessment questionnaire that measures anxiety and depression, comprises seven items in each of the depression (HAD-D) and anxiety (HAD-A) scales [36,37]. Possible subscale scores range from 0 to 21, with the lower score indicating the least depression and anxiety possible. A score of 7 or less indicates a non-case, a score of 8–10 indicates a doubtful case, and a score of 11 or more indicates a definite case.

*The Short Form Health Survey* (SF36) measures multi-dimensional health concepts and measurements of a full range of health states, including levels of well-being and personal evaluations of health. The instrument has eight dimensions and uses a scale from 0 to 100: physical functioning, role limitations due to physical functioning, bodily pain, general health, vitality, social functioning, role limitations due to emotional problems, and mental health [38]. Based on these eight scales, the instrument calculates a physical summary component (SF36-PSC) and a mental (psychological) summary component (SF36-MSC). This study uses the two summary components.

#### Functional tests of physical capacity

Maximal isometric elbow flexion force (kg) in both arms was measured using a dynamometer (Isobex®; Medical Device Solutions AG, Oberburg, Switzerland). The participant was in a seated position without back support and legs stretched out. The upper arm was aligned with the trunk and the elbow in 90° of flexion. The maximum strength obtained during five seconds was recorded [39].

Static knee extension strength (N) was determined using a dynamometer (Steve Strong®; Stig Starke HBI, Gothenburg, Sweden). The participant was in a fixed seated position with back support, knee and hip in 90° of flexion, and legs hanging freely. A non-elastic strap was placed around the ankle and attached to a pressure transducer with an amplifier. The maximum strength obtained during five seconds was recorded. The instrument has been used in previous studies of physical performance [40,41]. The distance that a person can cover in a six minutes (six-minute walk test; WT-6min) was also recorded [42].

#### Pressure pain thresholds

As a part of the clinical examination, algometry was performed using an electronic pressure algometer (Somedic, Hörby, Sweden) as previously described [43]. The algometry was conducted approximately one week before the microdialysis. The area of the contact area was 1 cm^2^ and pressure was applied perpendicularly to the skin at a speed corresponding to approximately 50 kPa/s. The subjects were instructed to mark the pressure pain threshold (PPT) by pressing a button as the sensation of “pressure” changed to “pain”. When the button was pressed or when the maximum pressure of 1 500 kPa was reached, the application of pressure ceased. Algometry was performed bilaterally over the supraspinatus muscle (at origins above the scapula spine near the medial border), the lateral epicondyle (2-cm distal to the epicondyles), over the gluteus maximus (in upper outer quadrants of buttocks in anterior fold of muscle), and inside of the knee (at the medial fat pad proximal to the joint line). That is, the algometry was used on eight of the eighteen tender points defined by the ACR criteria [44]. In the present study, a mean value of all eight anatomical sites was used. Before the actual testing of PPT, the subjects were given instructions and were familiarized with the testing procedure.

#### The 15-week exercise intervention

The exercise intervention has been described in detail elsewhere [34]; here is given a brief description. The main goal of the exercise intervention was to improve muscle strength and health status by progressive resistance exercise, but without risking increased pain while loading the muscles [34]. The exercise intervention lasted 15 weeks and each session was performed twice a week. Each session was performed under the supervision of specially trained physical therapists. It was conducted at physiotherapy premises and at a local gym at the different sites in groups compromising five to seven participants to promote interaction between participants and to facilitate physiotherapeutic guidance. The intervention was proceeded by an individual introductory meeting. Low intensity of exercise adjusted to individual limitations works well in most patients with FMS [45]. For this study, we initiated resistance training at 40% of the one repetition maximum (1 RM) and successively progressed to 70–80% of 1RM. This exercise mode has previously shown to be feasible in FMS [46]. Each session started with a 10-minute warm up (stationary bicycle) followed by a 50-minute resistance training protocol. In order to promote the participant´s sense of control and to avoid possible negative effects related to exercise, the exercise was initiated at low loads, and possibilities for progression of loads were evaluated every 3–4 weeks in dialogue between the physiotherapist and participant. The physiotherapists were not blinded for group belonging (FMS or CON).

#### Microdialysis and sample preparation

MD mimics the function of a capillary blood vessel by perfusing a thin dialysis tube implanted in the tissue with a physiological saline solution. Substances can pass by simple diffusion across the dialysis membrane along the concentration gradient. The dialysate is analysed chemically. MD allows for continuous sampling of compounds in the muscle interstitial space, where nociceptor-free nerve endings terminate in close proximity to the muscle fibres, providing accurate information on local biochemical changes before such compounds are diluted and cleared by the circulatory system.

The MD session was performed a few days after the clinical examination when the PPTs were determined. The participants were asked not to perform strenuous exercise two days before the study. They were also instructed not to drink any beverages with caffeine and not to smoke on the day of the study. In addition, they were asked not to take paracetamol-medication two days before and NSAID-medication one week before the MD sessions. The participants arrived at the clinical department in the morning after having eaten breakfast. All subjects reported that they had followed the instructions. During the study, they received a standardised meal at the end of the trauma period, but otherwise they were not allowed to eat. They could, however, drink water.

The skin and the subcutaneous tissues above where the catheter entered were anaesthetised with a local injection (0.5 ml) of lidocaine (Xylocain® 20 mg/ml, AstraZeneca, Södertälje, Sweden) without adrenaline. Care was taken not to anaesthetise the underlying muscle. Two commercially available microdialysis catheters–cut-off points of 100 (CMA 71) and 20 kDa (CMA 60), CMA Microdialysis AB, Solna, Sweden; membrane 30-mm length, 0.5-mm diameter–were inserted parallel to the muscle fibres into the vastus lateralis muscle at half the distance between the trochanter and the knee on the dominant side. Typically, a brief involuntary contraction and change of resistance were perceived when the tip of the insertion needle of the catheter entered the fascia and the muscle. To determine the concentrations of small molecules such as lactate, pyruvate, glutamate, and glucose, the catheter with the 20 kDa cut-off was used [47]. The catheters were perfused with a high-precision syringe pump (CMA 107; CMA/Microdialysis AB, Stockholm, Sweden) at a rate of 5 μl/min with a Ringer acetate solution (Fresenius Kabi AB, Uppsala, Sweden) containing 3-mM glucose, 0.5-mM lactate, and 3.0-μM [^14^C]-lactate (specific activity: 5.81 GBq/mmol, GE Healthcare, Buckinghamshire, UK). This procedure was performed according to the internal reference method [48]. Furthermore, nutritive muscle blood flow was estimated by the microdialysis ethanol technique [49] using ^3^H_2_O instead of ethanol [50]. In addition, 0.3-μl/ml ^3^H_2_O (specific activity: 37 MBq/g; PerkinElmer Life Sciences, Boston, USA) was added to the perfusate. The ratio of ^3^H_2_O in the dialysate and the perfusate (the outflow-to-inflow ratio) varies inversely with the local blood flow in the tissue [49,50].

Immediately after the insertion of catheters, participants rested comfortably in a supine position on a bed for 120 minutes (i.e., the *trauma period*) to allow the tissue to recover from possible changes induced in the interstitial environment. The samples of dialysate from the trauma period were not considered in the present study, which focussed upon the habitual situation of the muscle tissue. After the trauma period, participants continued to rest for 20 minutes–the baseline period (denoted *baseline*). The baseline period was followed by a 20-minute period of standardised repetitive dynamic low-intensity exercise of the quadriceps (thigh muscles) (in the following denoted as *20-min work perio*d). The subjects were seated on a bed (back supported) with the knee slightly bent and the lower leg resting on an exercise ball (diameter 55 cm). Participants with current pain intensity in the exercising leg > 40 on a 0–100 visual analogue scale (VAS) started at 15° of knee flexion and those with less pain started at 20° of knee flexion. During the 20-min work period, the participants were asked to slowly extend the knee to a straight position (0° knee angle), slowly lower it on the ball, and then immediately repeat the cycle without resting on the ball. Each repetition took five seconds. The experiment ended with a recovery period of 60 minutes during which participants rested on the bed (denoted *recov#1–3*). During the MD session, subjects rated the overall pain intensity using a 100 mm VAS (endpoints: 0 = no pain and 100 = worst possible pain). Pain intensity was registered every 20 minutes throughout the experiments except for the exercise period (then every five minutes).

Samples from both catheters were obtained every 20 minutes for the 220 minutes of testing, weighed immediately, and kept on ice throughout the rest of the MD experiment. The samples were then stored as aliquots in -70°C until analysis. All vials were weighed before the experiment started and after each 20-minute interval to confirm that sampling and fluid recovery (FR) was working according to the perfusion rate set. Vials with visible sign of haemolysis were discarded.

Relative recovery (RR) measurements: A 5-μL dialysate or perfusate was pipetted into a counting vial containing 3-ml scintillation fluid (High-flash Point, Universal LSC-Cocktail, ULTIMA GOLD™, PerkinElmer, Inc., MA, U.S.A) and vortexed. β -counting was performed using a liquid scintillation counter (Beckman LS 6000TA, Beckman instruments, Inc., Fullerton, CA, USA). RR was calculated as (dpmp—dpm_d_)/ dpm_p_, where dpm_p_ and dpm_d_ are disintegrations per minutes in the perfusate and the dialysate, respectively.

The dialysates from the baseline (140 min), the 20-min work period (160 min), and the recovery period (180, 200 and 220 min) were thawed, centrifugalised, and analysed for the interstitial concentrations of pyruvate, lactate, glutamate, glycerol, and glucose (abbreviated as [pyruvate], [lactate], [glutamate], [glycerol], and [glucose]) with an ISCUSS^flex^ Analyser (CMA Microdialysis, Solna, Sweden; standard range). The detection intervals are as follows: 0.1–12 mmol l^-1^ for [lactate]; 10–1500 μmol l^-1^ for [pyruvate]; 1.0–150 μmol l^-1^ for [glutamate]; 0.1–25 mmol l^-1^ for [glucose]; and 10–1500 mmol l^-1^ for [glycerol]. The interstitial concentrations (Ci) were calculated for the metabolites as Ci = (Cd–Cp) / RR + Cp, where Cd was the dialysate concentration and Cp was the perfusate concentration.

#### Outcomes of the present study

In conclusion, the present study used the following outcomes:

- Interstitial muscle concentrations of lactate, pyruvate, glutamate, glucose and glycerol obtained during miscrodialysis.
- Pain intensity during microdialysis.
- Tests of physical capacity: elbow flexion force right and lefts sides, knee extension strength right and left sides and six-minute walk test (WT-6min).

### Statistics

Statistical analyses were made using IBM SPSS (version 20.0; IBM Corporation, Route 100 Somers, New York, USA) and SIMCA-P+ (version 13.0; Umetrics Inc., Umeå) and p≤0.05 was used as level of significance in all analyses. In text and tables, data are presented as mean ± one standard deviation (± 1SD); in figures, data are presented as mean ± one standard error of the mean (± 1SEM). Generally the present study focused upon between group comparisons but also within group analyses were made. For the overall analyses, we used mean values of each substance, blood flow, and pain intensity throughout the MD session (i.e. time points: 140, 160, 180, 200 and 220 min). To compare groups concerning background data and data from the questionnaire, we used non parametric tests for the classical statistical between group (Mann Whitney U-test) and within group analyses (Wilcoxon Matched-Pair Signed-Rank test, Friedman's 2-Way ANOVA by Ranks test), as the biochemical substances, blood flow variables, and pain intensity were not normally distributed. It was not possible to transform these variables so that e.g. mixed models analyses could be applied using the available statistical package (i.e. SPSS).

Classical statistical methods can quantify the level of individual metabolites but disregard interrelationships between different metabolites [51] and thereby ignore the system-wide aspect of metabolism and algesics. Classical methods assume variable independence when interpreting the results [52]. Multivariate data analyses (MVDA) are capable of handling a number of intercorrelated substances and use advanced principal component analyses (PCA) and Partial Least Squares (PLS) regressions as important tools. When investigating the multivariate correlations between the concentrations of metabolites and pain intensities, PPT and group membership PLS were applied using SIMCA-P+ [53]. Before this analysis, PCA was used to check for multivariate outliers (no multivariate outliers were identified). In the PLS analyses, the mean of each MD session for the biochemical substances and blood flow were used.

*PLS* (i.e., PLS-OPLS/O2PLS) was used for the multivariate regression analysis of PPT, pain intensity (VAS), and group membership (FMS or CON; i.e., *PLS-discriminant analysis* (PLS-OPLS/O2PLS-DA))[53] using the mean interstitial concentrations of the metabolites as regressors. The VIP variable (variable influence on projection) indicates the relevance of each X-variable pooled over all dimensions and the Y-variables–the group of variables that best explains Y. Hence, variables with the highest VIP were most important as regressors. VIP ≥ 1.0 was considered significant. Coefficients (PLS scaled and centred regression coefficients) were used to note the direction of the relationship (positive or negative; in the text reported after the VIP value).

Multiple linear regression (MLR) is an alternative method when regressing pain intensity and PPT, but it assumes that the regressor (X) variables are independent. If multi-colinearity (i.e., high correlations) occurs among the X-variables in MLR, the regression coefficients become unstable and their interpretability breaks down [53]. MLR also assumes that a high subject-to-variables ratio is present (e.g., >5) and such requirements are not required for PLS; in fact, PLS can handle subject-to-variables ratios < 1 and can handle several Y-variables simultaneously [53].

## Results

### Background data

The two groups did not differ in age, blood pressure, or height (**Table 1**). FMS had significantly higher weight and BMI than CON (**Table 1**). FMS subjects had their pain condition for a considerable time. FMS had significantly lower PPT than CON (**Table 1**). Even though the two groups differed significantly on the two scales concerning anxiety and depression (**Table 1**), FMS did not show levels indicating anxiety or depression (i.e. ≥11 on both scales of HADS). Prominent group differences were found for the two quality of life indices: SF36-PCS and SF36-MSC. Both groups had a high participation rate in the 15-week exercise intervention and no significant group difference existed: CON: 93 ± 12% vs. FMS: 92 ± 10%, p = 0.605.

### Strength and endurance before and after the 15-week exercise intervention

Tests of strength and endurance are reported in **Table 2**. No significant group differences in the strength of the lower limbs were found before or after the 15-week exercise intervention (**Table 2**): before: right side (p = 0.196) and left side (p = 0.598); after: right side (p = 0.127) and left side (p = 0.798).

**Table 2:** Results of the static strength of the arm flexors and the knee extensor muscles and the six-minute walk test (WT-6min) before and after the 15-week exercise intervention in patients with fibromyalgia syndrome (FMS) and in healthy controls (CON).

*Group*	***FMS***		*(N = 29)*			***CON***		*(N = 28)*		
*Variables*	*Mean*	*SD*	*Mean*	*SD*	*p-value*	*Mean*	*SD*	*Mean*	*SD*	*p-value*
***Physical capacity***										
Elbow flexion force right (kg)	12.8	4.6	15.2	5.0	.001*	17.0	4.5	16.9	4.5	.876
Elbow flexion force left (kg)	12.8	4.9	15.0	5.4	.003*	16.4	4.1	16.8	3.1	.692
Knee extension strength right (N)	340.8	98.1	353.5	107.6	.387	370.1	85.8	398.8	69.1	.062
Knee extension strength left (N)	321.9	98.6	354.6	102.8	.033*	334.9	84.1	354.7	78.5	.063
WT-6min (m)	567	67	578	59	.049*	627	57	652	51	0.006*

Mean ± one standard deviation (SD) is given before and after the 15-week exercise intervention (15-weeks). For each group, the group analyses (before vs. after; p-value) were reported;

* denotes significant difference.

For between group analyses, see text.

Significant group differences existed in strength of the upper extremities before the 15-week exercise intervention (right side: p = 0.002 and left side: p = 0.006)(**Table 2**). No significant group differences were found after the 15-week exercise intervention (p = 0.136 and p = 0.119) (**Table 2**).

Within group analysis showed that after the 15-week exercise intervention FMS had improved muscle strength in three out of four strength tests (**Table 2**).

Significant group differences existed both before (p = 0.001) and after (p<0.001) the 15-week exercise intervention for WT-6min with longer distances walked in CON than in FMS (**Table 2**). Both CON (p = 0.008) and FMS (p = 0.049) had improved WT-6min after the 15-week exercise intervention (**Table 2**).

### Pain intensity before and after the 15-week exercise intervention

**Fig 3:**
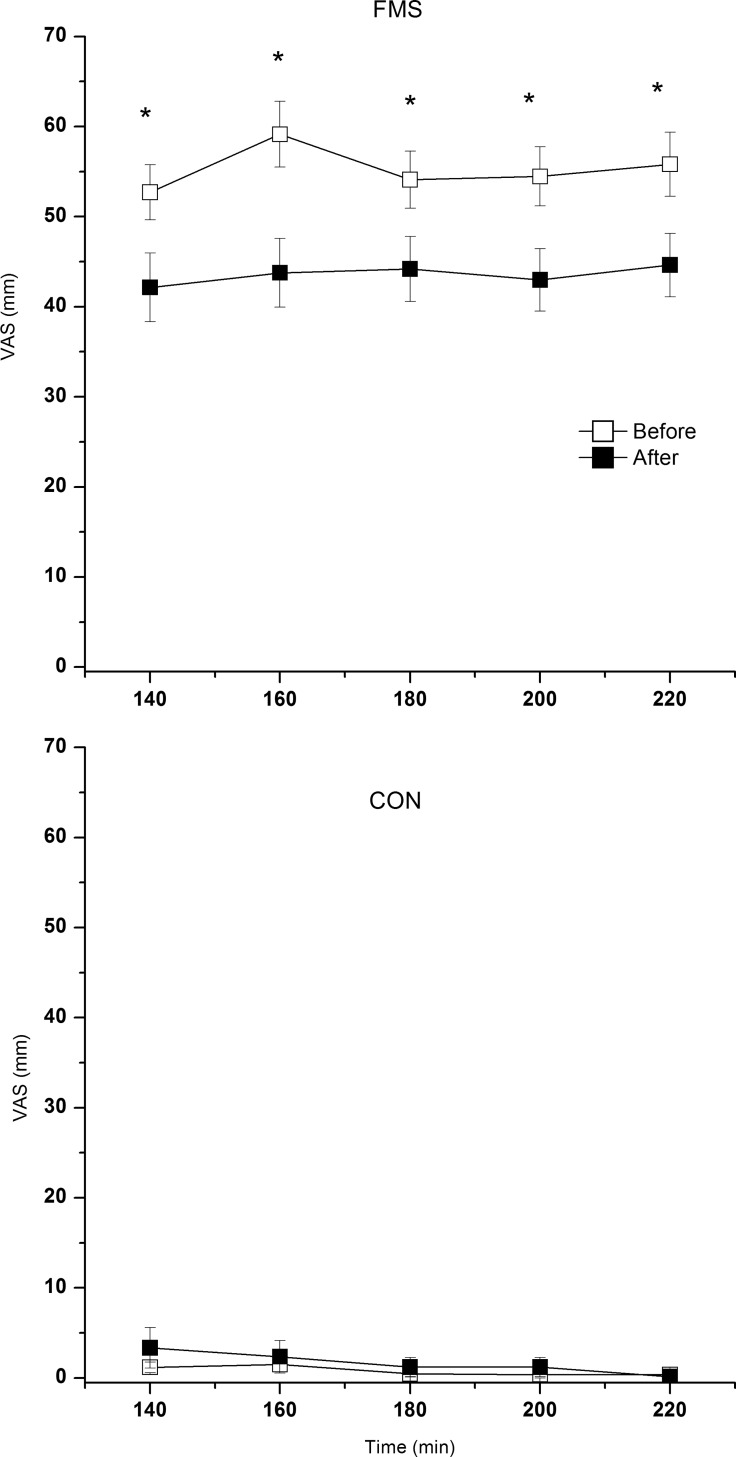
Pain intensity (mean ± SEM) before and after the 15-week exercise intervention in FMS (upper panel) and CON (lower panel) at the time points 140 (baseline), 160 (immediately after the 20-min work period), 180 (recovery), 200 (recovery), and 220 (recovery) min. Note the different y-axis. * denotes significant difference in pain intensity at that time point between before and after the exercise intervention.

As expected, the *mean* pain intensity throughout the MD experiment was significantly higher in FMS than in CON both before and after the 15-week exercise intervention (both p>0.001)(**Fig 3**).

The more detailed analyses revealed that FMS exhibited a significant increase in pain intensity from baseline (i.e., 140 min) to the end of the 20-min work period (i.e., 160 min) at the MD session before (p = 0.006) but not after (p = 0.150) the 15-week exercise intervention (**Fig 3**). No such significant changes were found in CON (p: 0.581–1.000).

The FMS had significantly lower pain intensities at all time points after the 15-week exercise intervention (p: 0.001–0.007)(**Fig 3**); no significant changes existed in CON (p: 0.500–0.866).

### The interstitial concentrations and blood flow at the two MD sessions

In order to get an overview is reported mean values. In the Supporting information (**S1 Appendix** and **S1–S6 Figs**) more detailed analyses of the different time points are presented.

Before the 15-week exercise intervention, there were significant group differences for the mean concentrations of pyruvate and glutamate with significantly higher concentrations in FMS (**Table 3**). A tendency for higher [lactate] in FMS was also observed (p = 0.088). A regression of group membership was done and confirmed in the multivariate context that the mean values of following substances were of significant importance for group membership before the 15-week exercise intervention (R^2^ = 0.11; p<0.05): [pyruvate] (VIP = 1.54+); [glutamate] (VIP = 1.40+); and [lactate] (VIP = 1.17+) (Variables with high VIP values are more important regressors than variables with lower VIP. The sign after the VIP value indicates the direction of the correlation between the regressor and the dependent variable). As a result, [lactate] was also important in the multivariate context.

**Table 3:** The mean concentrations of the five biochemical substances investigated during the two MD sessions–before and after the 15-week exercise intervention–in patients with fibromyalgia syndrome (FMS) and in healthy controls (CON).

*Group*	*FMS*	*n = 29*	*Statistical comparison within FMS*	*CON*	*n = 28*	*Statistical comparison within CON*	*Statistical comparison between groups*
*Substance*	*Mean*	*SD*	*p-value*	*Mean*	*SD*	*p-value*	*p-value*
**Before exercise intervention**							
[glucose] (mmol l^-1^)	8.5	6.4		6.7	3.2		.213
[lactate] (mmol l^-1^)	2.1	0.9		1.6	0.6		.088
[pyruvate] (μmol l^-1^)	82.2	43.9		54.2	33.7		.012*
[glycerol] (mmol l^-1^)	133.7	127.7		116.0	110.6		.690
[glutamate] (μmol l^-1^)	72.3	47.3		47.4	23.0		.014*
**After exercise intervention**							
[glucose] (mmol l^-1^)	6.0	1.7	.039*	6.8	2.3	.838	.191
[lactate] (mmol l^-1^)	1.9	1.1	.381	1.7	0.8	.873	.371
[pyruvate] (μmol l^-1^)	52.0	39.4	.007*	80.9	71.1	.072	.155
[glycerol] (mmol l^-1^)	120.2	91.5	.940	103.6	63.7	.909	.655
[glutamate] (μmol l^-1^)	45.7	23.1	.006*	51.3	28.3	.946	.598

Mean ± one standard deviation (SD) is given before and after the 15-week exercise intervention. Within group analyses (i.e., before vs. after the exercise intervention) and between group analyses (FMS vs. CON) are reported (p-values)

* denotes significant difference.

The FMS showed significant decreases in [glutamate], [pyruvate], and [glucose] after the 15-week exercise intervention (**Table 3**), while no significant changes were found in CON. As a consequence, no significant group differences for the mean concentrations of the five biochemical substances were found after the 15-week exercise intervention (**Table 3**). In order to clarify the importance of these within group differences a multivariate regression of group membership was done using the differences in the biochemical substances as regressors. Hence, this regression confirmed that belonging to the FMS group (R^2^ = 0.31; p<0.05) was associated with changes (differences between concentration before and after 15-week exercise intervention) in [pyruvate] (VIP = 1.64+) and in [glutamate] (VIP = 1.31+).

No significant differences in *mean* blood flow between the two groups were found at the two sessions–before: CON: 0.64 ± 0.17 vs. FMS: 0.63 ± 0.17; p = 0.549; after CON: 0.63 ± 0.13 vs. FMS: 0.58 ± 0.17; p = 0.231. No significant within group changes (i.e., before vs. after the 15-week exercise intervention) in mean blood flow were found in either the CON (p = 0.707) or the FMS (p = 0.113).

### Regressions of pain intensity, PPT, psychological status and physical tests

Regressions were made in order to explore if the concentrations of the biochemical substances correlated with pain aspects and physical performance; regression of pain intensity was not done in CON as the subjects in this group had no chronic pain.

No significant regressions were obtained when regressing mean pain intensity using the mean values of the five biochemical substances and blood flow in FMS before and after the 15-week exercise intervention. A significant regression was found when regressing the difference in mean pain intensity (before exercise minus after the 15-week exercise intervention) using the changes in biochemical substances and blood flow (before minus after the 15-week exercise intervention) (R^2^ = 0.13; p<0.05) and the following variables were significant: change in [pyruvate] (VIP = 1.72-) and change in [glucose] (VIP = 1.02-).

No significant regressions were obtained when regressing PPT or psychological status (both subscales of HADS) in each group separately using the mean values of the biochemical substances and blood flow as regressors.

It was not possible to significantly regress differences in the physical tests (i.e., after minus before the 15-week exercise intervention) using the differences in the biochemical concentrations and blood flow (before exercise intervention minus after the exercise intervention) in all subjects taken together or in either of the two groups separately.

## Discussion

This study produced three major and novel results:

- FMS was associated with higher [glutamate], [pyruvate], and [lactate].
- After the 15-week exercise intervention, the FMS subjects had a decrease in pain intensity, decreases in [glutamate], [pyruvate], and [glucose], and an increase in muscular fitness.
- The decrease in pain intensity in FMS after the exercise intervention was significantly correlated with the decreases in [pyruvate] and [glucose].

### Biochemical substances and blood flow before the 15-week exercise intervention

Four earlier MD studies have investigated muscle algesic and/or metabolic situations in FMS [28–30,32]. The regression of group membership confirmed that [glutamate] and [pyruvate] were important and [lactate] was also important when considering system-wide aspects. Although only three substances had increased concentrations, a relatively massive nociceptive inflow to the CNS may be present if similar alterations are present in several muscles. Muscle nociceptive inputs are believed to be important for maintaining central hyperexcitability [1]. Moreover, although the levels of glutamate, pyruvate and glucose had decreased after the 15-week exercise intervention and the decreases in two of these substances correlated with decreases in pain intensity it must be noted that FMS was not pain free after the exercise intervention. Taken together this may indicate that other peripheral and CNS factors also are important for pain aspects in FMS. A proteomic study of trapezius dialysate in CWP/FMS found that 1/3 of the proteins were significantly up or down regulated [54], which could reflect nociceptive processes, deconditioning, etc. The present and other MD studies pinpoint the need to move from a search of single molecules to a more systematic approach with respect to metabolites, algesics, and non-inflammatory substances [27].

### Glutamate

The finding that increased [glutamate] was present before the exercise intervention (**Table 3** and **S1 Fig**) agrees with findings from a study of the trapezius muscle of CWP/FMS [30]. However, in another study [glutamate] during rest did not differ between FMS and controls [29]. The difference in results may be related to methodological differences. Higher [glutamate] in muscles of local/regional pain conditions have also been found [26,27,55], although there is not total consensus [56]. Glutamate can be released from peripheral afferent neurons, resulting in excitation and sensitization of the same or adjacent neurons [55,57,58]. Glutamate acts via N-methyl-D-aspartate (NMDA), AMPA and possibly TRPV1 receptors [59–61]. When NMDA receptors are activated by glutamate, Nitric Oxide (NO)–a reactive oxygen species (ROS)–is generated [62] and can contribute to peripheral hyperalgesia [63,64]. Glutamate can induce experimental pain [55] and hypertonic saline infusion into the masseter and biceps muscles leads to release of glutamate [65,66]. Thus, human studies of e.g. FMS have found that glutamate is a peripheral algesic and contributes to chronic pain.

### Lactate, pyruvate, and blood flow

Lactate is produced during anaerobic and aerobic conditions and can be metabolized in the same cell or transported to other cells for metabolic use [67–73]. [Pyruvate] was significantly increased in FMS and [lactate] was multivariately important (**Table 3**). Increased [lactate] and [pyruvate] were found in the trapezius muscle of FMS [29]; this observation was also confirmed for [lactate] in FMS/CWP [30]. Hence, most studies of FMS show significant alterations in these substances.

Increased [lactate] and [pyruvate] can induce ROS [74–76], which may interact with nociception [77]. On the other hand, it has been suggested that pyruvate is an endogenous antioxidant [78–80]. A human study showed that a combination of metabolites is needed to activate nociceptors [81]. Lactic acid is dissociated at body pH [82]. Lactate together with adenosine triphosphate (ATP) facilitate the response of acid-sensing ion channel 3 (ASIC-3) to low pH [83–86]. Other receptors for low pH are TRPV1 [87], TRPV4 [61], TRPC4, and TRPC5 [88].

Increased [pyruvate] and [lactate] cannot be explained by insufficient oxygen supply since no group difference in blood flow existed either in the present cohort (**S6 Fig**) or the FMS/CWP cohort [30]; FMS studies using other techniques are inconsistent [89–92]. Altered activation patterns of the fibromyalgic muscle [93–96] may indicate muscular over-activity and thereby higher [lactate] and possibly [pyruvate] [97,98]. Mitochondrial insufficiency is another explanation for increased [lactate] and possibly [pyruvate] [99,100]. Some studies report increased frequencies of muscle fibres with alterations in mitochondrial distribution [101–103]. Decreased levels of ATP and phosphocreatine in vastus lateralis of FMS could indicate mitochondrial insufficiency [104,105]. Mitochondria dysfunction may be linked to the pain per se, but it may also be due to e.g. physical inactivity [99]. In FMS/CWP, muscular fitness is reduced [101,106]; a tendency also noted in this cohort of FMS.

### Glycerol and Glucose

Glycerol is a component of cell membranes [107] and insertion of catheters may cause an increase in [glycerol]. No difference in tissue reactions existed between the two groups (**Table 3**), but on the other hand it could have occurred during the trauma phase (not analysed in the present study). An increase in [glycerol] together with increases in [glutamate] and [serotonin] after hypertonic saline infusion have been reported [65], but their relationship to pain is unclear.

Correlations between elevated [glucose] in plasma and chronic pain have been reported [108,109], while PPT correlated positively with hyperglycemia in healthy men [110]. As in our study, one study found no significant group differences in [glucose] in chronic trapezius myalgia of low severity [111]. The decrease in [glucose] after the exercise intervention may be due to decreased sympathetic activity in FMS [112–114]. Unfortunately indicators of sympathetic drive was not registered using e.g. heart rate analysis in the present study.

### The effects of the 15-week exercise intervention

Earlier studies of exercise studies concerning chronic pain including FMS and CWP [18–21,115] also found decreased pain intensity. In addition, our study found improvements in 4/5 fitness tests in FMS. Our study also found that the CON exhibited significant improvement in WT-6min and the strength of the knee extensors nearly reached significant improvements.

The mechanisms for the positive effects of exercise in chronic pain are unclear as mentioned earlier, which hinders the development of exercise interventions aimed at diminishing pain. However, our study found that exercise at least partially is linked to periphearl effects in FMS and that glutamate and pyruvate normalized simultaneous with a decrease in pain intensity. One earlier project has used MD to evaluate the effects of exercise and reported decreased [glutamate] and [substance P] together with increased [beta-endorphin] and [cortisol] in the trapezius of subjects with chronic neck-shoulder pain [35]. Moreover, the effects on two NAEs differed with respect to type of exercise intervention [116]. Hence, two chronic pain conditions showed peripheral changes and decreased [glutamate] after exercise interventions. Karlsson et al. reported that improvements in pain intensity were associated with a decrease in [glutamate] and in an increase in cortisol [35]. In this study, improvements in pain intensity were associated with a decrease in [pyruvate]. The reason for this difference is unclear but may be linked to different pain conditions, muscles, and/or exercise interventions were investigated. Although a significant relationship existed, the explained variation was low (R^2^ = 0.13), indicating that other substances may be more important with respect to pain intensity. The possibility that the changes in metabolites mainly was linked to improvements in muscular fitness was not confirmed.

### Strengths and Limitations

The number of subjects in each group was higher than in other MD studies of FMS. FMS shows considerable heterogeneity, so it is difficult to generalize our results to FMS in the population. Both in this and other FMS cohorts investigated by our group, a low severity of comorbidities was noted and there is a risk that the severe cases of FMS were excluded due to nature of the studies. The design of the 15-week exercise intervention made it impossible to determine when the changes occurred. It is important to elucidate when improvements in pain intensity, physical performance, and biochemical substances occur to optimize exercise interventions. Significant differences in BMI and weight existed between the two groups and it cannot be completely ruled out that the normalization of metabolites in FMS was due to improved fitness after the 15-week exercise intervention. Against this can be argued that we found no significant correlations between changes in physical performance and changes in metabolites. In the present study was made no corrections for multiple statistical testing since the tests were not statistically independent, which is obvious when analysing the different time points e.g. for [glutamate] (**S1 Fig**) between the two groups. Using Bonferroni corrections in such situations will lead to too strong corrections and increase the risk for false negative results. The use of MVDA in the present study mitigates but does not completely eliminate the problem of multiple testing.

### Conclusions

FMS had significantly increased [glutamate], [pyruvate] and [lactate] in the vastus lateralis before the exercise intervention. The increased levels of [glutamate] and [pyruvate] decreased after the 15-week exercise intervention and no group differences were noted. The decrease in pain intensity in FMS after the 15-week exercise intervention was significantly correlated with the decreases in [pyruvate] and [glucose]. As in earlier studies, our study indicates that peripheral muscle alterations may contribute to pain in FMS patients. These alterations can be normalized and pain intensity can be decreased (but not abolished) after an exercise intervention. In addition, the effects of exercise in FMS are partially peripheral.

## Supporting Information

S1 Checklist TREND Statement Checklist. (PDF)

S1 Appendix Interstitial concentrations of the biochemical substances and blood flow at the five time points. In this appendix are MD results reported more in detail i.e. at the five time points during the MD sessions before and after the intervention in the two group of subjects.
(PDF)

S1 Fig Interstitial concentration of glutamate. Interstitial concentration of glutamate (mean ± SEM; μmol l^-1^) before and after the 15-week exercise intervention in FMS (upper panel) and CON (lower panel) at the time points 140 (baseline), 160 (immediately after 20-min work period), 180 (recovery), 200 (recovery), and 220 (recovery) min. * denotes significant difference in interstitial concentration of glutamate at that time point between before and after the exercise intervention.
(PDF)

S2 Fig Interstitial concentration of lactate. Interstitial concentration of lactate (mean ± SEM; mmol l^-1^) before and after the 15-week exercise intervention in FMS (upper panel) and CON (lower panel) at the time points 140 (baseline), 160 (immediately after 20-min work period), 180 (recovery), 200 (recovery), and 220 (recovery) min. * denotes significant difference interstitial concentration of lactate at that time point between before and after the exercise intervention.
(PDF)

S3 Fig Interstitial concentration of pyruvate. Interstitial concentration of pyruvate (mean ± SEM; μmol l^-1^) before and after the 15-week exercise intervention in FMS (upper panel) and CON (lower panel) at the time points 140 (baseline), 160 (immediately after 20-min work period), 180 (recovery), 200 (recovery), and 220 (recovery) min. * denotes significant difference in interstitial concentration of pyruvate at that time point between before and after the exercise intervention.
(PDF)

S4 Fig Interstitial concentration of glycerol. Interstitial concentration of glycerol (mean ± SEM; mmol l^-1^) before and after the 15-week exercise intervention in FMS (upper panel) and CON (lower panel) at the time points 140 (baseline), 160 (immediately after 20-min work period), 180 (recovery), 200 (recovery), and 220 (recovery) min. * denotes significant difference in concentration of glycerol at that time point between before and after the exercise intervention.
(PDF)

S5 Fig Interstitial concentration of glucose. Interstitial concentration of glucose (mean ± SEM; mmol l^-1^) before and after the 15-week exercise intervention in FMS (upper panel) and CON (lower panel) at the time points 140 (baseline), 160 (immediately after 20-min work period), 180 (recovery), 200 (recovery), and 220 (recovery) min. * denotes significant difference in concentration of glucose at that time point between before and after the exercise intervention.
(PDF)

S6 Fig Blood flow. Blood flow of the trapezius (mean ± SEM; arbitrary units) before and after the 15-week exercise intervention in FMS (upper panel) and CON (lower panel) at the time points 140 (baseline), 160 (immediately after 20-min work period), 180 (recovery), 200 (recovery), and 220 (recovery) min. * denotes significant difference in blood flow at that time point between before and after the exercise intervention.
(PDF)

S1 Protocol Ethical application including approval (in Swedish). (PDF)
