# Supplementary material for: Increased Interstitial Concentrations of Glutamate and Pyruvate in Vastus Lateralis of Women with Fibromyalgia Syndrome Are Normalized after an Exercise Intervention – A Case-Control Study
Source: PLoS One. 2016 Oct 3;11(10):e0162010. doi: 10.1371/journal.pone.0162010 (PMC5047648; doi:10.1371/journal.pone.0162010)
Supplement: S1 Appendix — In this appendix are MD results reported more in detail i.e. at the five time points during the MD sessions before and after the intervention in the two group of subjects. (PDF) [file pone.0162010.s002.pdf]

S1 Appendix
Interstitial concentrations of the biochemical substances and blood flow at the five time points

In this appendix are MD results reported more in detail i.e. at the five time points during the MD sessions before and after the intervention in the two group of subjects (**S1-6 Figs**).

Glutamate

In both groups and at both MD sessions, [glutamate] changed over time – i.e., increases at 160 and/or 180 minutes (CON: before p=<0.001, after p=0.005, FMS before p=<0.001, after p<0.001) (**S1 Fig**).

The statistical comparisons of before versus after the exercise intervention in FMS (**S1 Fig**) showed significantly lower [glutamate] after the exercise intervention at all but one of the time points: 140 min: p=0.068 (ns); 160 min: p=0.034; 180 min: p=0.013; 200 min: p=0.131 (ns); and 220 min: p=0.010. The CON displayed a significant difference in [glutamate] at 140 min (p=0.043), but no other significant differences (p: 0.052-0.665).

Lactate

In FMS, there were changes in [lactate] over time after the exercise intervention – i.e., increases at 160 min (before: p=0.058; after: p=0.001)(**S2 Fig**). In CON, there were no significant changes in [lactate] over time at the two MD sessions (before: p=0.071 (ns); after: p=0.242 (ns)). No significant differences in [lactate] between MD before and after the exercise intervention in FMS (P: 0.163-0.683) or in CON (p: 0.109-0.792) were found at any time points (**S2 Fig**).

Pyruvate

For both FMS and CON, there were significant changes in [pyruvate] over time at both MD sessions – i.e., increases at 160 and/or 180 min (FMS: before: p=0.013, after: p=0.007; CON: before: p=0.015, after: p<0.001)(**S3 Fig**). Significant differences in [pyruvate] between the two MD sessions in FMS were found at all time points (**S3 Fig**): 140 min: p=0.001; 160 min: p=0.003; 180 min: p=0.062 (ns);

200 min: p=0.028, and 220 min: p=0.001. The concentrations were lower after the 15-week intervention, although the CON showed a significant difference in [pyruvate] at 160 min (p=0.028) but not at the other time points (p: 0.163-0.264).

Glycerol

In FMS, there were significant changes in [glycerol] over time at both MD sessions – i.e., increases at 160 and/or 180 min (before: p<0.001, after: p=0.001) (**S4 Fig**). In CON, there were significant changes in [glycerol] over time after the exercise intervention but not before (before: p=0.127 (ns); after: p<0.001). When comparing the two MD sessions (**S4 Fig**), significant differences in [glycerol] were evident at 180 min (p=0.027) in FMS, but not at the other time points (p: 0.179-0.962). The CON exhibited significant differences in [glycerol] at 160 min (p=0.023) and 220 min (p=0.032), but not at the other time points (p: 0.187-0.597).

Glucose

In FMS, there were significant changes in [glucose] over time at both MD sessions (before: p=0.010; after: p=0.012) (**S5 Fig**). In CON, there were significant changes in [glucose] over time at the MD session after the exercise intervention but not before (before: p=0.271 (ns); after: p<0.001). The FMS also exhibited significant differences before vs. after the exercise intervention (**S5 Fig**) in [glucose] at two time points (180 min: p=0.011; 200 min: p=0.034), but the other time points were not significantly different between the two MD sessions (p: 0.265-0.317). No significant differences were found in CON (p: 0.107-0.891).

Blood flow

The detailed analysis of blood flow all time points revealed that in both groups and at both MD sessions there were changes over time (i.e., throughout MD) in blood flow (all p=<0.001) (**S6 Fig**). As in the analyses of mean blood flow, we found no significant group differences at the five time points between CON and FMS at either of the two MD sessions (p: 0.318-0.949). No significant within group differences (i.e., before vs. after) existed at any time point in CON (p: 0.219-0.927) or in FMS (P: 0.065-0.297).
