# Supplementary figures and images for: Increased Interstitial Concentrations of Glutamate and Pyruvate in Vastus Lateralis of Women with Fibromyalgia Syndrome Are Normalized after an Exercise Intervention – A Case-Control Study
Source: PLoS One. 2016 Oct 3;11(10):e0162010. doi: 10.1371/journal.pone.0162010 (PMC5047648; doi:10.1371/journal.pone.0162010)

S1 Fig

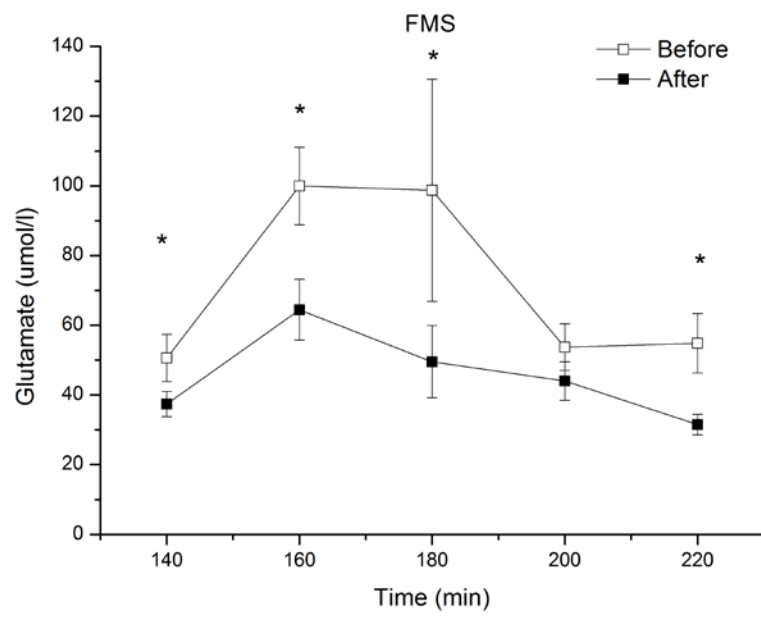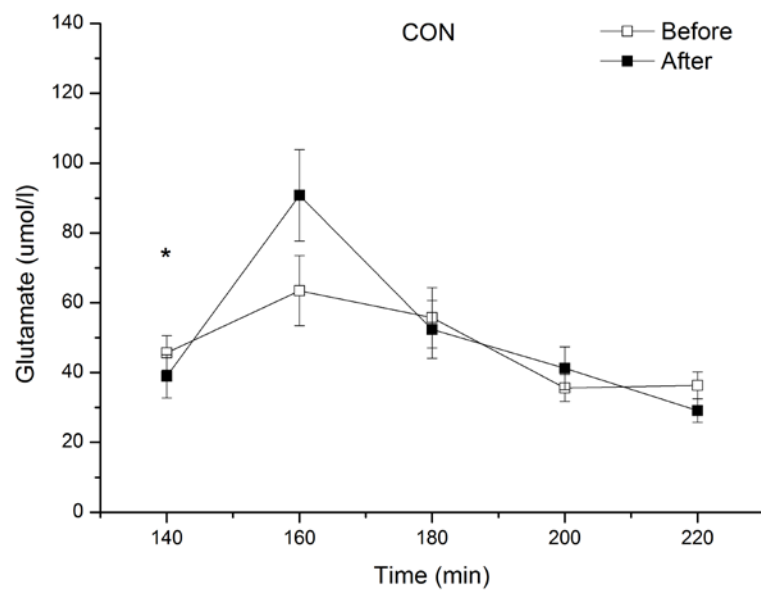

Supplement: S1 Fig — Interstitial concentration of glutamate (mean ± SEM; μmol l-1) before and after the 15-week exercise intervention in FMS (upper panel) and CON (lower panel) at the time points 140 (baseline), 160 (immediately after 20-min work period), 180 (recovery), 200 (recovery), and 220 (recovery) min. * denotes significant difference in interstitial concentration of glutamate at that time point between before and after the exercise intervention. (PDF) [file pone.0162010.s003.pdf]

S2 Fig

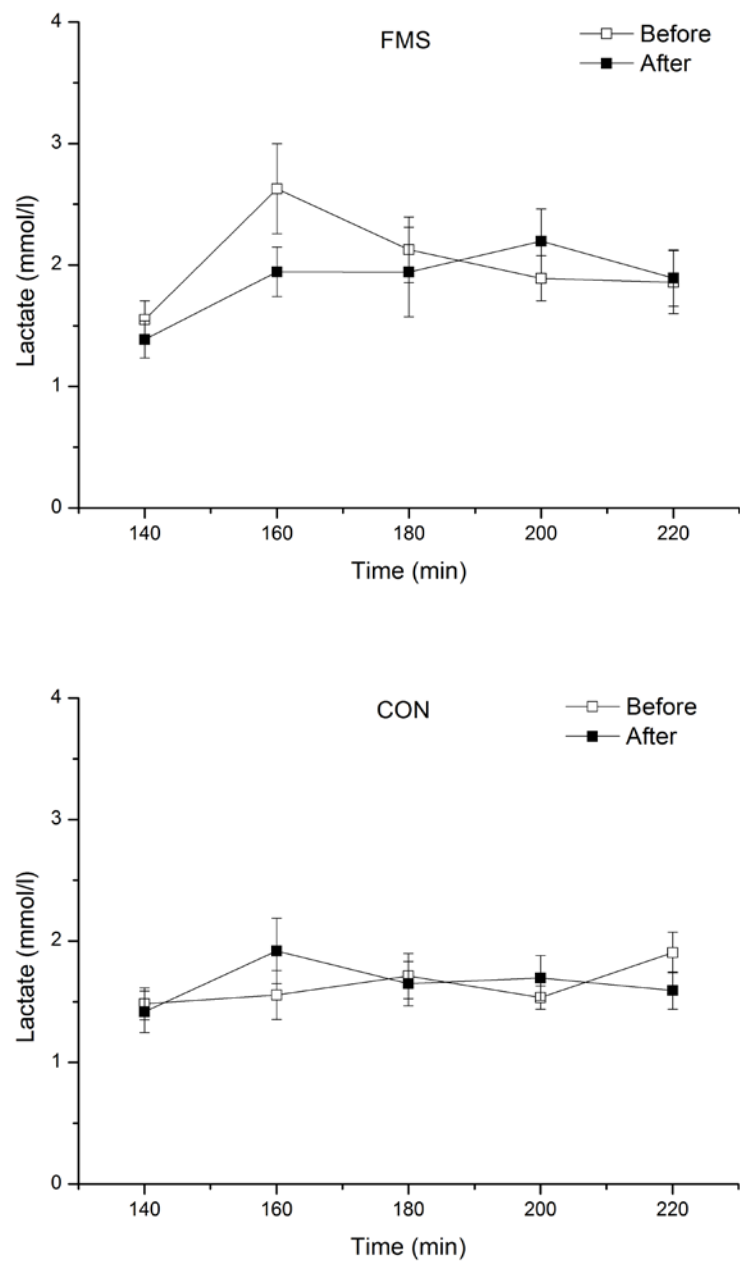

Supplement: S2 Fig — Interstitial concentration of lactate (mean ± SEM; mmol l-1) before and after the 15-week exercise intervention in FMS (upper panel) and CON (lower panel) at the time points 140 (baseline), 160 (immediately after 20-min work period), 180 (recovery), 200 (recovery), and 220 (recovery) min. * denotes significant difference interstitial concentration of lactate at that time point between before and after the exercise intervention. (PDF) [file pone.0162010.s004.pdf]

S3 Fig

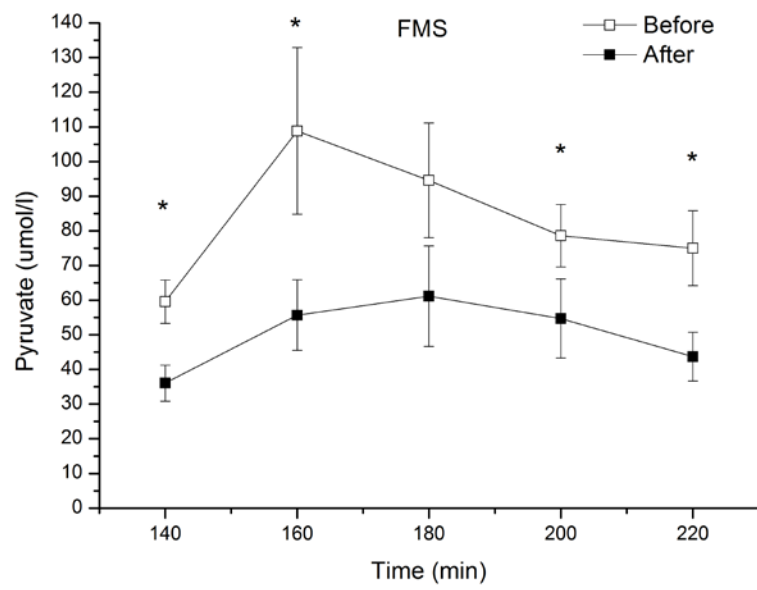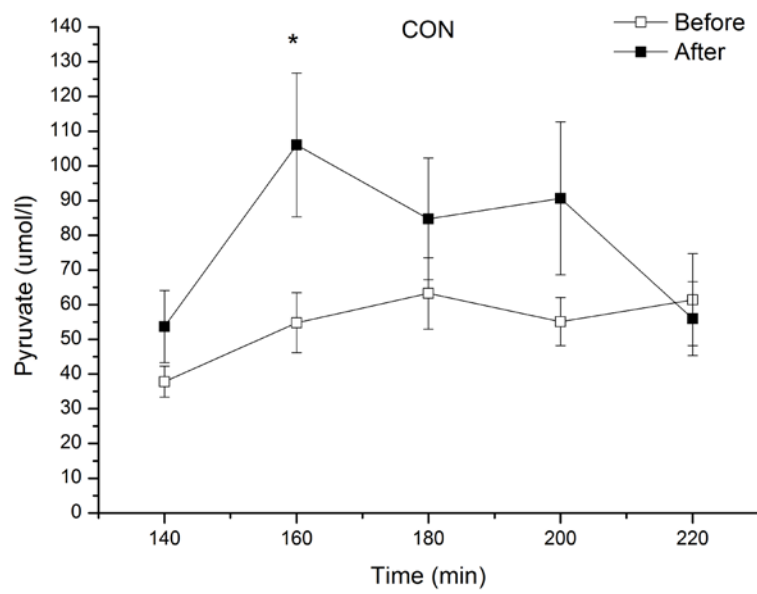

Supplement: S3 Fig — Interstitial concentration of pyruvate (mean ± SEM; μmol l-1) before and after the 15-week exercise intervention in FMS (upper panel) and CON (lower panel) at the time points 140 (baseline), 160 (immediately after 20-min work period), 180 (recovery), 200 (recovery), and 220 (recovery) min. * denotes significant difference in interstitial concentration of pyruvate at that time point between before and after the exercise intervention. (PDF) [file pone.0162010.s005.pdf]

S4 Fig

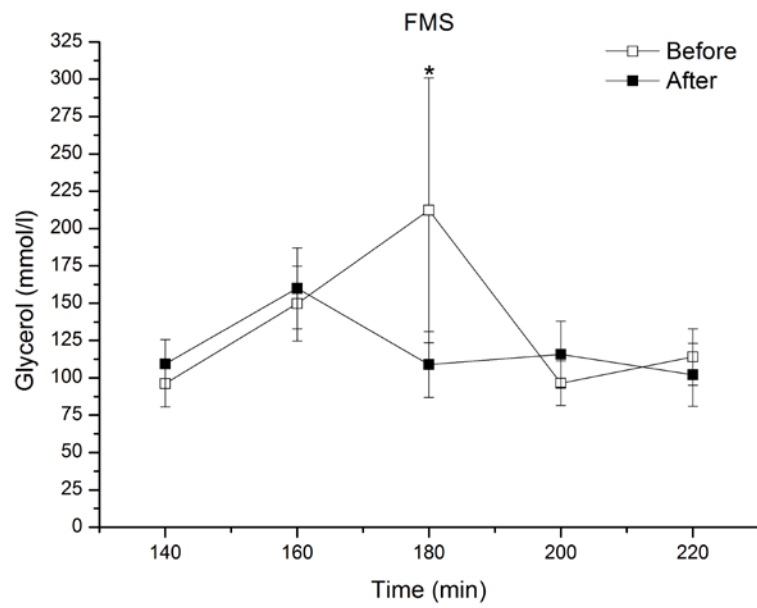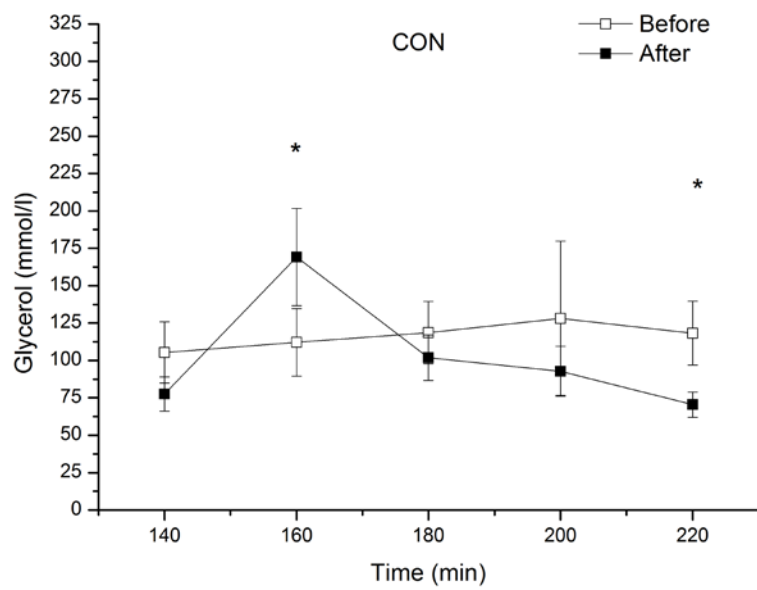

Supplement: S4 Fig — Interstitial concentration of glycerol (mean ± SEM; mmol l-1) before and after the 15-week exercise intervention in FMS (upper panel) and CON (lower panel) at the time points 140 (baseline), 160 (immediately after 20-min work period), 180 (recovery), 200 (recovery), and 220 (recovery) min. * denotes significant difference in concentration of glycerol at that time point between before and after the exercise intervention. (PDF) [file pone.0162010.s006.pdf]

S5 Fig

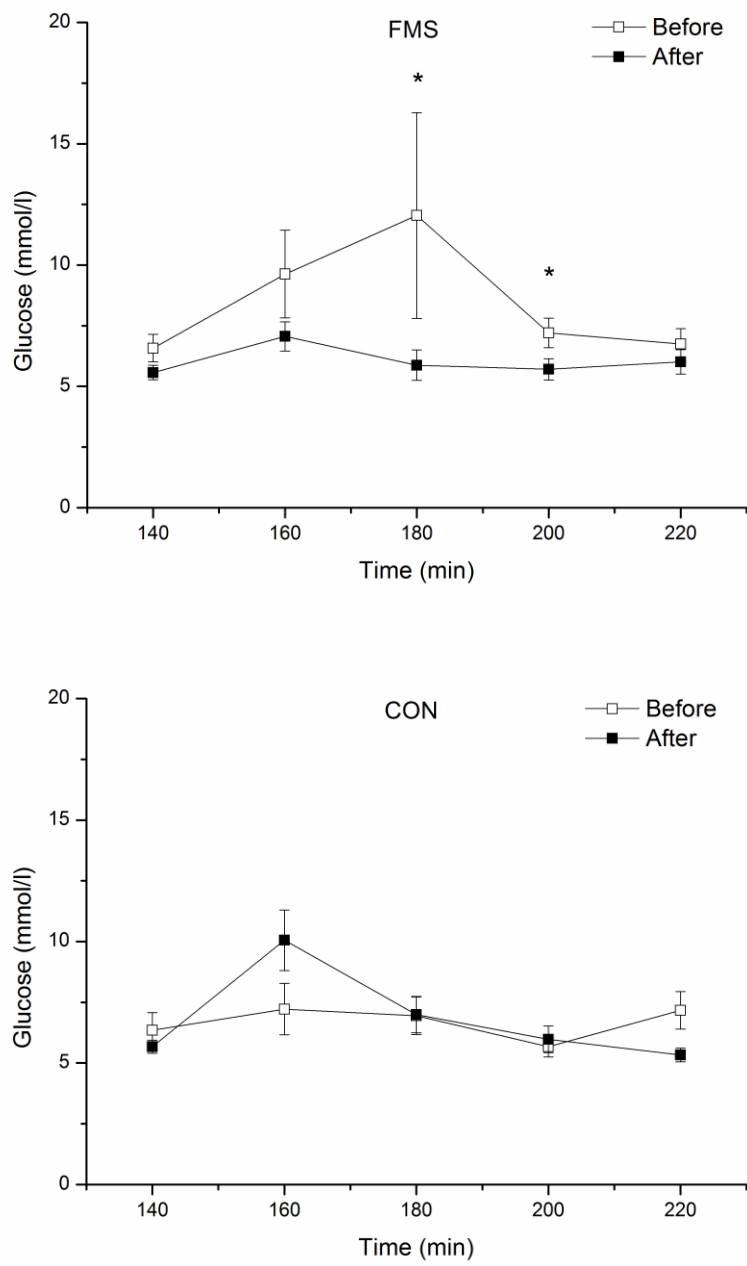

Supplement: S5 Fig — Interstitial concentration of glucose (mean ± SEM; mmol l-1) before and after the 15-week exercise intervention in FMS (upper panel) and CON (lower panel) at the time points 140 (baseline), 160 (immediately after 20-min work period), 180 (recovery), 200 (recovery), and 220 (recovery) min. * denotes significant difference in concentration of glucose at that time point between before and after the exercise intervention. (PDF) [file pone.0162010.s007.pdf]

S6 Fig

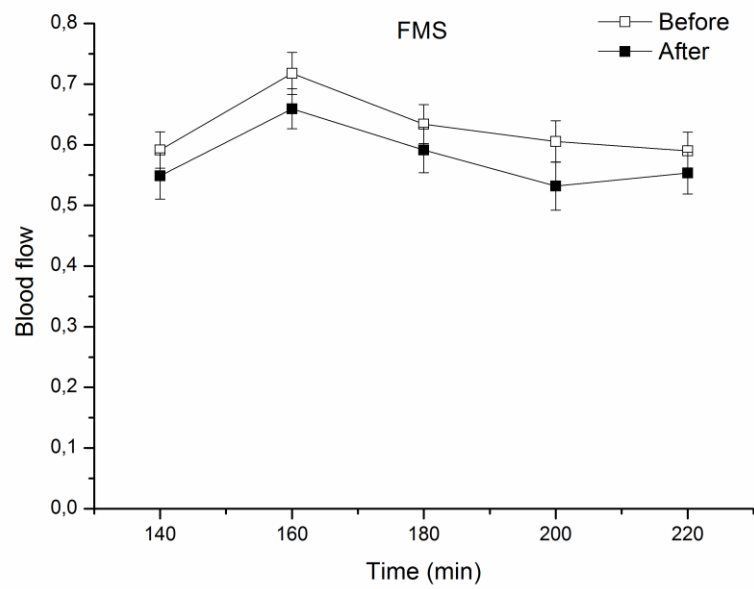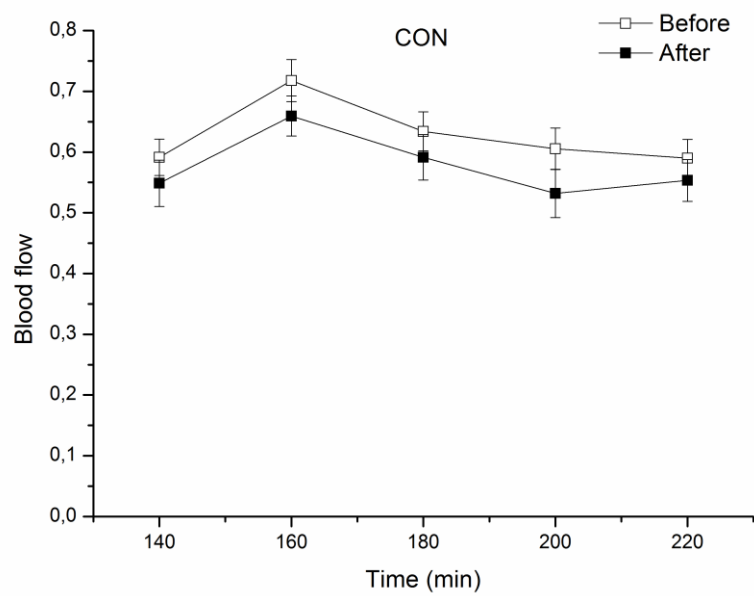

Supplement: S6 Fig — Blood flow of the trapezius (mean ± SEM; arbitrary units) before and after the 15-week exercise intervention in FMS (upper panel) and CON (lower panel) at the time points 140 (baseline), 160 (immediately after 20-min work period), 180 (recovery), 200 (recovery), and 220 (recovery) min. * denotes significant difference in blood flow at that time point between before and after the exercise intervention. (PDF) [file pone.0162010.s008.pdf]
