# Supplementary material for: Increased Interstitial Concentrations of Glutamate and Pyruvate in Vastus Lateralis of Women with Fibromyalgia Syndrome Are Normalized after an Exercise Intervention – A Case-Control Study
Source: PLoS One. 2016 Oct 3;11(10):e0162010. doi: 10.1371/journal.pone.0162010 (PMC5047648; doi:10.1371/journal.pone.0162010)
Supplement: S1 Protocol — (PDF) [file pone.0162010.s009.pdf]

EPN REGIONALA ETIKPRÖVNINGSNÄMNDEN STOCKHOLM

FÖR KÄNNEDOM

PROTOKOLL 2010/3:7
2010-08-18

Sammanträde i Stockholm

Avdelning 3

Ordförande
Håkan Julius

Ledamöter med vetenskaplig kompetens
Hans Glaumann, vetenskaplig sekreterare *(infektionssjukdomar)*
Ulf Adamson *(medicin, endokrinologi)*, deltar ej i ärendena 2010/1071, 2010/1092, 2010/1124-1127, 2010/1129-1131, 2010/1133, 2010/1147, 2010/1153 och 2010/1168
Björn Almé *(psykiatri)*
Maria Ankarcrona *(geriatrik)*, deltar i ärendena 2010/1092, 2010/1124-1127, 2010/1129-1131, 2010/1133, 2010/1147, 2010/1153 och 2010/1168
Maria Feychting *(miljömedicin, epidemiologi)*
Niels Lynöe *(socialmedicin, omvårdnad)*
Gianni Celsi *(barnmedicin)*, deltar i ärendena 2010/998, 2010/1062 och 2010/1077-1078
Mårten Rosenqvist *(kardiologi)*
Björn Wrangsjö *(barn- och ungdomspsykiatri)*

Ledamöter som företräder allmänna intressen
Birgitta Mörk
Bengt Gustafsson
Gösta Johansson
Ulf Uebel
Lars Åstrand

Övriga närvarande
Anne Manninen, administrativ sekreterare
Stefan Borg, ersättare för Hans Glaumann

---

§ 1 Ordföranden förklarar sammanträdet öppnat.

§ 2 Ledamoten Mårten Rosenkvist ifrågasätter om inte s.k. kvalitetssäkrings-studier eller studier utan egentliga etiska problem skulle kunna hanteras av etikprövningsnämnden med ett enklare förfarande, t.ex. att dessa delegeras till ordföranden att fatta beslut efter föredragning av den vetenskapliga sekreteraren. Ordföranden lovade att föra ärendet vidare till övriga ordföranden i samband med ett nämndledningsmöte.

§ 3 Den administrativa sekreteraren anmäler att den vetenskaplige sekreteraren sedan föregående möte den 9 juni 2010 fattat beslut i 34 ärenden som avser ändring av godkännande.

Adress	Besöksadress	Telefon	Fax	E-post	Hemsida
FE 289<br>171 77 Stockholm	Nobels väg 12 A<br>171 65 Solna	08-524 800 00	08-524 866 99	kansli@stockholm.epn.se	www.epn.se

§ 4 Ansökningar om etisk granskning av forskningsprojekt, se **Bilaga**.

§ 5 Ordföranden meddelar att nästa sammanträde i avdelning 3 äger rum onsdagen den 15 september 2010.

§ 6 Ordföranden förklarar mötet avslutat.

Håkan Julius
Ordförande

Hans Glaumann
Protokollförare, vetenskaplig sekreterare

------------- Utdrag ur protokoll från sammanträde den 18 augusti 2010 i avdelning 3 -------------

Diarienummer
Föredragande

2010/1121-31/3
Björn Almé

Sökande: Karolinska Institutet
Behörig företrädare: Jan Hillert
Projekt: Effekten av fysisk träning på sjukdomsmekanismer och symtom vid fibromyalgi.
Forskare som genomför projektet: Eva Kosek

BESLUT

Nämnden begär att sökanden kompletterar ansökan enligt följande:

1. Forskningspersonsinformation till patienter med fibromyalgi saknas och ska inskickas.
2. Nämnden sätter i fråga om inte även kontrollpersonerna ska genomgå s.k. funktionell magnetisk resonans imaging (fMRI) för att erhålla säkrare resultat.
3. Under 2:5 i ansökan anges att personuppgiftsansvarig är resp. biobank. Det torde snarare vara resp. universitetssjukhus.

Begärda kompletteringar skall i **ett exemplar** ha inkommit till nämnden senast **20 september 2010**. Efter den tidpunkten kommer ärendet att tas upp till prövning i befintligt skick.

Nämnden lämnar över till den vetenskapliga sekreteraren efter samråd med föredraganden att avgöra ärendet sedan kompletteringar inkommit.

Kopians överensstämmelse med originalet intygas:
[signature]
ANNE MANNINEN

GODKÄNNES Dat. 2010-09-14
[signature]
Hans Glaumann
Regionala etikprövningsnämnden i Stockholm

Beslut expedierat till behörig företrädare.
Kopia för kännedom till ansvarig forskare.

Att utdraget överensstämmer med originalet intygar:
[signature]
Anne Manninen, administrativ sekreterare
/expedierat 2010-08-26

Regionala etikprövningsnämnden

Stockholm 2010-09-10

Föredragande Björn Almé

Nobels väg 12 A

171 65 Solna

Kompletteringar till etikansökan 2010/1121-31/3 "Effekten av fysisk träning på sjukdomsmekanismer vid fibromyalgi"

1) ***Forskningspersonsinformation till patienter med fibromyalgi.***
   Vi ber om ursäkt att denna föll bort vid första inskicket, informationen bifogas nu.
2) ***fMRI av kontrollpersoner.***
   Vi hade tänkt använda fMRI data från 16 friska kontrollpersoner som undersökts med samma paradigm i tidigare studie (baseline). Emellertid kan vi då inte uttala oss om vad träning har för effekt på smärtmoduleringen hos friska. För att erhålla säkrare resultat har vi följt etiknämndens rekommendationer och nu lagt till fMRI undersökning även av kontrollpersoner före och efter träning. Försökspersonsarvodet för dessa har ändrats till 3 500kr (i enlighet med FM patienterna).
3) ***Personuppgiftsanavarig.***
   Under punkt 2:5 anges resp. universitetssjukhus som personuppgiftsanavarig.
4) ***Komplettering av en klinik i Göterborg.***
   Eftersom vissa patienter i Göteborg kan komma att rekryteras från NärRehab Alingsås har vi lagt till denna klinik under övriga medverkande, Göteborg. Signerat intyg av verksamhetschef bifogas.

Med vänliga hälsningar,

Eva Kosek, Docent, Överläkare
Insitutionen för Klinisk Neurovetenskap
Karolinska Institutet
Retzius väg 8
171 77 Stockholm

FÖR KÄNNEDOM

Regionala etikprövningsnämnden i Stockholm
Protokoll 2010/3:7

-------------- Utdrag ur protokoll från sammanträde den 18 augusti 2010 i avdelning 3 --------------

Diarienummer
Föredragande

2010/1121-31/3
Björn Almé

Sökande: Karolinska Institutet
Behörig företrädare: Jan Hillert
Projekt: Effekten av fysisk träning på sjukdomsmekanismer och symtom vid fibromyalgi.
Forskare som genomför projektet: Eva Kosek

BESLUT

Nämnden begär att sökanden kompletterar ansökan enligt följande:

1. Forskningspersonsinformation till patienter med fibromyalgi saknas och ska inskickas.
2. Nämnden sätter i fråga om inte även kontrollpersonerna ska genomgå s.k. funktionell magnetisk resonans imaging (fMRI) för att erhålla säkrare resultat.
3. Under 2:5 i ansökan anges att personuppgiftsansvarig är resp. biobank. Det torde snarare vara resp. universitetssjukhus.

Begärda kompletteringar skall i **ett exemplar** ha inkommit till nämnden senast **20 september 2010**. Efter den tidpunkten kommer ärendet att tas upp till prövning i befintligt skick.

Nämnden lämnar över till den vetenskapliga sekreteraren efter samråd med föredraganden att avgöra ärendet sedan kompletteringar inkommit.

Beslut expedierat till behörig företrädare.
Kopia för kännedom till ansvarig forskare.

Att utdraget överensstämmer med originalet intygar:

Anne Manninen, administrativ sekreterare
/expedierat 2010-08-26

Ordförande
Håkan Julius

Ledamöter med vetenskaplig kompetens
Hans Glaumann, vetenskaplig sekreterare *(infektionssjukdomar)*
Ulf Adamson *(medicin, endokrinologi)*, deltar ej i ärendena 2010/1071, 2010/1092, 2010/1124-1127, 2010/1129-1131, 2010/1133, 2010/1147, 2010/1153 och 2010/1168
Björn Almé *(psykiatri)*
Maria Ankarcrona *(geriatrik)*, deltar i ärendena 2010/1092, 2010/1124-1127, 2010/1129-1131, 2010/1133, 2010/1147, 2010/1153 och 2010/1168
Maria Feychting *(miljömedicin, epidemiologi)*
Niels Lynöe *(socialmedicin, omvårdnad)*
Gianni Celsi *(barnmedicin)*, deltar i ärendena 2010/998, 2010/1062 och 2010/1077-1078
Mårten Rosenqvist *(kardiologi)*
Björn Wrangsjö *(barn- och ungdomspsykiatri)*

Ledamöter som företräder allmänna intressen
Birgitta Mörk
Bengt Gustafsson
Gösta Johansson
Ulf Uebel
Lars Åstrand

Övriga närvarande
Anne Manninen, administrativ sekreterare
Stefan Borg, ersättare för Hans Glaumann

---

§ 1 Ordföranden förklarar sammanträdet öppnat.

§ 2 Ledamoten Mårten Rosenkvist ifrågasätter om inte s.k. kvalitetssäkringsstudier eller studier utan egentliga etiska problem skulle kunna hanteras av etikprövningsnämnden med ett enklare förfarande, t.ex. att dessa delegeras till ordföranden att fatta beslut efter föredragning av den vetenskapliga sekreteraren. Ordföranden lovade att föra ärendet vidare till övriga ordföranden i samband med ett nämndledningsmöte.

§ 3 Den administrativa sekreteraren anmäler att den vetenskaplige sekreteraren sedan föregående möte den 9 juni 2010 fattat beslut i 34 ärenden som avser ändring av godkännande.

ANSÖKAN OM ETIKPRÖVNING

Information till ansökan, ***se bilaga och Vägledningar (www.epn.se)***

Till Regionala etikprövningsnämnden i: Stockholm

Den regionala etikprövningsnämnd till vars upptagningsområde forskningshuvudmannen hör, se respektive nämnd *(www.epn.se)*

Avgift inbetald datum: 100614

Observera att en ansökan aldrig är komplett och därmed kan behandlas förrän blanketten är korrekt ifylld och avgiften är betald.

Projekttitel: Effekten av fysisk träning på sjukdomsmekanismer och symtom vid fibromyalgi .

Ange en beskrivande titel på svenska för lekmän, utan sekretesskyddad information. Ange också i förekommande fall projektets identitet, projektets/forskningsplanens (protokollets eller prövningsplanens) nummer, version, datum osv.

Projektnummer/identitet: Version nummer:

Uppgifter som fylls i av den regionala etikprövningsnämnden

Ansökan komplett: Dnr:

Begäran om ytterligare information (i sak): Begärd information inkommen:

Beslutsdatum: Expeditionsdatum:

Ansökan avser (gäller även vid begäran om rådgivande yttrande):

Forskning där endast en forskningshuvudman deltar (5 000 kr) ☐

Forskning där mer än en huvudman deltar (16 000 kr) ☒

Forskning där mer än en forskningshuvudman deltar, men där samtliga forskningspersoner eller forskningsobjekt har ett omedelbart samband med endast en av forskningshuvudmännen (5 000 kr) ☐

Endast behandling av personuppgifter (5 000 kr) ☐

Forskning som gäller klinisk läkemedelsprövning (16 000 kr) ☐

Ändring av tidigare godkänd ansökan enligt 4 § förordning (2003:615) om etikprövning av forskning som avser människor (2 000 kr) ☐

Om nämnden finner att forskningsprojektet inte faller inom etikprövninglagens tillämpningsområde önskas ett rådgivande yttrande. (Info: 4a och 4b §§ i förordning 2003:615) (Info: Bilaga till ansökan)

Ja: ☒ Nej: ☐

1. Information om forskningshuvudman m.m.

1:1 Forskningshuvudman (Info: p. 1:1 i Vägledning till ansökan)

Ansökan om etikprövning av forskning ska göras av forskningshuvudmannen. *Med forskningshuvudman avses en statlig myndighet eller en fysisk eller juridisk person i vars verksamhet forskningen utförs.* Inom staten utförs forskning främst vid lärosätena, men även vid vissa andra myndigheter, som t.ex. Brottsförebyggande rådet och Socialstyrelsen. Kommuner och landsting kan vara forskningshuvudmän, liksom privaträttsliga juridiska personer.

Namn: Institutionen för Klinisk Neurovetenskap, Karolinska Institutet
Adress: 171 77 Stockholm

1:2 Behörig företrädare för forskningshuvudmannen

Behörig företrädare är t.ex. prefekt, enhetschef, verksamhetschef. Forskningshuvudmännen bestämmer själva, genom interna arbets- och delegationsordningar eller genom fullmakt, vem som är behörig att företräda forskningshuvudmannen. Kopia av sådan handling *ska* bifogas.

Namn: Jan Hillert Tjänstetitel: Prefekt Institutionen för Klinisk Neurovetenskap
Adress: Institutionen för Klinisk Neurovetenskap, Karolinska Institutet, 171 77 Stockholm

1:3 Forskare som är huvudansvarig för genomförandet av projektet (kontaktperson)

(Info: p. 1:3 i Vägledning till ansökan)

Namn: Eva Kosek Tjänstetitel: Klinisk Lektor, Doc, Överläk
Adress: Retzius väg 8, Karolinska Institutet, 171 77 Stockholm
E-postadress: eva.kosek@ki.se
Telefon: 08-524 87054
Mobiltelefon: 0730-407755

1:4 Plats (Info: p. 1:4 i Vägledning till ansökan)

Plats (er) där projektet ska genomföras, ange inrättning (ar), institution (er), klinik (er) etc.

Göteborg:

Sjukgymnastik och Arbetsterapi, Sahlgrenska Universitetssjukhuset

Forsknings- och utvecklingsenheten, Reumatologi, Sahlgrenska Universitetssjukhuset

NärRehab Mitten Älvsborg, Alingsås

Linköping:

Smärt och Rehabcentrum, Linköpings Universitet, Linköping.

Stockholm:

Rehabiliteringsmedicinska Universitetskliniken, Danderyds Sjukhus, Danderyd.

Institutionen för Odontologi, Karolinska Institutet, Huddinge.

Institutionen för Klinisk Neurovetenskap, Karolinska Institutet, Solna.

1:5 Andra medverkande

Övriga deltagande forskningshuvudmän samt forskare ansvariga för att lokalt genomföra projektet (kontaktpersoner) anges här eller i bilaga med namn och adresser (se p. 9 bilaga nr 1).

Forskningshuvudmän:

Sjukgymnastik och Arbetsterapi, Sahlgrenska Universitetssjukhuset

Kontaktperson: Doc. Kaisa Mannerkorpi, Sjukgymnastiken SU/Sahlgrenska, Blå Stråket 3, 41345 Göteborg

Övriga medverkande:

Med Dr, reumatolog Jan Bjersing, Reumatologi, Sahlgrenska Universitetssjukhuset, Gröna Stråket 14, 41345 Göteborg

Leg sjukgymnast Anette Larsson, NärRehab Mitten Älvsborg, Herrljunga Vårdcentral, Distriktssjukgymnastiken, 524 32 Herrljunga

Linköpings Universitetssjukhus

Kontaktperson: Prof., Överläkare Björn Gerdle, Smärt och Rehabcentrum, Universitetssjukhuset, 581 85 Linköping.

Övriga medverkande: Överläkare, docent lektor Britt Larsson, Med Dr Kemist Bijar Ghafouri, ST-läkare doktorand Nazdar Ghafouri, Civiling Med Dr Anna Sjörs, ST-läkare Frida Forsberg, leg sjukgymnast Linn Karlsson alla på Smärt och Rehabcentrum, Universitetssjukhuset, 581 85 Linköping. .

Karolinska Institutet, Huddinge.

Kontaktperson: Doc., Övertandläkare Malin Ernberg, Institutionen för Odontologi, Box 4064, 141 04 Karolinska Universitetssjukhuset, Huddinge.

Övriga medverkande Stockholm:

Med.Dr. Leg sjukgymnast Monika Löfgren, Rehabilitetingsmedicinska Universitetskliniken, Danderyds Sjukhus AB, 182 88 Stockholm.

Med. Dr. Leg. läk Indre Ljungar, Rehabilitetingsmedicinska Universitetskliniken, Danderyds Sjukhus AB, 182 88 Stockholm.

Leg. sjukgymnast Philip Njoo, Rehabilitetingsmedicinska Universitetskliniken, Danderyds Sjukhus AB, 182 88 Stockholm.

Leg. psykolog, Med.Dr. Jens Gisselgård, Institutionen för Klinisk Neurovetenskap, Retzius väg 8, Karolinska Institutet, 171 77 Stockholm.

Nikolaus Christidis, Institutionen för Odontologi, Box 4064, 141 04 Karolinska Universitetssjukhuset, Huddinge.

Prof. Leg.läk. Martin Ingvar, Institutionen för Klinisk Neurovetenskap, Retzius väg 8, Karolinska Institutet, 171 77 Stockholm.

1:6 Ansökan/anmälan till andra myndigheter

Vid läkemedelsprövning

Ansökan om tillstånd av *Läkemedelsverket* – se Läkemedelsverkets hemsida (www.mpa.se)

Ansökan inlämnad (datum)

Tillstånd erhållits ☐

EudraCT nr:

Vid viss genetisk forskning

Om personuppgifter om genetiska anlag som har framkommit efter genetisk undersökning kommer att hanteras i studien ska detta anmälas till *Datainspektionen* enligt 10 § personuppgiftsförordningen (1998:1191) – se Datainspektionens hemsida www.datainspektionen.se

Anmälan inlämnad (datum)

Kommer att inlämnas efter godkänd etikprövning ☒

Vid viss forskning som innefattar bestrålning av forskningspersoner (Info: p. 9 i Vägledning till ansökan)

Ansökan, enligt 16 och 22 §§ Strålsäkerhetsmyndighetens föreskrifter (SSMFS 2008:35) om allmänna skyldigheter vid medicinsk och odontologisk verksamhet med joniserande strålning, till *Strålskyddskommitté* – för vidare information kontakta aktuell lokal strålskyddskommitté.

Ansökan inlämnad (datum): Ansökan tillstyrkt ☐

2. Uppgifter om projektet

2:1 Sammanfattande beskrivning av forskningsprojektet (programmet)

Vägledning till forskningsplan/forskningsprotokoll (program) (Info: p. 9 i Vägledning till ansökan)

Beskrivningen ska kunna förstås av nämndens samtliga ledamöter. Undvik därför terminologi som kräver specialkunskaper. Ange bakgrund och syfte för studien samt den/de vetenskapliga frågeställning (ar) som man söker svar på. Ange de viktigaste undersökningsvariablerna. Beskriv vilka kunskapsvinster projektet kan förväntas ge och betydelsen av dessa. Ange om det är en registerstudie, uppdragsforskning etc. För fackmän avsedd detaljerad information i forskningsplan/forskningsprotokoll (program) ska bifogas som bilaga (se p. 9 bilaga nr 2). En utförligare beskrivning av studiens genomförande *avsedd för lekmän* kan vid behov bifogas den för fackmän avsedda obligatoriska forskningsplanen.

Fibromyalgi (FM) är ett kroniskt smärtsyndrom kännetecknat av utbredd muskelsmärta, ömhet, trötthet och störd sömn. Prevalensen i befolkningnen är ca 2%, varav 80% kvinnor. Bland muskuloskelettala besvär är FM idag den dominerande orsaken till långvarig sjukfrånvaro hos kvinnor. Sjukvårdskostnaderna för denna grupp uppgick i Sverige till 865 miljoner kr/år medan de indirekta kostnadrena (sjukskrivning etc) uppgick till 12,5 miljarder kr/år (CMT Rapport 2003:5). WHO har erkänt FM som egen diagnos (ICD M79.7), men syndromet är fortfarande kontroversiellt fr.a. pga bristande förståelse för patogenesen. Sjukdomsmekanismerna vid FM är multifaktoriella. Detta avspeglas i behandlingen där teambaserat, multiprofessionellt omhändertagande med inslag av sjukgymnastik (fr.a. träning och avspänning), kognitiv beteendeterapi och läkemedelsbehandling dominerar (tre läkemedel har f.n. indikationen FM i USA, men inget i Europa). FM är således ett folkhälsoproblem som fr.a. drabbar kvinnor, leder till sänkt livskvalitet och arbetsoförmåga och där sjukdomsorsakerna är multifaktoriella och ofullständigt kända varför effektiv behandling saknas.

Tidigare forskning på området har identifierat såväl perifera som centrala störningar hos FM patienter. Perifert ses störd muskelgenomblödning med frisättning av smärtframkallande substanser. Centralt ses störd funktion av autonoma nervsystemet och bristande aktivering av kroppsegna smärthämmande system. De i detta nätverk ingående grupperna har var och en bidragit med internationellt uppmärksammad forskning inom sitt respektive område. Ett övergripande problem inom FM forskningen är att syndromets multifaktoriella karaktär ställer

stora krav på kompetens inom flera, traditionellt disparata forskningsområden och tillgång till avancerad och kostsam apparatur. Tyvärr har FM forskningen i Sverige, såväl som internationellt, präglats av att varje forskargrupp studerat FM utifrån sitt eget perspektiv med ringa samarbete mellan olika kompetensområden. Detta har medfört att vi å ena sidan har en relativt omfattande kunskap avseende olika smärtmekanismer vid FM, men å andra sidan nästan helt saknar förståelse för hur dessa mekanismer interagerar och hur olika behandlingar fungerar.

Träning utgör alltid en central komponent i behandlingen av patienter med FM. Det övergripande syftet med detta projekt är att studera effekten av träning på sjukdomsmekanismer vid FM, närmare bestämt undersöka hur perifera och centralnervösa mekanismer samverkar och hur denna samverkan kan påverkas av en strukturerad träningsintervention. Vi önskar även undersöka hur genetiska (ärftliga) faktorer inverkar på smärtkänslighet och effekt av träning. För att kunna besvara dessa frågeställningar är nätverkets första mål att genomföra en randomiserad, kontrollerad multicenterstudie avseende effekten av fysisk träning på patofysiologiska mekanismer, symtom, funktion och arbetsförmåga hos kvinnor med fibromyalgia (FM). Vi kommer att särskilt studera effekten av träning på autonoma nervsystemet, kroppsegna smärtreglerande system, inflammatoriska parametrar och muskelmetabolism. Effekterna relateras till förekomsten av kända genetiska faktorer (haplotyper) av betydelse för smärtkänslighet. De fyra nätverkscentra, som vardera representerar för projektet specifika kompetensområden samarbetar i denna studie. Totalt rekryteras 144 kvinnliga FM patienter och 144 friska kvinnliga kontroller (48 st vardera i Göteborg, Linköping och Stockholm). FM patienterna randomiseras till specifik styrketräning (n = 72) eller avspänningsträning (kontrollintervention, n = 72). Friska kontroller randomiseras till styrketräning (n = 36) eller passiva kontroller (n = 108). Styrke- resp. avspänningsträningsinterventionen genomförs 2 ggr/vecka i 15 veckor under handledning av speciellt utbildade sjukgymnster. Försökspersonerna genomgår en strukturerad intervju om tidigare och aktuella smärtor, andra sjukdomar samt konsekvenser för daglig livsföring (inkl sjukskrivning). Med validerade enkäter kartläggs smärtans utbredning, intensitet och konsekvenser för dagligt liv, depressiva symptom och ångest, katastroftänkande, rörelserädsla, hälsoupplevelse och arbetsförmåga före, direkt efter och 1 år efter avslutad träningsintervention. Ett batteri av funktionella test görs före och efter träningsinterventionen. Blodprov tas på alla försökspersoner för analys av substanser relevanta för smärtreglering och inflammation (cytokiner, nerve growth factor, endogena opioider, endocannabinoider, katekolaminer, neuropeptider och proteomic) före och efter avslutad intervention. Salivprov tas för genetisk kartläggning av gener relevanta för smärtreglering (genotypning). Smärtkänslighet i vila och funktionen av kroppsegna smärtreglerande system under standardiserat statiskt muskelarbete undersöks med tryckalgometri. Central smärtmodulering undersöks även genom registrering av smärtkänslighet för värme och kyla samt förändringar i smärtupplevelse under 2 minuters smärtsam värmestimulering av huden (Thermotest). Tryckalgometritesten och Thermotestet

utförs före och efter avslutad träningsintervention och relateras till den kliniska effekten av träning och de olika genotyperna. Hjärtfrekvensvariabilitet som ger en uppfattning om autonom funktion registreras under 24 timmar före resp. efter genomgången träningsintervention hos 72 FM patienter och 36 kontroller. Microdialys av en lårmuskel (m. quadriceps) genomförs i vila och efter standardiserat muskelarbete med hjälp av en metod som utarbetats av Linköpingsgruppen (Rosendal et al. Pain 2004;112:324-34). Dialysatet analyseras avseende relevanta muskelmetaboliter och substanser relevanta för smärtmodulering och inflammation (cytokiner, nerve growth factor, endogena opioider, endocannabinoider, catecholaminer, serotonin och neuropeptider). Microdialys utförs på 36 FM patienter och 36 friska kontroller före och efter styrketräningsinterventionen. I samband med microdialysen tas även en stansbiopsi från huden för analys av kroppsegna opioider och endocanabinoider, ämnen relevanta för kroppsegna smärthämmande system. 24 FM patienter och 12 kontroller undersöks med funktionell magnetisk resonsansimaging (fMRI) avseende effekten av träning på viloaktivitet och smärtbetingad hjärnaktivering, vilket avspeglar hur kroppsegna smärtreglerande system aktiveras i hjärnan före och efter träningsinterventionen.

Frågeställningarna är höggradigt relevanta och kan endast besvaras i en studie där försökspersonerna kan karaktäriseras avseende multipla variabler, vilket kräver ett intimt samarbete mellan olika forskargrupper. En fördjupad kunskap om muskulära och centralnervösa faktorers roll vid FM ökar möjligheterna att utveckla effektiv behandling. Kännedom om ev. genetiska faktorers betydelse skulle öka möjligheterna för att identifiera individer med ökad risk att utveckla FM och därmed möjliggöra prevention. Genusaspekter är höggradigt relevanta i projektet då enbart kvinnor studeras utifrån att FM är väsentligt vanligare bland kvinnor. Vid kroniska smärtillstånd är fysikalisk behandling, som en del i multimodal rehabilitering, en adekvat åtgärd enligt SBU rapport av år 2006 (Gerdle & Gullacksen, SBU 2006;159-213). Integrerad kunskap om intramuskulära mekanismerna, centralnervösa mekanismer och betydelsen av genetiska faktrorer vid smärtlindrande muskelträning ger nya förutsättningar att förbättra och vetenskapligt förankra sådana fysikaliska behandlingskomponenter.

Projektet har således förutsättningar att innebära positiva effekter för de drabbade individerna, för arbetsliv och för samhällsekonomi.

2:2 Vilken/vilka vetenskaplig (a) frågeställning (ar) ligger till grund för projektets utformning?

Om projektet kan karakteriseras som en hypotesprövning, ange den primära och eventuellt sekundära hypotesen. Hänvisning till mer detaljerad information för fackmän kan ske till bifogad forskningsplan enligt punkt 2:1

Nätverket har identifierat några angelägna forskningshypoteser som kräver en samverkan mellan forskargrupperna för att kunna studeras:

1) FM smärtan är relaterad till en störning i autonoma nervsystemet (ökad sympatikustonus), vilket ger upphov till metaboliska förändringar i muskulaturen, dvs en obalans mellan pro- och anti-inflammatoriska cytokiner och mellan smärthämmande (t.ex. opioider, endocannabinoider) och smärtframkallande (nerve growth factor, peptider) substanser i blod och i muskulatur.

2) Den ökade smärtkänsligheten och den bristande funktionen av kroppsegna smärthämmande system hos FM patienter påverkas av genetiska faktorer av betydelse för reglering av metabolismen av transmittorsubstanser involverade i smärtreglerande system (t.ex. opioider).

3) Fysisk träning normaliserar funktionen av autonoma nervsystemet och medför minskade koncentrationer av pro-inflammatoriska och smärtframkallande substanser i blod och muskulatur och därmed minskad smärta.

4) Vissa genotyper svarar bättre på fysisk träning på grund av en ärftligt betingad bättre funktion av kroppsegna opioidberoende smärthämmande system. Detta kan studeras genom att undersöka koncentrationerna av kroppsegna opioider och endocannabinoider och deras resp. receptorer före och efter en träningsintervention i relation till funktionen av kroppsegna smärthämmande system hos de olika genotyperna.

5) Fysisk träning minskar symtom och aktivitetsbegränsningar hos patienter med FM vilket leder till förbättrad funktion, livskvalitet och arbetsförmåga.

2:3 Redogör för resultat från relevanta djurförsök

Om djurförsök inte utförts ange skälen till detta.

Studien kräver initierad medverkan av försökspersoner (t.ex. att svara på frågeformulär) och en verbal kommunikation mellan undersökare och försöksperson vilket inte låter sig göras på djur. Smärta är en subjektiv upplevelse och djur saknar förmåga att skatta smärta. Dessutom saknas djurmodeller för FM.

2:4 Redogör översiktligt för undersökningsprocedur, datainsamling och datas karaktär

(Info: p. 2:4 i Vägledning till ansökan)

Av beskrivningen ska framgå hur projektet planeras genomföras. Beskriv insamlade datas karaktär. Ange hur datas tillförlitlighet säkerställs (t.ex. kvalitetskontroll/monitorering). - Vid enkäter och intervjuer ska beskrivas

tillvägagångssätt och t.ex. frågors innehåll och hur slutsatser dras. Enkäter och skattningsskalor _ska_ bifogas (se p. 9 bilaga nr 5). - För medicinsk forskning ska anges t.ex. typer av ingrepp, mätmetoder, antal besök, tidsåtgång vid varje försök, doser och administrationssätt för eventuella läkemedel och/eller isotoper, blodprovsmängd (även ackumulerad mängd vid multipla försök). Ange även om och på vilket sätt undersökningsprocedur m.m. skiljer sig från klinisk rutin. Ange proceduren för att ge den eventuella behandling efter projektets slut, som kan erfordras. Ange procedur för insamling av biologiskt material. Redogör för datakällor och procedurer vid behandling av personuppgifter. För mer detaljerad information kan hänvisning ske till bilagd forskningsplan.

Försökspersonerna (fp) i denna randomiserade kontrollerade multicenterstudie kommer att rekryteras genom annonsering i dagspressen. Tre centra deltar, Göteborg, Linköping och Stockholm. Varje centrer kommer att rekrytera 48 kvinnliga FM patienter och 48 friska åldersmatchade kvinnor. Försökspersoner som anmäler intresse kommer att kontaktas per telefon för att informeras muntligen om studien och skriftlig information skickas hem till de som visar intresse. Fp tillfrågas om de även kan tänka sig att delta i hjärtfrekvensvariabilitets analys (HRV) (FM n = 72; friska n = 36) och microdialys (FM n = 36; friska n = 36) och för Stockholmsdeltagarna genomgå en funktionell magnetisk resonansimaging undersökning (fMRI)(FM n = 24, kontroller n = 12). Friska försökspersoner intervjuas avseende ev. exklusionskriterier och de som samtycker till deltagande kallas direkt till besök 2.

Besök 1: Screeningbesök (alla FM patienter): Efter att skriftligt samtycke erhållits kallas fp till ett första besök (tidsåtgång ca 1 timme). Därvid kontrolleras att fp uppfyller inklusionskriterierna och att inga exklusionskriterier föreligger (se nedan). Hos FM patienter inhämtas uppgifter avseende smärtduration, när de erhållit diagnosen FM, ev andra genomgångna eller aktuella sjukdomar (somatiska/psykiska), alkoholkonsumtion undersöks med hjälp av frågeformuläret Audit, aktuell medicinering gås igenom. Alla FM pat undersöks av leg. läk för att fastställa diagnosen FM och för att utesluta annan sjuklighet som utgör kontraindikation för deltagande i studien. Slutligen fastställs vilka mediciner som fp ska göra uppehåll med 48 timmar före mätningarna (besök 2, 3 , 4 och 5) resp. 7 dagar före microdialys (besök 3 och 7).

Inklusionskriterier: Kvinnor 20-65 år som uppfyller ACR-1990 klassifikationskriterier för FM resp. friska kvinnor 20-65 år gamla.

Exklusionskriterier (båda grupperna):

Högt blodtryck (>160/90 mmHg) och/eller pågående farmakologisk behandling mot högt blodtryck.

Uttalad och smärtsam artros höft eller knä.

Höga skattningar på Audit (> 6), talande för hög alkoholkonsumtion.

Andra sjukdomar: Reumatiska inflammatoriska sjukdomar (t.ex. RA, SLE), neurologiska sjukdomar (t.ex. MS, ALS), sockersjuka (diabetes mellitus), hjärt-kärl sjukdomar, andra dominerande orsaker till smärta alt. smärtsamma tillstånd som omöjliggör träning, svårare psykiska sjukdomar (t.ex. depression/ångest), pågående alkohol eller narkotika överkonsumtion/missbruk .

Fp som senaste året genomgått rehabiliteringsprogram med strukturerad styrketräning resp. avspänningsträning. Fp som styrketränar alt. praktiserar avspänning regelbundet > 2 gånger i veckan.

Andra faktorer som enligt försöksledaren utgör hinder för deltagande i studien.

Bristande kunskaper i svenska som omöjliggör besvarande av enkäter.

Gällande fMRI delen av studien: Metallimplantat i kroppen, vänsterhänthet och klaustrofobi.

FM patienter som inte kan tänka sig att avstå från smärtstillande (analgetika), antiinflammatoriska läkemedel (NSAID) eller sömnmedel 48 timmar före mätningarna (blodprov, fMRI).

FM patienter som inte kan tänka sig att avstå från antiinflammatoriska läkemedel (NSAID) 7 dagar före microdialys kan inte delta i microdialysdelen av studien.

Fp tillfrågas konsekutivt om de även kan tänka sig att delta i microdialysdelen av studien.

Vecka 1: Försökspersoner som efter genomgången skreening uppfyller kriterierna för att delta i studien och som vill delta kallas till besök 2.

Besök 2 basmätningar (alla)(tidsåtgång ca 2 timmar; 2,5 timmar för friska försökspersoner): Friska försökspersoner tillfrågas om sin hälsa och får fylla i Audit (alkoholvanor). Alla försökspersonerna fyller i frågeformulär avseende smärtintensitet (visuell analog skala, VAS), smärtutbredning (smärtteckning), pain disability index (PDI), psykologiska parameterar; hospital anxiety and depression scale (HADS), pain catastrophizing scale (PCS), chronic pain acceptance questionnnaire (CPAQ) and fear-avoidance beliefs questionnaire (FABQ), frågeformulär rörande fysisk aktivitet och träningsvanor; exercise self-efficacy scale, experience of physical activity (EPA), perceived physical work demands (enl. Balogh) and leisure time physical activity (LTPAI), sömn/trötthet; Pittsburgh sleep inventory index (PSIQ), multidimensional fatigue inventory (MFI) och livskvalitet; fibromyalgia impact questionnaire (FIQ) och short-form health survey (SF-36), se bifogade frågeformulär. Frågeformulären utvärderas enligt sedvanlig standard och monitorering sker för att säkra tillförlitligheten av data i samband med att de överförs till avidentifierade filer för statistisk analys.

Vikt och längd kontrolleras.

Smärttest: Smärttrösklar för tryck undersöks med en s.k tryckalgometer. Därvid trycker försöksledaren med en stav mot försökspersonen och försökspersonen signalerar när trycket övergår till förnimmbar smärta (i "minsta lilla smärta") varid tryckningen avbryts. Smärttrösklarna undersöks på 8 olika ställen på kroppen (båda sidor av kroppen över skuldermuskulatur, i armbågsnivå, över skinkorna och strax ovan knänledern) i vila. Dessutom undersöks smärttrösklar för tryck över lårmuskel och överarmsmuskel på icke-dominant sida i vila och under standardiserad statisk muskelkontraktion motsvarande ca 30% av individens maximala styrka (MVC) för att undersöka funktionen av kroppsegna smärthämmande system. Den minskade känsligheten över arbetande muskel avspeglar aktivering av segmentellt och minskad känslighet över den vilande överarmsmuskeln avspeglar funktionen av ett generellt kroppseget smärthämmande system. Slutligen undersöks känseln för värme och kyla. Därvid läggs en metallplatta (thermod) mot försökspersonenes hud på låret (icke-dominat sida) och temperaturen i denna regleras så att den blir varm resp. kall. Försökspersonen ombeds att trycka på en knapp när hon känner minsta lilla värme eller kyla resp. när värmen/kylan gör minsta lilla ont, varvid stimuleringen omedelbart avbryts. Därefter ber man försökspersonen trycka på knappen först när värmen motsvarar en smärta som skulle skattas som 4 av 10 resp. 7 av 10 på Borgs 11-gradiga intensitetsskala. Medelvärdet av varje individs temperaturen vid 4/10 och 7/10 beräknas. Försökspersonerna stimuleras sedan i 2 min med denna temperatur och får skatta smärtintensiteten på VAS i början av stimuleringen och efter 30, 60, 90 och 120 sekunder. Eventuell förändring av smärtintensiteten avspeglar om kroppen vänjer sig (habituering) eller om smärtan förstärks (sensitisering).

Styrkemätningar och funktionella test: 6 minuters gångtest utförs varvid man mäter hur lång sträcka personen kan gå på 6 min. Maximal styrka i lårmuskel, armmuskel och handgrepp mäts med dynamometer. Försökspersonerna ombeds då att ta i allt vad de orkar under 15 sek 3 gånger för varje test och det bästa värdet räknas. Slutligen utförs ett stående funktionellt balanstest.

Blodprov: ca 20 ml tas för analys av rutinprover, endogena opioider och endokannabinoider, cytokiner och andra pro-inflammatoriska substanser, transmittorsubstanser (t.ex. NGF, SP), metaboliter (t.ex. laktat) samt analys av proteinbilden (proteomic). (Utom patienter som ska genomgå microdialys, där blodprovet tas i samband med besök 3). Patienter i Göteborg undersöks på sjukgymnastavdelning och får därför uppsöka sjukhus Reumatologiska kliniken, Sahlgrenska Universitetssjukhuset, Göteborg eller Alingsås lasarett för blodprovstagning.

Salivprov tas för genetisk analys av gener relevanta för smärtreglering (t.ex. COMT, SERT).

Hjärtvariabilitetsanalys (HRV)(gäller endast 72 FM patienter och 36 friska): EKG-elektroder sätts fast på 6 ställen på försökspersonens bröstkorg och kopplas till en liten portabel EKG-bandspelare (Holter). Försökspersonerna uppmanas att leva som vanigt med bandsplearen på

under 24 timmar (med undantag för dusch/bad). Efter 24 timmar får försökspersonerna ta av elektroderna och lämna tillbaka bandspelaren.

Försökspersoner som inte ska genomgå microdialys randomiseras till styrke resp. avspänningsträning.

Besök 3; microdialys (36 FM patienter och 36 friska) (tidsåtgång ca 6 timmar). Efter information och demonstration av apparatur bedövas huden i en punkt över den icke-dominanta lårmuskeln (quadriceps; vastus lateralis) med lokalt bedövningsmedel (subkutan anestesi 0.2–0.5 ml Xylocain (20 mg/ml) utan adrenalin). Två microdialyskatetrar förs in med 2 cm mellanrum i muskeln med 45 graders vinkel längst muskefibrerna. Proberna kopplas till mikrodialyspump med sedvanliga, icke-vävnadsretande perfusionslösningar och prover (100 μL) samlas var 20:e minut. 120 min efter start av mikrodialys ombeds försökspersonenerna att spänna lårmuskeln vid upprepade tillfällen (standardiserad knästräckning mot motstånd i form av intermittenta statiska kontraktioner) under 20 min varefter mikrodialysen fortsätter i 120 min. Total tid för mikrodialys motsvarar 4 timmar 20 min. Smärtskattningar (VAS) registreras var 60:e min innan och efter statiskt arbete samt var 5:e minut under statiskt arbete (dvs vid tiderna 60, 120, 125, 130, 135, 140, 200 och 260 min). Efter avslutad mikrodialys bedövas huden över låret igen enl. ovan och en stanshudbiopsi i huden tas (4-6 mm i diameter) innan dialyskatetrarna dras ut. Försökspersoner som genomgått microdialys randomiseras därefter till styrke- eller avspänningsträning.

Besök 4; kalibrering inför fMRI undersökning 24 FM patienter och 12 kontroller (tidsåtgång ca 1 timme). Smärttröskel och smärtkänslighet utvärderas med hjälp av automatiserat tryck mot tumnageln, som i forskningen anses ge en representativ bild av en persons smärtrespons (Petzke et al. J. Rheumatol. 2003;30:567-574). Försöksledaren börjar med att ge mycket lätt, knappt kännbart tryck för att sedan öka trycket tills deltagaren anger att trycket på tumnageln övergår från "att kännas" till "att göra ont" vilket representerar deltagarens individuella smärttröskel. Dessa stimuleringar utförs med hjälp av en automatiserad stimulator där tumnagelsbädden stimuleras i 2,5 sekunder med hjälp av en automatiserad, luftdriven, datoriserad tryckstimulator med en gummipropp mot tumnageln. Efter varje stimulering kommer deltagaren att ange sin upplevda smärtnivå med hjälp av en visuell analog skala (VAS). Mellan varje stimulering kommer det att gå minst 30 sekunder för att undvika kvardröjande smärta från stimuleringen innan vilket kan leda till ökad känslighet. Efter att smärttröskeln är fastställd kommer ytterligare stimuleringar med ökat tryck att ges tills deltagaren anger att smärtan motsvarar 60 mm av 100 mm på VAS-skalan. Detta tryck utgör deltagarens individuella maxnivå i denna studie. Utöver den första stigande tryckomgången där deltagarens smärttröskel, samt 60 mm på VAS-skalan fastställs kommer en randomiserad tryckstimulering med 15 tryck att utföras där deltagaren också skattar sin upplevda smärtnivå för varje stimulering på VAS-skalan. Detta görs för att kalibrera

patientens smärtskattningar. På detta sätt kan inte deltagaren förutse huruvida ett lättare eller hårdare tryck kommer att ges vilket är en väldigt viktig förutsättning för att få bort så mycket som möjligt av psykologiska effekter i smärtresponsen och rapporteringen på VAS-skalan (Petzke et al. Pain 2003;105:403-413).

Besök 5; fMRI-undersökning 24 FM patienter och 12 friska kontroller (tidsåtgång ca 1 timme).

fMRI undersökning i vila och under smärtprovokation i form av tryck på tumnageln enl. ovan.

Vecka 2-16.

Försökspersonerna tränar under handledning av leg. sjukgymnast. De första träningsgångerna tar ca 2 timmar eftersom träningsregimen då gås igenom. Därefter tar passen ca 1 timme.

Styrketräning: I denna studie har vi valt ett beprövat koncept vid träning av människor med FM nämligen träning som påbörjas med låga belastningar dvs 40% av individuell maximal muskelspänning (MVC) och successivt ökas till 70-80% MVC (Valkeinen et al. Arthritis & Rheum 2006;54:1333-39). Träningsintensiteten individualiseras för varje deltagare utifrån styrkemätningar och följs upp kontinuerligt.

Träningen leds av speciellt utbildade leg. sjukgymnaster som motiverar och uppmuntrar försökspersonerna och vägleder dem i att utföra träningen på ett korrekt och för kroppen skonsamt sätt. Varje försöksperson dokumenterar sin träning i en träningsdagbok. Ökning av belastningar beslutas i samråd mellan tränande sjukgymnast och försöksperson. Träningspassen utförs i grupper av 5 FM patienter vilket ger möjlighet till socialt stöd och uppmuntran. Vid smärtökning minskas belastningarna tillfälligt. Ev. avvikelser från protokollet diskuteras med träningskoordinatorn på varje center och dokumenteras.

Träningsprotokoll:

10 min uppvärming på ergometercykel, egenvald belastning 40 - 75 Watt.

Styrkeövningar av knästräckare, knäböjare, höftsträckare, magmuskulatur, ryggmuskulatur, armbågsböjare, fotledssträckare och handgreppsstyrka. Träning av bålstabilitet integreras i alla övningar. Varje träningspass avslutas med ett stretching program.

Avspänningsträning: I denna studie har vi valt en beprövad metodik för avspänning som kontrollintervention. Sessionerna kommer att starta med ett standardiserat avspänningsprogram i liggande (Rudberg Natur & Kultur 1985), följt av ett stretchingprogram i syfte att reducera muskespänning. Avspänningsträningen sker i grupper om 10. Försökspersonernas upplevelse av avspänningen diskuteras och ev. modifieringar görs i samråd med sjukgymnasten i likhet med styrketräningsinterventionerna.

Vecka 17: Besök 6 och 7 som är identiska med besök 2 resp. 3 förutom att försökspersonerna även fyller i en fråga om de tycker att de förbättrats (patient global impression of change, PGIC) vid besök 6. De 24 FM patienter och 12 friska kontroller som deltog i fMRI undersökningen (besök 4 och 5) gör om samma undersökning i samband med besök 8 och 9.

Frågeformulären skickas hem till alla försökspersoner som genomgått tränings/avspänningsinterventionen (FM patienter n = 144, friska n =36) 1 år efter avslutad träning och returneras i frankerade kuvert.

2:5 Redogör för om insamlat biologiskt material kommer att förvaras i en biobank

(Info: p. 2:5 i Vägledning till ansökan)

Med biobank avses biologiskt material från en eller flera människor som samlas och bevaras tills vidare eller för en bestämd tid och vars ursprung kan härledas till den eller de människor från vilka materialet härrör. Redogör för var och hur prover som ska sparas förvaras, kodningsprocedurer och villkor för utlämnande av prover. Ange huvudman för biobanken. Observera att i förekommande fall ska anmälan av biobank ske till Socialstyrelsen enligt lagen (2002:297) om biobanker i hälso- och sjukvården m.m.

Samtliga blodprover märks endast med varje deltagares kodnummer och förflyttas efter insamling till Karolinska Institutets biobank, Sahlgrenska sjukhusets biobank respektive Linköpings Universitetssjukhus biobank. Biobankerna ser till att hanteringen av det insamlade biologiska materialet hanteras i enlighet med gällande lagstiftning. Salivprover för genotypning förvaras samtliga på Karolinska Institutets biobank.

De biologiska proverna lagras i frysar och flytande nitrogentankar. Alla prover och processer är möjliga att spåra då man använder sig av streckkodsmärkning och ett automatiserat sätt att hantera prover. Man har mycket höga säkerhetskrav för att undvika att prover blir förstörda eller ihopblandade, eller att icke-auktoriserade personer får tillgång till materialet. Enligt den svenska biobankslagen (2002:297) är alla prover som lagras i biobanken registrerade och märkta på ett sådant sätt att det tillåter att prover kan spåras till en donator och ett personnummer. Personuppgiftsansvarig är respektive universitetssjukhus.

Säkerhet och sekretess gällande de prover som registereras i biobanken säkras av datasystem med mycket hög säkerhet. Backup för databaserna, begränsad tillgång till datorerna och loggar för all inläggning av data och ändringar är några av de rutiner som tillämpas. Registreringen av personuppgifter och överföring av sådana uppgifter regleras av lagstiftning. Enligt denna lagstiftning krävs det att samtliga försökspersoner ger sitt samtycke till registrering i biobanken och dessutom kommer försökspersonerna att informeras om att de har rätt att få reda på vad som

finns registrerat samt att de kan begära att ta bort sin personliga information ur biobanken (se bilaga).

2:6 Redovisa tillgång till nödvändiga resurser under projektets genomförande

Ange vem/vilka som har ansvaret (prefekt, verksamhetschef eller motsvarande) för forskningspersonernas säkerhet vid alla enheter/kliniker där forskningspersoner ska delta. Intyg från dessa ansvariga *ska* bifogas (se p. 9 bilaga nr 9). Av intyget ska framgå att erforderliga ekonomiska, strukturella och personella resurser finns tillgängliga för att garantera forskningspersonernas säkerhet.

Göteborg:

Verksamhetschef, Boel Mörck, Reumatologi, Sahlgrenska Universitetssjukhuset, Gröna Stråket 14, 413 46 Göteborg.

Verksamhetschef, Eva Beckung, Sjukgymnastik och Arbetsterapi, Sahlgrenska Universitetssjukhuset, Hus A4, 431 80 Mölndal.

Verksamhetschef, Helen Andersson, När Rehab Alingsås, Alingsås Lasarett, 441 83 Alingsås.

Linköping:

Verksamhetschef, professor Björn Gerdle, Smärt- och rehabiliteringscentrum, Universitetssjukhuset, 581 85 Linköping.

Stockholm;

Prefekt Institutionen för Kliniks Neurovetenskap Prof. Överläk. Jan Hillert, Karolinska Institutet, Solna.

Klinikchef Mikael Ryd, Rehabiliteringsmedicinska Universitetskliniken, Danderyd Sjukhus AB, Stockholm.

Prefekt Institutionen för Dept. of Dental Medicine Prof. Kaj Fried, Alfred Nobels Allé 8, Huddinge.

2:7 Journalföring, registrering och hantering av data (Info: p. 2:7 i Vägledning till ansökan)

Redogör för hur undersökningsprocedurer och eventuella ingrepp journalförs. Ange hur registrering och behandling av resultaten ska gå till. Om materialet ska kodas, ange proceduren, vem som förvarar kodlistor och

vem eller vilka som har tillgång till dem, var och hur länge de förvaras samt om materialet kommer att anonymiseras eller förstöras. Ange om band- och videoinspelningar används. Redogör för vilken tillgänglighet datamaterialet har och hur det förvaras samt hur erforderligt sekretesskydd erhålls.

Varje försöksperson registreras i en individuell försöksmapp. All publicering sker på sådant sätt att en enskild försökspersons resultat ej kan identifieras. All datahantering sker med avidentifierade data. Endast en pappersnyckel/center bevaras i låst journalskåp. Deltagarnas mätningsresultat kommer att förvaras identitetsmärkta i separata pärmar i låsta skåp. För patienter gäller sedvanlig sekretess inom sjukvården. Tillgång till datamaterialet har projektledare, forskare som arbetar i projektet och personal inom de aktuella vårdenheterna med arbetsuppgifter i projektet.

Materialet sparas i 10 år.

2:8 Redogör för tidigare erfarenheter (egna och/eller andras) av den använda proceduren, tekniken eller behandlingen

Särskilt angeläget är att redovisning av risker för komplikationer görs tydliga och i förekommande fall med angivande av relevanta publikationer. Vid nya behandlingar av patienter, t.ex. med läkemedel, bör anges hur många patienter (med aktuell eller annan åkomma) som tidigare erhållit föreslagen behandling, läkemedelsdosering (eller annan dosering) samt hur långa behandlingsperioder som studerats.

Docent, Leg. sjukgymnast Kaisa Mannerkorpi har specialiserat sig på att studera effekterna av träning på FM och har lång erfarenhet av att träna patienter med FM. I detta projekt ansvarar Doc Mannerkorpi för träningsupplägget på samtliga centra och för att inskola de leg. sjukgymnaster som kommer att genomföra träningsinterventionerna på resp. center. Med. Dr. Leg. sjukgymnast Monika Löfgrens specialitet är att utveckla och studera effekterna av basal kroppskännedomsträning/avspänning hos patienter med FM. Dr. Löfgren ansvarar för upplägget av avspänningsinterventionen för samtliga centra och för att inskola de leg. sjukgymnaster som kommer att utföra avspänningsinterventionen på resp. center. Såväl styrke som avspänningsträning rekommenderas som behandling vid FM och ingår rutinmässigt i det kliniska omhändertagandet av patienterna.

Beträffande smärtmätningarna och fMRI undersökningarna har Docent Eva Kosek använt de aktuella metoderna i tidigare studier. Tryckalgometri och Thermotest används även i diagnostiskt syfte i klinisk rutinsjukvård t.ex. för diagnostik av neuropatiska smärttillstånd. Oss veterligen har inga allvarligare komplikationer tillstött.

Såväl Docent Kosek som Prof. Gerdle och Med Dr civiling Anna Sjörs har erfarenhet av hjärtfrekvensvariabilitetsanalyser på smärtpatienter. Goda kunskaper om analys av data finns

inom forskningsgruppen i Linköping som kommer att ansvara för analys av data från samtliga centra i denna studie.

Microdialys i muskel är en metodik som såväl Docent Malin Ernberg som Prof. Björn Gerdle är väl förtrogna med. Båda har ett flertal publikationer där metodiken använts. Inga allvarligare komplikationer (tex infektioner) har förelegat.

De seniora forskarna på samtliga centra är väl förtrogna med de aktuella frågeformulären som ofta använts i klinisk praxis och i forskning. Blodprovstagningen följer sedvanliga rutiner som är standard inom klinisk praxis.

3. Uppgifter om forskningspersoner

3:1 Hur görs urvalet av forskningspersoner? (Info: p. 3:1 i Vägledning till ansökan)

Med forskningsperson avses en levande människa som forskningen avser. Ange urvalskriterier (inklusion och exklusion). Redogör för på vilket sätt forskaren kommer i kontakt med/får kännedom om lämpliga forskningspersoner. Ange om rekrytering sker från egna/andras tidigare eller pågående studier. Om annonsering sker, *ska* annonsmaterialet insändas som bilaga (se p. 9 bilaga nr 3). Om t.ex. barn eller personer som tillfälligt eller permanent inte är kapabla att ge ett eget informerat samtycke ska ingå i projektet, ska detta särskilt motiveras. Om vissa grupper (t.ex. kvinnor, barn eller äldre) utesluts från deltagande i projektet ska detta särskilt motiveras.

De tre centra annonserar efter forskningspersoner i dagspress, v.g. se annonsmaterialtet som bilaga. Eftersom FM till 90% drabbar kvinnor kommer män att uteslutas ur denna studie för att minska variabiliteten.

Beträffande inklusions och exklusionskriterier se under 2.4 i denna ansökan.

3:2 Ange relationen mellan forskare/försöksledare och forskningspersonerna

- ☐ Behandlare (t.ex. läkare, psykolog, sjukgymnast) - forskningsperson (t.ex. patient, klient)
- ☐ Kursgivare (lärare) - student
- ☐ Arbetsgivare - anställd
- ☒ Annan relation som kan tänkas medföra risk för påverkan. Beskriv:

Alla mätningar sker av legitimerad sjukvårdspersonal dock ej patientens ordinarie vårdgivare. De sjukgymnaster som deltar i träning resp. avspänningsinterventionerna deltar inte i utvärderingen.

3:3 Redogör för det statistiska underlaget för studiepopulationens (-ernas)/ undersökningsmaterialets (-ens) storlek (Info: p. 3:3 i Vägledning till ansökan)

Redovisa statistisk styrka, så kallad "power"- beräkning eller redovisa motsvarande överväganden som tydliggör studiens möjligheter att besvara frågeställningarna.

Doc Ernberg och Prof. Gerdle har i tidigare microdialysstudier inkluderat 15-20 patienter/grupp och kunnat detektera gruppskillnader. Eftersom vi i denna studie vill kunna påvisa även effekter av träning vill vi kunna ha power att detektera även något mindre skillnader varför 36 försökspersoner/grupp torde vara adekvat i samband med parad statistik.

Beträffande fMRI har vi tidigare med samma metodik som ska användas i denna studie kunnat påvisa skillnader mellan 16 FM patienter och 16 friska kontroller, varför 24 personer/grupp torde vara adekvat med hänsyn taget till troligt bortfall i träningsstudien. Vi har tillgång till kontrollmaterial från friska kvinnor.

Effekt av träningsintervention på smärthämmande system och förbättrad funktion/livskvalitet kräver större material då vi måste räkna med ett relativt stort bortfall av försökspersoner. Vi räknar med att det ska vara tillräckligt om minst 50 FM patienter/interventionsgrupp fullföljer studien med hög kompliance varför 72 försökspersoner/grupp torde vara adekvat.

De genotyper vi främst är intresserade av finns hos ca 25% av befolkningen. Vi behöver ca 15 försökspersoner/genotyp för att kunna undersöka sambandet mellan genotyp och smärtreglering. Således kommer materialet att räcka till baseline och borde vara tillräckligt för de huvudsakliga analyserna även efter träningsinterventionen förutsatt att bortfallet inte blir för stort.

3:4 Ange om forskningspersonerna kan komma att inkluderas i flera studier samtidigt eller i annan/andra studie (-er) i nära anslutning till denna? I så fall vilken typ av forskning? (Info: p. 3:4 i Vägledning till ansökan)

Nej

3:5 Vilket försäkringsskydd finns för de forskningspersoner som deltar i projektet?

Det åligger forskningshuvudmannen att kontrollera att befintliga försäkringar täcker eventuella skador som kan uppkomma.

Patienterna täcks av patientförsäkringen eftersom träningen och mätningarna sker av legitimerad sjukvårdspersonal med anställning inom landstinget. Om legitimerad sjukvårdspersonal som inte innehar anställning inom landsting anlitas kommer centrat i fråga att betala en tilläggsförsäkring för de aktuella försöksperonerna.

3:6 Vilken ekonomisk ersättning eller andra förmåner utgår till de forskningspersoner som deltar i projektet och när betalas ersättningen ut? Utförligare beskrivning kan lämnas i bilaga.
(Info: p. 3:6 i Vägledning till ansökan)

Ersättning för obehag och besvär. Belopp (före skatt): V.g se bilaga

Ersättning för förlorad arbetsinkomst ☐ Ja ☒ Nej

Reseersättning ☐ Ja ☒ Nej

Befrielse från kostnader för läkemedel ☐ Ja ☒ Nej

Befrielse från andra kostnader. Vilka? Nej

Andra förmåner. Vilka? ev. gymkort 6 månader

När betalas ersättningen ut? Ekonomisk ersättning efter sista mätningens genomförande.

Ingen ersättning betalas ut ☐

4. Information och samtycke (Info: Forskningspersonsinformation)

4:1 Proceduren för och innehållet i den *information* som lämnas då forskningspersoner tillfrågas om deltagande

Beskriv hur och när information ges och vad den innehåller. Ange vem som informerar. Normalt ska en kortfattad och lättförståelig skriftlig information ges. Denna skriftliga information *ska* bifogas ansökan (se p. 9 bilaga nr 4). Om ingen eller ofullständig information ges, måste skälen för detta noggrant anges.

Patienter och friska försökspersoner som anmäler sitt intresse att delta genom att besvara annonsen kontaktas per telefon av försöksledare på resp. centrer. De får därvid verbal information om projektet och skriftlig information (se bilaga) med informerat samtycke skickas hem till de tillsänkta försökspersonerna.

4:2 Hur och från vem inhämtas *samtycke*?

Beskriv proceduren; vem som frågar, när detta sker och hur samtycket dokumenteras. Utförlig redovisning är särskilt viktig då barn eller personer med nedsatt beslutskompetens ingår i studien, likaså vid studier av en grupp/grupper, t.ex. föreningar, organisationer, företag, kyrkosamfund, församlingar eller skolklasser.

Efter information via telefonsamtal skickas skriftlig information hem till de som anmält intresse. Skriftligt samtycke inhämtas innan försökspersonerna kan delta i studien.

5. Forskningsetiska överväganden

5:1 Redogör för de risker som deltagandet kan medföra samt möjliga komplikationer

Dessa kan vara t.ex. fysisk skada, smärta, obehag eller integritetsintrång som projektet innebär eller kan innebära. Ange vilka åtgärder som har vidtagits för att förebygga de risker som nämns ovan samt vilken

beredskap som finns för att hantera sådana komplikationer. Ange vilka/de metoder som kommer att användas för att efterforska, registrera och rapportera oönskade händelser.

Frågeformulären omfattar psykologiska, sociala och fysiska variabler och kan eventuellt upplevas som ett visst integritetsintrång. Försökspersonerna informeras om att alla data analyseras avidentifierade. Vår erfarenhet från tidigare studier är dock att patienter med smärta anser det viktigt att få förmedla sin situation och sina upplevelser. Vid höga poäng på Audit tas detta upp med försökspersonen som även får råd om var de kan vända sig för att få hjälp att minska sin alkoholkonsumtion.

Blodprovstagning följer kliniska rutiner och innebär ett smärre obehag.

Mikrodialys sker efter att huden har bedövats och innebär därför inte någon egentlig smärtsensation, förutom vid själva bedövningen. Alla mikrodialysundersökningar utförs i sjukhusmiljö med iakttagande av sedvanliga säkerhetsåtgärder och hygien för att minimera risken för komplikationer. Vid det statiska arbetet upplever vissa personer ökande smärta men denna är ej mer omfattande än vad som föreligger dagligen vid olika vardagliga aktiviteter. Försökspersonerna upplyses om att detta kan ske samt att smärtintensitesökningen snabbt klingar av. Några allvarliga komplikationer (tex infektioner) har inte förekommit i samband med mätningarna.

Hudbiopsierna utförs under bedövning och lämnar en liten sårskårpa 4-6 mm efter sig.

Smärttesten innebär att försökspersonen utsätts för smärtprovokation. Mestadels avbryts stimuleringen i samband med att smärttrösklen nåtts vilket innebär att försökspersonen utsätts för minimal smärta. Vid testning av övertröskelig smärtkänslighet för tryck och värme utsätts försökspersonen för kortvarig smärtstimulering över smärttröskeln, dvs utsätts för viss smärta. Smärtstimuleringen kan när som helst avbrytas av försöskpersonen. Någon allvarlig vävnadsskada har oss veterligen aldrig uppkommit och testen avbryts innan vävnadsskadande temperaturer eller tryck uppnås även om försökspersonen inte signalerar smärta.

Undersökning i fMRI kamera är oss veterligen helt ofarlig men kan upplevas som obehaglig av försökspersoner med tendens till klaustrofobi, varför detta utgör ett exklusionskriterium.

24-timmars EKG (HRV analys) är helt ofarlig för försökspersonen men det utgör ett visst obehag att sova med påklistrade EKG elektroder och Holterbandspelare intill sängen.

Träningsinterventionen till lika med avspänningsinterventionen är en del av rekommenderad behandling vid FM och helt anpassad till FM patienters förmåga. Om träningen medför ökad smärta reduceras träningsintensiteten tillfälligt. Behandlingen torde således snarast vara till godo för försökspersonerna. De friska försökspersoner som deltar i styrketräning torde även de få gynnsamma effekter av väl doserad fysisk aktivitet.

5:2 Redogör för förutsebar nytta för de forskningspersoner som ingår i projektet

Eftersom såväl träningsinterventionen som avspänningsinterventionen normalt ingår i rekommenderad behandling av FM torde de FM patienter som ingår i studien få del av de positiva behandlingseffekterna. Deltagandet i studien ger också en bra grund för fortsatt bra träningsaktivitet och en bättre förståelse för smärtsyndromet. Deltagarna lär sig att dosera träning med utgångspunkt från egna förutsättningar vilket ökar sannolikheten att de kommer att fortsätta vara träningsaktiva.

5:3 Gör en egen värdering av förhållandet risk - nytta för de forskningspersoner som deltar

FM är ett av de vanligaste smärtsyndromen hos kvinnor och orsakar stort lidande och nedsatt funktion och livskvalitet. Även om kunskapen om sjukdomsmekanismer ökat på senare år saknas fortfarande en förståelse för hur de olika sjukdomsmekanismerna samverkar och hur olika typer av behandlingar påverkar olika sjukdomsmekanismer och samverkan mellan dessa. Denna multicenterstudie har förutsättningar att belysa hur träning, dvs. förstahandsvalet vid behandling av FM påverkar en rad perifera och centrala sjukdomsmekanismer och samspelet mellan dessa. Försökspersonerna erbjuds en 15 veckor lång träningsintervention, ledd av specialutbildade sjukgymnaster och utan extra kostnader för försökspersonerna. Försökspersonerna kommer att få avsätta tid för besvarnade av frågeformulär och genomgå en del undersökningar som innebär viss smärta och visst obehag men oss veterligen inte lett till några allvarligare komplikationer. Det är sannolikt att deltagarna får glädje av tränings/avspänningsinterventionerna och att de i denna studie kan lägga grunden till varaktigt goda träningsvanor. Utöver detta får de en ökad förståelse för smärtsyndromet FM och får träffa andra med liknande besvär vilket brukar upplevas som positivt. Studien har goda förutsättningar att bidra till en ökad förståelse av sjukdomsmekanismer vid FM, vilket är nödvändigt för utveckling av mer effektiva behandlingar i framtiden. Sammantaget anser vi att det obehag som försökspersonerna utsätts för vida övervägs av den vetenskapliga nyttan med denna studie.

5:4 Identifiera och precisera om etiska problem t.ex risk - nytta i ett vidare perspektiv kan uppstå inom eller genom projektet

Här kan redovisas om exempelvis vissa grupper kan komma att utpekas/få hjälp som ett resultat av studien.

Studien har förutsättningar att öka förståelsen för hur fysisk aktivitet påverkar smärtmekanismer vid FM vilket förutom för framtida behandling av FM även kan vara av värde för att förstå varför arbetsförmågan påverkas negativt hos människor med FM och vilka åtgärder som skulle kunna vara effektiva för att motverka detta. Ökad förståelse för genetiska mekanismers betydelse för

smärtreglering och effekt av träning kan öka möjligheten att bättre anpassa behandlingar utifrån en individs givna förutsättningar.

6. Redovisning av resultaten

6:1 Hur garanteras forskningshuvudmannen och medverkande forskare tillgång till data (anges vid t.ex. uppdragsforskning) och vem ansvarar för databearbetning och rapportskrivning?

(Info: p. 6:1 i Vägledning till ansökan)

Deltagande forskare ansvarar för att studieresultaten publiceras i vetenskapliga tidsskrifter.

6:2 Hur kommer resultaten att göras offentligt tillgängliga? Kommer studien att insändas för publicering i tidskrift eller publiceras på annat sätt?

Ange i vilken form resultaten planeras offentliggöras samt tidsplan för detta.

Resultat kommer att redovisas på vetenskapliga kongresser och därefter publiceras i vetenskapliga tidsskrifter. Datainsamling 2010-2012. Databearbetning sker under 2013.

6:3 På vilket sätt garanteras forskningspersonernas rätt till integritet när materialet offentliggörs/publiceras?

Redovisas resultat på statistisk gruppnivå? Beskriv procedurer eller metoder för avidentifiering/anonymisering.

Alla data analyseras avidentifierade och resultaten presenteras enbart på gruppnivå.

7. Redovisning av ekonomiska förhållanden och beroendeförhållanden

Redovisning enligt punkterna 7:1-7:3 syftar till att tydliggöra alla direkta eller indirekta förhållanden, som kan tänkas påverka forskarens relation till forskningspersonerna (vid t.ex. informations-, samtyckes-, genomförandeprocedurer).

7:1 Vid uppdragsforskning

Ange uppdragsgivaren t.ex. ett företag (vid klinisk läkemedelsprövning eller prövning av andra nya produkter), en organisation eller en myndighet.

Namn: Kontaktperson:

Adress: Telefon/mobiltelefon:

Ange uppdragsgivarens relation till forskningshuvudmannen/medverkande forskare, t.ex. anställningsförhållande

7:2 Redovisa eventuella ekonomiska överenskommelser med uppdragsgivare eller andra finansiärer (namn, belopp)

Vid klinisk läkemedelsprövning bör hänvisning ske till ingånget avtal med sjukvårdshuvudmannen. Liknande överenskommelser kan förekomma vid annan uppdragsforskning och ska redovisas på samma sätt. Separata överenskommelser med den/de som ska genomföra forskningen ska redovisas. Belopp som kommer att erhållas för studien/ersättning till kliniken/genomföraren, vad ersättningen ska täcka och ev. belopp som erhålls per forskningsperson, ska också anges här (se p. 9 bilaga nr 12).

7:3 Redovisa forskningshuvudmannens, huvudansvarig forskares och medverkande forskares egna intressen

Här redovisas t.ex. aktieinnehav, anställning, konsultuppdrag i finansierande företag, eget företag som kan få (direkt eller indirekt) ekonomisk vinst av forskningen (se p. 9 bilaga nr 12).

Inga.

8. Undertecknande

Behörig företrädare för sökande forskningshuvudman enligt p. 1:2

Ort: Stockholm Datum: 10 09 10

Signatur:

Namnförtydligande: Jan Hillert

Tjänstetitel: Prefekt Institutionen för Klinisk Neurovetenskap

Undertecknad forskare som genomför projektet (kontaktperson) enligt p. 1:3 intygar härmed att forskningen kommer att genomföras i enlighet med ansökan

Ort: Stockholm Datum: 100908

Signatur: ______________________

Namnförtydligande: Eva Kosek

Tjänstetitel: Klinisk Lektor, Docent, Överläkare

9. Förteckning över bilagor (Info: p. 9 i Vägledning till ansökan)

Dokument som, i tillämpliga fall, ska bifogas *om inte motsvarande information finns i blanketten* har markerats med x. Markera de bilagor som skickas in med denna ansökan.

Insänd med ansökan	Bil nr	Beskrivning	Klinisk läkemedels-prövning	Annan forskning
☐	1	Deltagande forskningshuvudmän och medverkande forskare (kontaktpersoner) vid forskning där mer än en forskningshuvudman deltar. Info p. 1:5	X	X
☒	2	För fackmän avsedd forskningsplan, vid behov även för lekmän avsedd bilaga. Info p. 2:1 och i Vägledning till forskningsplan/forskningsprotokoll (program)	X	X
☒	3	Annonsmaterial för rekrytering av forskningspersoner. Info p. 3:1 och i Vägledning till ansökan p. 3:1	X	X
☒	4	Skriftlig information till dem som tillfrågas. Info p. 4:1 och i Forskningspersonsinformation	X	X
☒	5	Enkät, frågeformulär. Info p. 2:4	X	X
☐	6	Gemensam EU blankett (gäller fr.o.m. den 1 maj 2004), gäller även vid ändring.	X	
☐	7	Sammanfattning av protokollet på svenska	X	
☐	8	Prövarhandbok alt. bipacksedel/produktresumé/IB	X	
☒	9	Intyg från verksamhetschef/motsv. om resurser och om forskningspersonernas säkerhet. Info p. 2:6	X	X
☒	10	CV för forskare (samma som p. 1:3) med huvudansvar för genomförandet, redovisa forskarens (-arnas) kompetens av relevans för studien. Info i Vägledning till ansökan p. 1:3	X	X
☒	11	Beskrivning av ersättning till forskningspersoner. Info p. 3:6 och i Vägledning till ansökan p. 3:6	X	X
☐	12	Överenskommelser med uppdragsgivare/finansiär om t.ex. anställningsförhållanden, bidrag/ersättning till prövningsplats, sjukvårdshuvudman, forskningshuvudman eller forskare. Info p. 7:2 och p. 7:3	X	X

Övriga bilagor som bifogas ansökan:

Beskrivning av träningsinterventionen

Br 12

The effect of physical exercise on fibromyalgia symptoms – how do peripheral and central pain mechanisms interact and what is the impact of genetic factors?

Purpose and aims

FM is a chronic pain syndrome characterized by widespread, mainly muscular pain, tenderness, stiffness, disturbed sleep and fatigue. The prevalence is approximately 2% in the general population, 80% women. Currently FM is a dominating cause of long-term sick leave among female patients with musculoskeletal disorders in Sweden and FM patients rate their quality of life as very poor. Although recognized by the World Health Organization (WHO) as a diagnostic entity (ICD M79.7), FM remains controversial partly due to the lack of consensus regarding pathophysiology.

The pathophysiology of FM is most likely multifactorial with a complex interaction between peripheral and central mechanisms. Peripheral mechanisms, i.e., muscle ischemia and release of algogenic, pro-inflammatory substances have been documented in FM patients. Furthermore, central abnormalities i.e., dysregulation of the autonomuic nervous system and a dysfunction of endogenous pain regulatory mechanisms leading to pain amplification have been reported.

Several Swedish research groups, including the four groups represented in this collaboration, have made important contributions to the understanding of peripheral and central pain mechanisms in FM. The multifactorial nature of FM calls for expertise within various, traditionally independent research areas as well as access to advanced and costly equipment. Unfortunately, FM research has been characterized by research groups studying FM from their own perspective, with very limited translational collaboration internationally as well as in Sweden. The consequence is that although several peripheral as well as central abnormalities have been recognized in FM patients, understanding of how these mechanisms interact and how they are affected by various treatments is almost totally lacking.

The multifactorial nature of FM requires a multiprofessional treatment including physical therapy (training and relaxation exercise), cognitive behavioral therapy and pharmacotherapy (at present 3 drugs have approved indication for FM in USA, but none in Europe). However, currently no effective treatment is available for the majority of FM patients. In addition, there is no way to predict whether a patient is likely to benefit from a certain treatment or not, making FM treatment much of a trail and error procedure. Increased understanding of how specific treatments affect pathophysiological mechanisms in FM is therefore necessary for future development of effective treatments and to enable rational treatment choices.

The current project is a collaboration between four Swedish, internationally recognized research groups within FM research, two specialized on peripheral mechanisms (Prof. Björn Gerdle, Linköpings University and Assoc. Prof. Malin Ernberg, Karolinska Institute), one on central mechanisms (Assoc. Prof. Eva Kosek, Karolinska Institute) and one on the consequences of FM on psychological and physical symptoms, function, quality of life and ability to work and the impact of physical therapy on these parameters (Assoc. Prof.

Kaisa Mannerkorpi). In this translational research project, we wish to profit from the positive synergistic effects of collaboration between pre-clinical and clinical scientists as well as between researchers focusing on peripheral and central pain mechanisms. The collaboration has been made possible by an initiative of the Swedish Rheumatism Association to support clinically important pain research projects, this project being identified as one of the of them. A patient representative ("brukare") has participated in the planning of the project and is currently part of the research team in order to ascertain focus on patient perspective.

The broad spectrum of competence among the participating researchers in this project provides a unique opportunity to perform an in depth study of the interaction between peripheral and central pain mechanisms before and following a standardized training regime in patients with chronic widespread pain fulfilling the diagnostic criteria of FM. The study is planned as a randomized, controlled, multicenter trial (Gothenburg, Linköping, Stockholm). The extensive collaboration is necessary in order to provide the group with necessary competence and methodological know-how. In addition, this collaboration permits sharing of resources such as equipment and personnel and joint development of new analytic methods. The multicenter nature of this study enables fast recruitment of participants and makes the subjects representative of Swedish FM patients. We aim to include a large number of subjects which is a prerequisite for the multivariate statistical analysis necessary to analyze interactions between various pain mechanisms.

Specific aims

The purpose of this multicenter, randomized controlled trail (RCT) is to assess the effect of the most commonly recommended treatment for FM, i.e., standardized physical training, on pathopysiological mechamisms and clinical symptoms. We hypothesize that:

I) The pain in FM is related to increased sympathetic tone and reduced muscle blood flow giving rise to increased intramuscular concentrations of anaerobic metabolites as well as other pain inducing and pro-inflammatory substances.

II) The increased pain sensitivity and the dysfunction of endogenous pain inhibitory mechanisms in FM patients depend upon dysfunctional opioid and endocannabinoid systems and are influenced by certain genetic factors regulating the metabolism of transmitter substances involved in pain modulation.

III) Physical exercise diminishes the degree of symptom severity and activity limitations and improves body functions, work capacity and quality of life.

IV) Physical exercise normalizes the sympathetic tone and improves muscle blood flow thus reducing intramuscular concentrations of anaerobic metabolites, pain inducing and pro-inflammatory substances.

V) Physical exercise improves the function of endogenous pain inhibitory mechanisms, but this effect is less pronounced in certain genotypes due to an inherited trait of reduced efficiency of endogenous opioid and cannabinoid pain inhibitory systems.

Survey of the field

Epidemiology and diagnosis

Chronic generalized pain is a heterogenous condition affecting approximately 10 % of the general population (1). Fibromyalgia (FM) is the only form of generalized pain with internationally accepted classification criteria and also represents the most severe form of generalized pain including generalized allodynia/hyperalgesia for mechanical stimuli (pressure) (2). The diagnosis of FM is based on the American College of Rheumatology classification criteria (3) and relies on the presence of chronic widespread pain and tenderness (i.e., allodynia to pressure), in addition, fatigue, disturbed sleep and stiffness is typically present. The prevalence rates are fairly homogenous across various continents and correspond to approximately 2% of the population, making FM one of the most common pain syndromes in man (3).

FM has a great impact on the quality of life and work performance. Swedish FM patients had lower quality of life ratings than patients with rheumatoid arthritis (4) and FM caused more days away from work than did inflammatory rheumatic diseases (5).

Even though FM is recognized by the WHO and classified as a diagnostic entity according to ICD 10 (M79.7), certain health care practitioners still fail to recognize FM as a valid concept. Many patients with FM report great difficulties to get their symptoms acknowledged and treated, which is a likely explanation for the large number of visits to various health care providers that these patients make. The cost of health care for FM in Sweden corresponded to 865 million SEK/year, while the costs for sick-leave corresponded to 12.5 billion SEK/year (5). Recent studies from primary care settings in UK show that the yearly health care costs were reduced for 4 years following the diagnosis of FM compared to a ten year period prior to FM diagnosis (6), stressing the importance to increase the willingness to recognize FM in the health care system.

Pain mechanisms in FM

Muscle ischemia and neurogenic inflammation

FM is a multisymptomatic pain syndrome, the pain most likely caused by a complex interaction between peripheral and central mechanisms (7). In this perspective the autonomic nervous system is of interest, since it is involved in the regulation of muscular blood flow as well as physiologically closely linked to endogenous pain modulatory mechanisms (7). FM patients have been shown to have an increased sympathetic nervous system activity at baseline combined with hypo-reactivity during exposure to various stressors, including physical activity (8, 9). The latter could have a negative influence on muscle blood flow. Muscle ischemia has been identified as a potent cause of peripheral sensitization, leading to increased excitability and spontaneous activity of peripheral nociceptors and thus potentially explaining muscle pain during physical activity and rest (10). In FM muscle ischemia has been proposed to form the basis of peripheral sensitization based on findings of reduced intramuscular blood flow during physical activity (11) and morphological muscle abnormalities typical of muscle ischemia (12). Furthermore, increased intramuscular concentrations of the anaerobic metabolites lacatate and pyruvate were found in FM patients (Gerdle et al submitted), with possible implications for increased nociception (13). Activation of primary afferent nociceptors leads to neurogenic inflammation with efferent release of algogenic substances such as glutamate, substance P (SP), calcitonin-gene-related peptide (CGRP), serotonin (5-HT), and nerve growth factor (NGF). In humans,

intramuscular (i.m.) injections of glutamate (14), SP/CGRP (15), 5-HT (16) and NGF (17) induce pain. Furthermore, increased pain sensitivity (allodynia/hyperalgesia) has been reported following i.m. injections of glutamate (14), 5-HT (16), SP when co-injected with bradykinin (18) and NGF (17).

Given that muscular pain and increased pain sensitivity are the hallmarks of FM, the potential implication of these substances in pathogenesis of FM are of interest. In fact, researchers in this project have documented increased concentrations of glutamate in patients with chronic localized/regional muscular pain (19, 20). To our knowledge, we were also first to report that 5-HT was released in higher concentration in the muscle of FM patients compared to healthy controls (21). We are not aware of any publications regarding the peripheral release of SP/CGRP or NGF in FM patients. Experimentally, intramuscular injection of NGF in humans causes mild transient pain but a long-lasting (at least 7 days) mechanical hyperalgesia as well as pain during strenuous movement (17), expansion of the hyperalgesia to distant areas (22) and increased pain to temporal summation (23). Given the presence of generalized hyperalgesia, increased pain during exercise and increased pain to temporal summation in FM patients, NGF could potentially be implicated in FM. NGF may also be transported retrogradely and regulate receptor proteins in the periphery, i.e., SP and CGRP receptors (24). Although, as stated, we are not aware of any studies assessing NGF in peripheral tissues in FM patients, elevated NGF concentrations have been found in the cerebrospinal fluid (CSF) of FM patients and to correlate with increased glutamate levels in the CSF (25). Thus, both peripheral and central mechanisms could be involved in conditions with NGF-induced hyperalgesia, which is of great interest since NGF-antagonists are presently being explored as a possible treatment for chronic pain (26).

Autonomic nervous system and cytokines

Increased sympathetic tone has been related to pain and associated with the increased release of certain pro-inflammatory cytokines, such as IL-8 (27). Several studies have shown increased levels of IL-8 in the blood of FM patients that can be reduced following physical exercise (28, 29). Although not a uniform finding (28) IL-8 has been positively related to ongoing pain in FM patients (30). Parasympathetic activity has been attributed anti-inflammatory effects (i.e., the vagal anti-inflammatory reflex) (31). We have recently documented a relative hypoactivity of the parasympathetic NS in FM patients, which would theoretically favor the release of pro-inflammatory cytokines in relation to anti-inflammatory cytokines. This is in accordance with previous studies where increased concentrations of pro-inflammatory cytokines (TNF-alpha, IL-1b, IL-8) (32) as well as decreased concentrations of anti-inflammatory cytokines (IL-4, IL-10) (33) have been reported in the blood of FM patients. Given that FM patients mainly complain of muscular pain and exacerbation of pain during and following physical activity, the study of intramuscular concentrations of cytokines would be highly relevant. To our knowledge, no previous study has assessed the intramuscular release of cytokines in FM patients.

The increased sympathetic tone in FM patients would have negative consequences on muscle blood flow causing muscle ischemia, neurogenic inflammation and thus increased intramuscular release of algogenic substances such as glutamate, SP, CGRP, 5-HT and NGF as well as pro-inflammatory cytokines (TNF-alpha, IL-1b, IL-8) (I). As an explorative part of this study we also intend to apply proteomics (34) to microdialysate and serum in order to identify potentially unknown proteins relevant for pain perception in FM and to study how these proteins are expressed and regulated (35). Proteomics have contributed to the understanding of inflammatory and neuropathic pain (36) and to the

identification of potentially new peptides of interest in the cerebrospinal fluid of patients with generalized pain and fatigue (37), but, to our knowledge, proteomics have never been studied in a well defined FM population. The i.m. concentrations of algogenic and proinflammatory substances will be related to clinical symptoms such as pain, tenderness and exercise intolerance. The increased afferent input from sensitized muscle nociceptors has been suggested to initiate and maintain the central dysfunction, i.e., central sensitization, disinhibition and amplification of nociceptive input, all leading to pain amplification in FM patients (7). Therefore, we believe that the degree of peripheral pathology described above will be related to the degree of central dysfunction such as increased temporal pain summation and decreased function of endogenous pain inhibitory mechanisms.

Central pain mechanisms

CNS mechanisms in FM have received increasing attention during the past decades. FM patients suffer from a generalized, non-modality specific increase in pain sensitivity (38) and increased temporal summation of nociceptive stimuli indicating central sensitization (39). Behavioural studies have shown a dysfunction of endogenous pain inhibitory mechanisms (40), supporting CNS dysfunction of pain regulation. Using a specially developed technique for assessment of pain related brain activation with functional magnetic resonance imaging (fMRI) we have recently documented that when FM patients were compared to healthy controls, lower pressure stimulation intensities were sufficient to evoke the same amount of subjective pain and no group differences in activation of the ascending nociceptive projection areas were found, providing objective proof of increased transmission and/or processing of nociceptive input in FM (41). However, contrary to healthy controls, FM patients failed to activate the first link in the descending pain regulatory system (rostral anterior cinguate cortex, rACC) and the regions in the brainstem which are known to depend on the rACC displayed a corresponding abnormality in the activation pattern. (41). To our knowledge, this is the first time that a dysfunction in a specific brain area involved in pain modulation has been identified in FM patients. The rACC is well known for its executive pain modulatory function (42, 43), constitutes a high concentration of opioid receptors and is central in opioid-dependant treatments including those relying on activation of endogenous pain inhibition such as placebo analgesia (42). The inability to activate the descending inhibitory system during provoked pain and the possible impairment of its opioid receptors is consistent with reports of reduced mu-opioid binding capacity in these regions in FM patients (44) as well as with reports of FM patient's poor response to this class of analgesics (45). A possible alternative interpretation of the nature of the inhibitory impairment in FM would be that the down-regulation of the my-opioid receptors is a response to a constant nociceptive input (44), i.e. constant baseline activation of the pain regulatory mechanisms could preclude further activation during experimental pain stimulation as well as in situations when the system is normally activated such as during physical exercise.

We have previously documented a dysfunction of endogenous pain inhibitory mechanisms during static work in FM patients (46), which has been confirmed in later studies (47). Furthermore, our preliminary results indicate that FM patients have a hypoactive sympathetic nervous system and a hypo-reactive hypothalamic-pituitary-adrenal axis (HPA-axis) during static work, with a reduced release of adrenocorticotropic hormone (ACTH) (Kadetoff et al, unpublished data). Since endogenous opioids are co-released with ACTH, the latter might have direct implications for the reduced opioid mediated exercise induced analgesia in FM patients. Exercise induced analgesia has been shown to be partly opioid dependent, but endocannabinoids have also been implicated (48). Endocannabinoids act synergistically with opioids in the pain regulatory systems but in addition they also have pain

inhibitory effects that are mediated through opioid independent mechanisms (48). Endocannabinoids are endogenous lipid mediators that pass the blood-brain-barrier and regulate pain and inflammation. To date, two main endocannabinoid ligands have been identified in humans, i.e., anandamide and 2-arachidonoylglycerol (2-AG) (48). Anandamide is released into the circulation following nociceptive stimulation and exercise (48). Conflicting reports exist regarding anandamide in FM. Increased concentrations of anandamide have been reported in the blood of FM patients but were not related to FM symptoms (49), on the other hand, treatment with anadamide was beneficial in reducing pain and improving function in FM patients (50). We are not aware of any reports regarding 2-AG in FM patients. Our hypothesis is that FM patients, as a response to peripheral nociceptive input, have a long-term activation of endogenous cannabinoid and opioid pain inhibitory systems. The long-term activation causes down-regulation of sensitivity in these systems and therefore a relative inability to activate them during pain stimulation and exercise (**II**). Therefore, we wish to examine the baseline levels of endogenous opioids and endocannabinoids as well as the expression of their receptors. In addition, we wish to examine the response of the opioid and endocannabinoid system to physical activity and to relate this to perception of pain and to pain sensitivity. The importance of certain genetic factors known to influence the efficacy of opioid dependent pain modulatory mechanisms will be assessed (**II**).

Genetics and fibromyalgia

Although no specific genes have been identified to cause FM, certain genetic polymorphisms are over represented in FM patients as well as in patients with other chronic pain syndromes compared to the general population. The two most important functional polymorphisms identified in FM patients are the (val158met) of the Catechol-Omethyltransferase (COMT) gene that regulates the metabolism of dopamine/noradrenaline and two polymorphisms (SERTPR; 5-HTTLPR and rs25531) affecting the expression of the serotonin transporter (SERT) which is a key regulator of serotonin metabolism and availability (51). Subjects homozygous for the COMT met/met and/or 5-HTTLPR s/s are overrepresented among patients suffering from chronic pain, including FM (51) opioid addiction and depression/anxiety (52, 53). Furthermore, healthy individuals homozygous for the COMT met/met have been reported to have increased pain sensitivity and a reduced activation of endogenous opioidergic pain inhibitory mechanisms compared to subjects homozygous for COMT val/val (54). Recently, we investigated the influence of genetic factors on the pain relieving effect of a short acting opioid (remifentanyl i.v.) in healthy subjects. We found that the functional SERTPR polymorphisms clearly related to the pain relieving effect of remifentanyl (53), while the functional COMT polymorphisms were related to opioid induced hyperalgiesia/central sensitization (52). Our results firmly indicate the importance of these two functional polymorphisms for the function of opioid pain modulatory mechanisms. Since opioid drugs work on the same receptors as the endogenous opioids, our results indicate that these functional polymorphisms are most likely also of importance for the function of endogenous pain inhibitory mechanisms. We hypothesize that FM patients homozygous for the functionally low expressing haplotypes (including COMTmet/met and SERTPR; 5-HTTLPR and rs25531) will have a reduced function of endogenous pain inhibitory mechanisms, leading to; 1) increased clinical symptoms, 2) more pronounced exercise intolerance, 3) reduced activation of endogenous pain inhibitory mechanisms during exercise related to reduced peripheral expression of opioid and endocannabinoid receptors, and 4) normal (or increased) baseline release of opioids and endocannabinoids but reduced release during physical activity (**II**).

Fibromyalgia treatment

The increased understanding of the CNS mechanisms in FM has had direct consequences for treatment development and several centrally acting drugs have been approved for FM treatment in the USA (but not in Europe). However, since pharmacological treatments remain effective only in a minority of FM patients, the core of FM treatment still relies on multidisciplinary treatments including cognitive behavioral therapy and physical therapy (i.e., multimodal rehabilitation programs). In fact, physical therapy is the most frequent management of patients with chronic musculoskelettal pain (55, 56, 57) and forms an integral part in the treatment and rehabilitation of FM patients (58). Physical therapy regimes including exercise tailored according to the specific needs of FM patients have been shown to be beneficial (57). However, the effects of exercise on the pathophysiological mechanisms in FM are not well understood. Exercise has been demonstrated to improve muscle blood flow (59), reduce sympathetic tone and permit more intensive physical activity during the same level of sympathetic nervous system activation in healthy subjects (60). Furthermore, pain inhibitory mechanisms that are normally activated during exercise have been demonstrated to be dysfunctional in FM patients (46, 47) and it is unknown if exercise can normalize their function. Prospective studies are needed to establish the effects of exercise on peripheral as well as central pain mechanisms in FM patients. We expect that standardized physical training will have long-term beneficial effects i.e., diminished pain and improved function in FM patients compared to relaxation exercises (**III**). Our hypothesis is that the positive effect of exercise in FM is characterized by a normalization of sympathetic nervous system activity and improved muscle blood flow which would hypothetically lead to reduced intramuscular release of anaerobic metabolites, pain inducing substances and pro-inflammatory cytokines at baseline as well as following physical activity (**IV**). Finally, we hypothesize that exercise will improve the function of endogenous pain inhibitory mechanisms but that this improvement will be less pronounced in FM patients homozygous for the functionally low expressing haplotypes of COMT and SERT due to their negative influence on the function of pain regulatory mechanisms involving endogenous opioids and cannabinoids (**V**). In this multicenter, translational study we hope to profit from the synergistic effects between the various research groups to clarify the effects of physical training on the pathogenesis of FM and to find prognostic markers useful for pain treatment and rehabilitation of FM patients.

Project description

Overview

This translational study will be performed as a randomized, controlled, multicenter assessment of the effects of standardized physical training on symptoms, function, work capacity and quality of life in FM patients. Muscle metabolites (lactate, puruvate), inflammatory parameters (cytokines), algesic substances (glutamate, SP, CGRP, 5-HT, NGF) and analgesic substances (endogenous opioids and cannabinoids) will be measured in muscle (using microdialysis technique) and blood in FM patients and compared to healthy controls before and following exercise. In addition, the effect of physical exercise on autonomic nervous system activity (using HRV) and endogenous pain modulation (using quantitative sensory testing (QST) and fMRI) will be assessed. Finally, we also plan to analyze the impact of specific genetic haplotypes on the above mentioned parameters. Three centers, each representing specific competence in the various research areas will participate (i.e.,

Sahlgrenska University Hospital, Gothenburg, Linköpings University, Linköping and Karolinska Institute, Stockholm).

Subjects

A total of 144 female FM patients, age 20-65 years and fulfilling the ACR classification criteria for FM (3) and 144 healthy female age-matched controls will be included. Subjects will be recruited by newspaper advertising in order to avoid bias towards psychosocially affected patients that are overrepresented at highly specialized pain centers. Patients will be allowed to continue with their baseline medication but analgesics and NSAIDs will be suspended 48 hours prior to investigations (assessment of pain sensitivity, HRV, microdialysis/skin biopsy, fMRI). 48 FM patients and 48 controls will be recruited in each center (Gothenburg, Linköping, Stockholm).

Procedures

The FM patients will be randomized to active physical training (n = 72) or relaxation exercise (i.e., control intervention, n = 72) (see flowchart). The healthy subjects will be randomized to physical training (n = 36) or baseline controls (n = 108). The physical training/relaxation exercise will be performed twice a week for 15 weeks under the supervision of specifically trained physical therapists. Self administered validated questionnaires will be used to assess symptoms, function and quality of life at baseline and to follow intervention. Blood samples (all subjects) and muscle microdialysis with skin biopsy (m. quadriceps femoris) (36 FM patients and 36 controls) will be taken at baseline and following training for assessment of substances of importance for muscle metabolism, pain modulation and inflammatory responses. Samples of saliva will also be used for genotyping for genetic polymorphisms of relevance for pain modulation. The autonomic function will be assessed using a 24 hour analysis of heart rate variability at baseline and following training (72 FM patients and 72 controls). Pain sensitivity (using pressure algometry) and the function of endogenous pain modulatory mechanisms during physical activity, (i.e., the difference between pressure pain sensitivity at baseline and during standardized static contractions) will be monitored at baseline and following intervention in all subjects. Furthermore, temporal heat summation (Thermotest), a measure of central pain processing, will be assessed in all subjects at baseline and following physical training and related to the specific genotypes. 24 FM patients and 12 controls will also be assessed using fMRI to assess pain related cerebral activation and function of endogenous pain inhibitory mechanisms before and following treatment (physical exercise). Furthermore, as an exploratory part of this study protein analysis (i.e., proteomics) will be performed in microdialysate and blood in order to identify specific pathological profiles in FM patients.

Flowchart

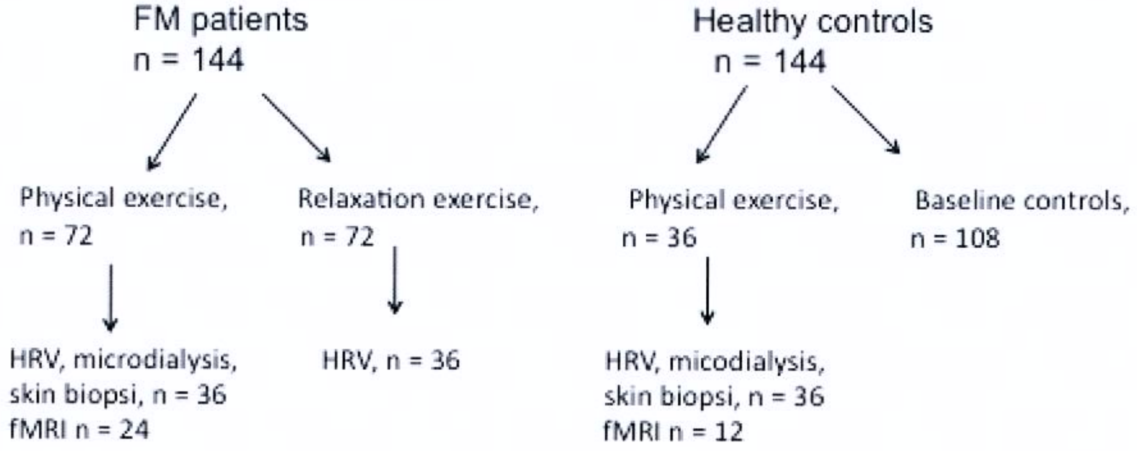

Methods

Questionnaires

Patients will be asked to rate the intensity of pain on a 100 mmVAS. Present pain, as well as average, minimal and maximal pain during the past week will be registered. The distribution of pain will be assessed using a standardized pain drawing and patients will complete the pain disability index (PDI). Psychological parameters and coping will be assessed by hospital depression and anxiety scale (HADS), pain catastrophizing scale (PCS), chronic pain acceptance questionnaire (CPAQ) and fear-avoidance beliefs questionnaire (FABQ). Various aspects regarding physical activity and performance will be assessed by exercise self-efficacy scale, experience of physical activity (EPA), perceived physical work demands (Balogh instrument) and leisure time physical activity (LTPAI). FM symptoms and the impact of FM will be assessed by Pittsburg sleep quality index (PSQI), multidimensional fatigue inventory (MFI), fibromyalgia impact questionnaire (FIQ) and short form health survey (SF-36). Effect of treatment will also be monitored by the one-question patient global impression of change (PGIC).

Venous blood samples

Blood samples will be drawn for analysis of endogenous opioids and endocannabinoids (anandamide and 2-AG), cytokines (TNF-alpha, IL-1b, IL-6, IL-8, IL-4 and IL-10), NGF, SP, CGRP, 5-HT, catecholamines, ACTH, glutamate, lactate, pyruvate, vitamin D and proteomics. Mononuclear cells will be separated for the analysis of opioid (beta-endorphin, endomorphin 1 and 2, dynorphin, leu-and metenkephalins) and cannabinoid (anandamide and 2-arachidonoyl glycerol) ligands and their receptors (mu, delta, kappa and sigma as well as CB1 and CB2).

Saliva samples

DNA will be extracted from blood samples and genotyped regarding relevant polymorphisms (SERT, COMT, OPRM 1 and SCN9A).

Intramuscular microdialysis

The intramuscular microdialysis method developed by researchers in this group will be used (19, 61) and microdialysate will be analysed for lactate, pyruvate, glutamate, SP, CGRP, 5-HT, NGF, BDNF, cytokines (TNF-alpha, IL-1b, IL-6, IL-8, IL-4 and IL-10), endogenous opioids and endocannabinoids as well as catecholamines. In addition proteomics will be performed.

Proteomics

Characterization of the plasma and muscle microdialysate proteome will be done by using 2-DE / MS. 2-DE resolves complex protein mixture in the first dimension by charge and in the second dimension by mass. Quantification of the separated proteins is achieved by spot integration using specialized 2-DE software that makes it possible to compare protein patterns from different subjects/groups. Proteins are identified by in-gel digestion and peptide mass fingerprinting using MALDI-TOF (matrix assisted laser desorption ionization time of flight) or amino acid sequencing using ESI-Q/TOF (electrospray ionization triple quadrupole time of flight) MS.

Skin biopsy

The material will be analysed for expression of cannabinoid receptors (CB1 and CB2 and NMDA-receptor (glutamate)).

Functional tests

The distance that a person can walk during 6 minutes (6-minute walk test (62)), maximal voluntary contraction force (MVC) during isometric contractions in knee extensors (Stigstarke®), elbow flexors (Isobex®) and hand grip (Grippit®) as well as a functional standing balance test (63) will be assessed before and following treatment.

Assessment of pain sensitivity and endogenous pain inhibitory mechanisms

Pressure pain thresholds (PPTs) will be assessed at 4 bilateral sites using a pressure algometer (Somedic Sales). In addition PPTs will be assessed at the right thigh (m. quadriceps femoris) and at the left shoulder (m. deltoideus) before and every 30 seconds during an isometric knee extension corresponding to 25 % of the individual maximal voluntary contraction force. The increase in PPTs from baseline will be calculated as a measure of the activation of endogenous pain inhibitory mechanisms (46).

Heat and cold pain thresholds and temporal summation will be assessed using a thermotest (Metronics or Somedic). Tonic heat stimulation corresponding to individually calibrated suprathreshold (average between Borg 4/10 and 7/10 rating) will be presented for 2 minutes and subjects will continiously rate the pain on a VAS. The increase in VAS ratings from start to end is taken as a measure of temporal summation (central sensitization)(64).

Heart rate variability (HRV)

A 24- hour spectal analysis of heart rate variability (Holter-ECG) will be performed as one parameter assessing autonomic nervous system tone, specifically diurnal variation will be analyzed.

fMRI examinations

Pressure stimulation will be applied to the thumbnail using an automated, pneumatic, computer-controlled stimulator (Pepito) used previously in our fMRI studies (41) and each subject will be calibrated for subjective pain ratings corresponding to 50 mm on a 100 mm VAS. These individually calibrated painful stimuli will be used during the fMRI examinations. Images will be collected using a 1.5 Tesla scanner. Multiple T2-weighted single-shot gradient echo EPI sequences will be used to acquire the blood oxygen level dependent (BOLD) contrast images. In addition to the functional scans, high resolution T1-weighted structural images will be acquired in coronal orientation for anatomical reference purposes and screening for cerebral anomalies.

Randomization

Patients will be recruited separately for each center. Informed consent will be collected for all subjects for participation in the entire study (including microdialysis/skin biopsi) (A) or all investigations except micordialysis/skin biopsi (B). Randomization will take place at each center stratified for group A and B, respectively.

Physical exercise

Low-intensity of exercise, adjusted to the individual limitations, has shown to work well in most patients with FM (65). For this study, we have chosen strength training which is initiated at low loads, at 40% of the maximum volontary capacity (MVC), and successively progresses to 70-80% of MVC. This exercise mode has previously shown to be feasible in FM (66). Exercise load will be measured and monitored for each subject. Sessions will start with a 10 min warm-up (stationary bicycle), followed by a 50 min strength training protocol. All three centers will be coordinated by the same investigator (Mannerkorpi) to ensure that procedures and training equipment is standardized across sites.

Relaxation exercise

The session will be initiated with a 10 min stretching program to release muscle tension, after which a 50 min relaxation program (67) will be conducted lying supine. All three centers will be coordinated by the same investigator (Löfgren) to ensure that procedures are standardized across sites.

Significance of the research project

In order to reach a more holistic view of FM, a multi-factorial pain syndrome, increased understanding of the interactions between peripheral and central pain mechanisms is necessary. The project aims at answering highly relevant, but complex, research hypothesis and therefore calls for collaboration between various researchers each one successful in their respective field of pain research. Previous research regarding FM has often been hampered by the controversies between researchers advocating peripheral and those advocating central pain mechanisms. In this rather unique project we aim to recruit representatives of both views to work together. FM is a common chronic pain syndrome causing much suffering, loss of function, reduced work capacity and quality of life. Typically pain is exacerbated during and following physical activity. However, physical exercise is the most often recommended treatment in chronic pain (68) and has also been found beneficial in FM. Increased understanding of the interactions between peripheral and central pain mechanisms during physical activity as well as the effect of long-term standardized physical exercise on these mechanisms is necessary in order to improve treatment for this patient group. The importance of genetic factors for FM symptoms and treatment response needs to be investigated in order to allow for identification of individuals with increased risk of developing chronic generalized

pain and allowing for preventive measures. In addition, increased understanding of pathophysiological mechanism is a prerequisite for future development of more effective treatments.

References

1) Gran J. Best Practice & Research Clinical Rheumatology 2003;17(4):547–61,
2) Cöster L et al. Eur J Pain 2008;12:600-10,
3) Wolfe F, et al. Arthr Rheum 1990;33(2):160-72,
4) Bergman S. Dissertation. Lunds University, Sweden, 2002,
5) Schmidt et al. CMT Rapport 2003:5,
6) Annemans et al. Athritis & Rheum 2008;58(3):895-902,
7) Kosek E. Int J Clin Rheumatol 2009;4:493-7.
8) Cohen et al. IMAJ 2001;3:755-60,
9) Petzke F et al. Curr Rheumatol Rep 2000;2:116-23,
10) Mense S. Current Pain & Headache Rep 2003;7:419-25,
11) Elvin et al. Eur J Pain 2006;10(2):137-44,
12) Bengtsson A. Rheumatol 2002;41(7):721-24,
13) Kim et al. J Pain 2007;8:59-66,
14) Svensson et al. Pain 2003a;101:221-7,
15) Pedersen-Bjergaard et al. Peptides 1991;12:333-7,
16) Ernberg et al. Pain 2000;84:339-46,
17) Svensson et al. Pain 2003b;104:241-7,
18) Jensen et al. Cephalalgia 1991;11:175-82,
19) Rosendal et al. Pain 2004;112:324-34,
20) Larsson et al. Pain 2008;140:479-90,
21) Ernberg et al. Life Sci 1999;65:313-25,
22) Andersen et al. Exp Brain Res 2008;191: 371-82,
23) Nie H et al. Eur J Pain 2009;13 704-10,
24) Bennett. Neuroscientist 2001;7:13-17,
25) Sarchielli et al. J Pain 2007;8(9):737-45,
26) Watson et al. BioDrugs 2008;22(6):349-59,
27) Cunha et al. Br J Pharmacol. 1991;104:765-7,
28) Wang et al. J Rheumatol 2008;35(7):1366-70,
29) Ortega et al. Exerc Immunol Rev 2009;15:42-65,
30) Gur et al. J Rheumatol 2002;29(2):358-61,
31) Tracey. J Clin Invest 2007;117:289-296,
32) Bazzichi et al. Clin Exp Rheumatol 2007;25(2):225-30,
33) Uceyeler et al. Arthritis & Rheumatism 2006;54(8):2656-64,
34) Camafeitaet et al. Proteomics – clinical applications 2009;3:226-41,
35) Banks et al. Lancet 2000;356:1749-56,
36) Kunz et al. Neurosci Lett 2005;381:289-93,
37) Baraniuk et al. BMC Neurol 2005;1(5):22,
38) Kosek et al. Pain 1996a;68(2-3):375-83,
39) Staud et al. Pain 2001;91:165-75,
40) Kosek et al. Pain 1997;70(1):41-51,
41) Jensen et al. Pain 2009a;144(1-2):95-100,
42) Petrovic et al. Science 2002;295:1737–40,
43) Tracey and Mantyh. Neuron 2007(55):377-91,
44) Harris et al. J Neurosci 2007;27(37):10000-6,
45) Sörensen et al. Scand J Rheumatol 1995;24:360-65,
46) Kosek et al. Pain 1996b;64(3):415-23,
47) Staud R et al. Pain 2005;118(1-2):176-84,
48) Dietrich and McDaniel. Br J Sports Med 2004;38:536-41,
49) Kaufmann et al. Psychoneuroendocrinology 2008;33:676-85,

50) Skrabek et al. J Pain 2008;9(2):164-73,
51) Buskila. Best Pract Res Clin Rheumatol 2007;21(3):535-47,
52) Jensen et al. PLoS One 2009b;4(6):e6016,
53) Kosek et al. Mol Pain 2009;5:37,
54) Zubieta et al. Science 2003;299:1240-43,
55) Dziedzic et al. Best Practice & Research Clinical Rheumatology 2008;22(3):407-18,
56) Andersen et al. J Appl Physiol 2008;105:1796-1801,
57) Mannerkorpi and Henriksson. Best Practice & Research Clinical Rheumatology 2007;21(3):513-34,
58) Carville et al. Ann Rheum Dis 2008;67(4):536-41,
59) Laughlin et al. J Physiol Pharmacol 2008;59(suppl 7):71-88,
60) Peronnet et al. J Appl Physiol 1981;51(4):812-15,
61) Sjøgaard et al. Eur J Appl Physiol 2010;108:657-69,
62) Mannerkorpi et al. Arthritis Care Res 1999;12(3):193-99,
63) Franchignoni et al. Aging 1998;10:26-31,
64) Tousignant-Laflamme et al. Brain Res 2008;1230:73-9,
65) Mannerkorpi et al. J Rehabil Med 2009;41:751-60,
66) Valkeinen et al. Arthritis Rheum 2006;54(4):1333-39,
67) Rudberg, B., Avspänning, ett sätt att leva. 1985: Natur och Kultur,
68) Gerdle B, Gullacksen A-C. Multimodal rehabilitering. Metoder för behandling av långvarig smärta – en systematisk litteraturöversikt 2006; 159-213 (SBU).

Study group:

Steering commettee:
The project steering commettee consists of group leaders of the participating research groups i.e., Assoc. Prof. Ernberg (Karolinska Institute, Huddinge), Prof. Gerdle (Linköpings University), Assoc. Prof. Kosek (Karolinska Institute, Solna) (PI) and Assoc. Prof. Mannerkorpi (Göteborgs University).

Participating researchers in alphabetic order:

Ernberg Malin, DDS, PhD, Assoc Prof.
Dr. Ernberg is a dentist. She defended her thesis on muscle pain in 1999, did her post-doc 2004-2006 in a distinguished laboratory in Århus, Denmark (Prof Svensson) and became Assoc. Prof 2004. She currently holds a position as senior consultant at the Karolinska University Hospital, Huddinge and has a 50% research position (VR, Swedish research Council). She has supervised one PhD student and is currently supervising five. She has published 40 scientific articles and 7 review articles/book chapters. Her main research area is muscle pain (neuropeptides, algogenic and inflammatory substances and micordialysis).

Gerdle Björn, MD., PhD., Professor.
Dr. Gerdle is a specialist in rehabilitation medicine. He defended his thesis in rehabilitation medicine 1985, became Assoc. Prof 1988 and Professor in Rehabilitation Medicine 1993. Currently he holds the position as Prof. of Rehabilitation Medicine at Linköpings University and Head of Department/Senior consultant at the Pain Center, Linköpings University Hospital. He has supervised 12 PhD students and is currently supervising three. He has published 143 articles in scientific journals and written 19 book chapters. His main research area is muscle pain (metabolites, muscle perfusion, microdialysis) and treatment/rehabilitaiton of chronic pain patients.

Ghafouri Bijar, PhD.
Dr. Ghafouri has a diploma as an ingenier in chemistry. She defended her thesis in experimental medicine 2005 and did her post-doc at Linköpings University 2006-2007. She currently holds a position as chemical ingenier at Östergötlands County Council. She has published 8 scientific articles. Her main research areas are methods for analysis of blood and microdialysis samples regarding algogenic and inflammatory substances and proteomics.

Kosek Eva, MD., PhD., Assoc. Prof.
Dr Kosek is a specialist in rehabilitation medicine and pain relief. She defended her thesis on fibromyalgia1996 and became Assoc Prof in pain research 2004. She currently holds a postition as Assoc. Prof. at Karolinska Institute, Solna (50%) and senior consultant Dept of Rheumatology, Karolinska University Hospital (currently placed at Stockholm Spine Center, 50%). She has supervised two PhD students and is currently supervising two and one post-doc. She has published 25 sceintific articles and 10 book chapters. Her main research area is endogenous pain regulation (central sensitization/disinhibition/facilitation, quantitative sensory testing and functional imaging).

Larsson, Britt, MD., PhD, Assoc Prof.
Dr Larsson is an MD specialized in occupational medicine. She defended her thesis 2001 regarding muscle pain and became Assoc. Prof. in clinical pain research 2007. She currently holds a postition as senior consultant at Pain and Rehabilitation Center, Linköpings Unviversity Hospital and as Assoc. Prof. in clinical pain research 50%. She has published 24 scientific articles. Her main area of research is muscular pain (regulation of muscle perfusion during physical exercise, microdialysis).

Ljungar Indre, MD., PhD.
Dr. Ljungar is doing her training to become a specialist in rehabilitaiton medicine at the Dept of Rehabilitation Medicine, Danderyds Hospital, Stockholm. She defended her thesis on inflammatory mechanisms 1998 and has published 23 scientific articles. Her main areas of research is peripheral inflammation, opioids and endocannabinoids.

Löfgren, Monika, PT., PhD.
Dr Löfgren is a physical therapist with a long experience in chronic pain. She defended her thesis on fibromyalgia 2006. She currently holds a position as physical therapist at the Dept of Rehabilitation Medicine, Danderyds Hospital, Stockholm. She is supervising 2 PhD students. She has published 6 scientific articles. Her main research area is the effect of physical therapy in fibromyalgia (body awareness/relaxation), psychosocial factors, assessment of function, work capacity and quality of life.

Mannerkorpi Kaisa, PT., PhD., Assoc Prof.
Dr. Mannerkorpi is a Physical Therapist with a long experience in chronic pain. She defended her thesis in physical therapy 1999, did her post-doc at Georgetown University, Washington DC 2001-2002 in collaboration with a distinguished researcher in fibromyalgia (Dr. D. Clauw) and become Assoc. Prof. 2006. She is currenlty holding a position as Assoc Prof and Physical Therapist at Sahlgrenska UniversityHospital and the University of Gothenburg, and has a 50% research position financed by the Swedish Research Council. She is currently supervising four PhD students. She has published 19 scientific articles and 8 review articles/book chapters. Her main research area is the effect of physical exercise in fibromyalgia, psychosocial factors, assessment of function, work capacity and quality of life.

Sjörs Anna, PhD.
Dr. Sjörs has a diploma as an ingenier in technical biology 2006. She defended her thesis 2010 at Linköpings University. She has published 4 scientific articles. Her main research areas are the autonomic nervous system and heart rate variability analysis.

PhD-students in alphabetic order
Forsberg Frida, MD, PhD-student, Linköpings University.
Ghafouri Nazdar, MD, PhD-student, Linköpings University.
Larsson Anette, physical therapist, University of Gothenburg
Palstam Annie, physical therapist, University of Gothenburg
Two PhD students will most likely be recruited to the project at Karolinska Institute, Stockholm.

Bil 3

Styrketräning och avspänning vid fibromyalgi

Vi söker kvinnor med fibromyalgi, i åldrarna 20-65 år, som är intresserade av att delta i en studie om smärtmekanismer vid styrketräning respektive avspänningsträning. Studien utförs som ett samarbetsprojekt mellan Göteborgs Universitet, Karolinska Institutet, Stockholm och Linköpings Universitet samt sjukvårdsenheter/kliniker i respektive region. I studien ingår ledarledd styrke- eller avspänningsträning 2 ggr i veckan under 15 veckor. I Västra Götalandslän genomförs studien i Göteborg och Alingsås och huvudansvarig är Doc. Kaisa Mannerkorpi, Sjukgymnastiken SU/Sahlgrenska.

För mer ingående och skriftlig information kontakta:

Docent Kaisa Mannerkorpi, SU/Sjukgymnastikenheten, tel 031-3421195, och Avd för Reumatologi och Inflammationsforskning, Sahlgrenska Akademin, Göteborgs Universitet, tel. 031-3424692. Email: kaisa.mannerkorpi@rheuma.gu.se

Kontaktperson i Alingsås: Anette Larsson, leg sjukgymnast, NärRehab Mitten Älvsborg, Herrljunga Vårdcentral, tel 0322-624076

Styrketräning och avspänning vid fibromyalgi

Vi söker kvinnor med fibromyalgi, i åldrarna 20-65 år, som är intresserade av att delta i en studie om smärtmekanismer vid styrketräning respektive avspänningsträning. Studien utförs som ett samarbetsprojekt mellan Göteborgs Universitet, Karolinska Institutet, Stockholm och Linköpings Universitet samt sjukvårdsenheter/kliniker i respektive region. I studien ingår ledarledd styrke- eller avspänningsträning 2 ggr i veckan under 15 veckor. I Linköping genomförs studien på Universitetssjukhuset och huvudansvarig för studien är Prof., Överläkare Björn Gerdle, Smärt och Rehabcentrum, Universitetssjukhuset, 581 85 Linköping.

För mer ingående och skriftlig information kontakta:

Docent, Överläkare Britt Larsson, Smärt och Rehabcentrum, Universitetssjukhuset, Linköping, e-mail: Britt.Larsson@inr.liu.se eller

Sekreterare Natalie Baldew, Smärt och Rehabcentrum, Universitetssjukhuset, Linköping, tfn: 010-1034453.

Styrketräning och avspänning vid fibromyalgi

Vi söker kvinnor med fibromyalgi, i åldrarna 20-65 år, som är intresserade av att delta i en studie om smärtmekanismer vid styrketräning respektive avspänningsträning. Studien utförs som ett samarbetsprojekt mellan Göteborgs Universitet, Karolinska Institutet, Stockholm och Linköpings Universitet samt sjukvårdsenheter/kliniker i respektive region. I studien ingår ledarledd styrke- eller avspänningsträning 2 ggr i veckan under 15 veckor. I Stockholm genomförs studien på Karolinska Institutet och Danderyds sjukhus och huvudansvarig för studien är Doc., Överläkare Eva Kosek, Institutionen för Klinisk Neurovetenskap, Karolinska Institutet, 171 77 Stockholm.

För mer ingående och skriftlig information kontakta:

Med.Dr. Leg sjukgymnast Monika Löfgren, Rehabilitetingsmedicinska Universitetskliniken, Danderyds Sjukhus AB, 182 88 Stockholm. E-mail: Monika.Lofgren@ds.se, Tfn: 08-655 78 13.

Med. Dr. Leg. läk Indre Ljungar, Rehabilitetingsmedicinska Universitetskliniken, Danderyds Sjukhus AB, 182 88 Stockholm. E-mail: Indre.Ljungar@ds.se

Friska kvinnor söks till studie om styrketräning

Vi söker friska kvinnor i åldrarna 20-65 år, som är intresserade att delta som kontrollpersoner i en studie om smärtmekanismer vid träning hos patienter med fibromyalgi. Studien utförs som ett samarbetsprojekt mellan Göteborgs Universitet, Karolinska Institutet, Stockholm och Linköpings Universitet samt sjukvårdsenheter/kliniker i respektive region. I studien ingår ledarledd styrketräning 2 ggr i veckan under 15 veckor. I Västra Götalandslän genomförs studien i Göteborg och Alingsås och huvudansvarig är Doc. Kaisa Mannerkorpi, Sjukgymnastiken SU/Sahlgrenska.

För mer ingående och skriftlig information kontakta:

Docent Kaisa Mannerkorpi, SU/Sjukgymnastikenheten, tel 031-3421195, och Avd för Reumatologi och Inflammationsforskning, Sahlgrenska Akademin, Göteborgs Universitet, tel. 031-3424692. Email: kaisa.mannerkorpi@rheuma.gu.se

Kontaktperson i Alingsås: Anette Larsson, leg sjukgymnast, NärRehab Mitten Älvsborg, Herrljunga Vårdcentral, tel 0322-624076

Friska kvinnor söks till studie om styrketräning

Vi söker friska kvinnor i åldrarna 20-65 år, som är intresserade att delta som kontrollpersoner i en studie om smärtmekanismer vid träning hos patienter med fibromyalgi. Studien utförs som ett samarbetsprojekt mellan Göteborgs Universitet, Karolinska Institutet, Stockholm och Linköpings Universitet samt sjukvårdsenheter/kliniker i respektive region. I studien ingår ledarledd styrketräning 2 ggr i veckan under 15 veckor. I Linköping genomförs studien på Universitetssjukhuset och huvudansvarig för studien är Prof., Överläkare Björn Gerdle, Smärt och Rehabcentrum, Universitetssjukhuset, 581 85 Linköping.

För mer ingående och skriftlig information kontakta:

Med. Dr. Leg sjukgymnast, Docent, Överläkare Britt Larsson, Smärt och Rehabcentrum, Universitetssjukhuset, Linköping, e-mail: Britt.Larsson@inr.liu.se eller

Sekreterare Natalie Baldew, Smärt och Rehabcentrum, Universitetssjukhuset, Linköping, tfn: 010-1034453.

Friska kvinnor söks till studie om styrketräning

Vi söker friska kvinnor i åldrarna 20-65 år, som är intresserade att delta som kontrollpersoner i en studie om smärtmekanismer vid träning hos patienter med fibromyalgi. Studien utförs som ett samarbetsprojekt mellan Göteborgs Universitet, Karolinska Institutet, Stockholm och Linköpings Universitet samt sjukvårdsenheter/kliniker i respektive region. I studien ingår ledarledd styrketräning 2 ggr i veckan under 15 veckor. I Stockholm genomförs studien på Karolinska Institutet och Danderyds sjukhus och huvudansvarig för studien är Doc., Överläkare Eva Kosek, Institutionen för Klinisk Neurovetenskap, Karolinska Institutet, 171 77 Stockholm.

För mer ingående och skriftlig information kontakta:

Med.Dr. Leg sjukgymnast Monika Löfgren, Rehabilitetingsmedicinska Universitetskliniken, Danderyds Sjukhus AB, 182 88 Stockholm. E-mail: Monika.Lofgren@ds.se, Tfn: 08-655 78 13.

Med. Dr. Leg. läk Indre Ljungar, Rehabilitetingsmedicinska Universitetskliniken, Danderyds Sjukhus AB, 182 88 Stockholm. E-mail: Indre.Ljungar@ds.se

Bil 4

Förfågan om deltagande i en studie om effekten av fysisk träning på sjukdomsmekanismer och symtom vid fibromyalgi .

Bakgrund och syfte. Det saknas förståelse för sjukdomsmekanismer vid fibromyalgi och därmed saknas även effektiv behandling. Många former av träning anses förbättra funktion och symtom hos personer med fibromyalgi, men mer kunskaper behövs om effekter av olika träningsformer likväl som om smärtmekanismer hos personer med fibromyalgi. Denna studie syftar till att öka förståelsen för smärtmekanismer hos patienter med fibromyalgi och undersöka effekterna av styrke- respektive avspänningsträning på sjukdomsmekanismer och hälsotillståndet. Eftersom man i tidigare forskning sett att vissa ärftliga (genetiska) faktorer har betydelse vid fibromyalgi kommer ärftliga faktorers inverkan på smärtmekanismer och effekten av träning att undersökas i denna studie. Effekterna av träning kommer att utvärderas avseende psykisk och fysisk hälsa, funktion och livskvalitet. Ökade kunskaper är nödvändiga för att på sikt kunna utveckla mer effektiv behandling. Studien utförs i Göteborg-Alingsås, Stockholm och Linköping.

Vem kan delta?
Vi söker kvinnor med fibromyalgi i åldrarna 20-65 år som är bosatta i eller närheten av Göteborg-Alingsås och som är intresserade av att delta i ledarledd träning antigen i form av styrketräning eller avspänningsträning två gånger i veckan under 15 veckor.

Hur går studien till?

Besök 1 (ca 1 timme)
Forskningsstudien inleds med en genomgång av din hälsa/dina sjukdomar, du får besvara ett frågeformulär om alkoholkonsumtion samt genomgå en läkarundersökning för fastställa/bekräfta diagnosen fibromyalgi. Du tillfrågas också om du inom ramen för denna studie kan tänka dig att delta i en mikrodialysundersökning av muskel. Studiedeltagare lottas därefter till någondera av två träningsgrupper: den ena gruppen ska delta i ledarledd styrketräning och den andra i ledarledd avspänningsträning.

Besök 2 (ca 2 timmar)
Vid andra besöket får du besvara olika frågeformulär avseende din smärta, sömn, ev. psykiska symtom, din fysiska aktivitet och din livskvalitet.
Smärtkänslighet för tryck undersöks med s.k. tryckalgometer. Därvid trycker försöksledaren med en stav mot olika delar av din kropp och du får signalera när trycket gör minsta lilla ont, resp. motsvarar en smärta som du skattar som måttlig eller mycket stark. Känsligheten för tryck undersöks i vila och under statiskt muskelarbete (du trycker underbenet mot ett fast motstånd).
Känsligheten för värme och kyla undersöks genom att en metallplatta läggs mot låret och du signalerar när du känner minsta lilla värme/kyla, när värmen/kylan gör minsta lilla ont och när du skulle skatta värmesmärtan som måttlig eller mycket stark, samt hur ont värmen gör när den ges under 2 minuter. Du kan när som helst avbryta stimuleringen.
Vid besöket får Du också genomgå mätningar av din styrka och balans samt utföra ett 6 minuters gångtest.
Du ombeds att uppsöka Reumatologen/SU eller ett näraliggande sjukhus för blodprovstagning (utom för de försökspersoner som deltar i microdialysundersökningen, där blodprov tas i samband med denna). Blodprovet (20 ml) kommer att användas för analys av ämnen som är

av betydelse för reglering av smärta och inflammation. Du får lämna ett salivprov för analys av vissa gener som har betydelse för smärtreglering.
Slutligen kommer hälften av deltagarna att få registrera EKG under 24 timmar med en liten bärbar EKG-bandspelare. Under EKG registreringen ska du leva som vanligt förutom att du inte kan duscha och bada. Efter 24-timmar kan du ta bort elektroderna och lämna tillbaka apparaten.

De som lottats till avspänningsträning:
De som lottats till avspänningsträning påbörjar nu denna, 2 gånger i veckan under 15 veckor i grupper av 10. Träningen leds av speciellt utbildad sjukgymnast. De första träningspassen tar ca 2 timmar därefter tar träningspassen ca 1 timme i anspråk. Efter avslutad avspänningsträning upprepas alla mätningar (se besök 2, ovan).
Ett år senare kommer vi att skicka hem samma frågeformulär som besvaras och skickas tillbaka till oss i frankerade kuvert som vi tillhandahåller.

De som lottats till styrketräning:
Besök 3 (ca 6 timmar)
Detta besök gäller endast de försökspersoner som sagt sig vara villiga att delta i mikrodialysundersökningen.
Mikrodialys
Huden mitt på låret bedövas med bedövningsmedel. Därefter förs två mycket tunna mikrodialyskatetrar (trådtunna slangar) in i lårmuskeln. En vätska cirkulerar långsamt med hjälp av en lite pump och prover tas med regelbundna intervall från vätskan för analys av ämnesomsättningsämnen samt smärt- och inflammationsframkallande substanser. Du kommer även att få utföra ett statiskt muskelarbete med lårmuskeln. Sammantaget tar själva mikrodialysundersökningen 4 timmar och 20 minuter i anspråk och katetrarna avlägsnas. Efter avslutad mikrodialysundersökning sker ny bedövning av ett litet område av huden, och en 4-6 mm stort vävnadsprov tas från huden.

De som lottats till styrketräning påbörjar nu denna, 2 gånger i veckan under 15 veckor i grupper av 5. Träningen leds av speciellt utbildad sjukgymnast och varje pass. De första träningspassen tar ca 2 timmar därefter tar träningspassen ca 1 timme i anspråk. Efter avslutad styrketräning upprepas alla mätningar (besök 2, 4 och 5 samt 3 för de som deltar i microdialysdelen).
Ett år senare kommer vi att skicka hem samma frågeformulär som besvaras och skickas tillbaka till oss i frankerade kuvert som vi tillhandahåller.

Blodprovet lagras tills vidare i en så kallad biobank vid varje ort. Salivprovet lagras på Karolinska Institutets Biobank i Stockholm. Proverna förvaras kodade, vilket innebär att de inte kan härledas till dig som person, och de kommer att används endast på det sätt som du givit samtycke till. De kan endast bli aktuella för ett nytt forskningsprojekt efter att du blivit tillfrågad och lämnat ett nytt samtycke och/eller godkännande skett av etikprövningsnämnden (Biobankslagen, SFS 2002:297).

Vilka är riskerna?
Vid undersökning av smärtkänslighet utsätts du för kortvarig smärta som du när som helst kan avbryta. Smärtstimuleringarna avbryts alltid vid ett visst tryck (1500 kPa) och håller sig inom temperaturintervallet (0 – 50°C) för att undvika vävnadsskada. Blodprovstagning följer sedvanliga kliniska rutiner och är inte förenat med allvarliga risker. Registrering av 24-timmars EKG innebär inga risker men innebär att du inte kan duscha och bada under

registreringen och kan upplevas som lite obekvämt nattetid. Mikrodialys och hudbiopsi utförs under bedövning med lokalt bedövningsmedel och är inte förenat med smärta. När slangarna är på plats i muskulaturen känns de inte och det känns inte heller när de avlägsnas. En liten risk för infektion eller blödning finns och om tecken på detta skulle uppstå behandlas den på sedvanligt sätt (du kommer att få uppgift om vem du ska kontakta i så fall). Noggrann hygien iakttas för att undvika infektioner. För att kunna bestämma nivåerna av de biokemiska ämnena används en mycket liten dos radioaktiva ämnen i den vätska som genomsköljer de slangar som lagts in i muskulaturen. Strålningen av de radioaktiva ämnena motsvarar 25 % av strålningen vid t ex en röntgenundersökning av en arm. Denna dos är inte förknippad med någon märkbart ökad risk för cancer. Träningsrelaterad smärta kan förekomma vid styrketräning, men även den är övergående. Undersökningsmetoder och träningsprogrammet är beprövade och leder inte till några bestående bieffekter eller vävnadsskada.

Finns det några fördelar?
Du kommer att få en uppfattning om din fysiska funktion och ditt hälsotillstånd. Vi kommer att få kunskap om effekter av två olika former av träning vid fibromyalgi, samt om träningens påverkan på smärta och funktion.

Hantering av data och sekretess
Alla försökspersoner erhåller ett studiespecifikt nummer. All information om dig registreras i en speciell mapp märkt med ditt nummer och all databearbetning sker med avidentifierade data. Alla frågeformulär och ditt försöksprotokoll marks endast med dina initialer och ditt studienummer och alltså inte med ditt fullständiga namn. Dina personuppgifter och ditt kodnummer förvaras separat inlåst i journalskåp. Dina personuppgifter skyddas av den sekretess som anges i journallagen. Originaldata sparas under minst 10 år för att möjliggöra granskning av projektets kvalité. Studiens resultat kommer att presenteras i en vetenskaplig tidskrift på gruppnivå, vilket innebär att det inte går att röja enskild individ.

Hur får jag information om studiens resultat?
Efter att data har analyserats kommer vi att presentera studiens resultat i vetenskapliga artiklar och på olika seminarier och föreläsningar.

Försäkring och ersättning
Försökspersoner som lottas till styrketräning får tillgång till träningslokal (alt. gymkort i 6 månader) och erhåller 500kr efter studiens slut. Personer som lottas till avspänningsträning erhåller 500 kr efter studiens avslut. De försökspersoner som deltar i mikrodialys/hudbiopsi får 3 000 kr efter sista undersökningstillfället dvs. efter mikrodialysundersökning/hudbiopsi för andra gången efter avslutad styrketräning. Ersättningen är skattepliktig. Sjukvårdens patientskadeförsäkring gäller för undersökningarna.

Frivillighet
Det är givetvis frivilligt att delta i forskningsprojektet, och Du kan när som helst avbryta Ditt deltagande om Du så önskar utan att ange någon orsak. Frågeformulär och blodprover märks så att det inte är möjligt att spåra dem till den enskilde, och du har rätt att begära att de förstörs. Vill du inte delta, eller vill du avbryta studien kommer detta inte att påverka din sedvanliga behandling.

Ansvarig för studien i Göteborg-Alingsås

Docent Kaisa Mannerkorpi, SU/Sjukgymnastikenheten, tel 031-3421195, och Avd för Reumatologi och Inflammationsforskning, Sahlgrenska Akademin, Göteborgs Universitet, tel. 031-3424692. Email: kaisa.mannerkorpi@rheuma.gu.se

För mer information om studien kontakta:

Docent Kaisa Mannerkorpi, SU/Sjukgymnastikenheten, tel 031-3421195, och Avd för Reumatologi och Inflammationsforskning, Sahlgrenska Akademin, Göteborgs Universitet, tel. 031-3424692. Email: kaisa.mannerkorpi@rheuma.gu.se

Avseende läkarundersökning: Annelie Lund, forskningssjuksköterska, Forsknings- och utvecklingsenheten, Reumatologi, Sahlgrenska Universitetssjukhuset, Gröna Stråket 14 plan 5, 413 45 Göteborg, Tel: 031-342 8639

Kontaktperson i Alingsås: Anette Larsson, leg sjukgymnast, NärRehab Mitten Älvsborg, Herrljunga Vårdcentral, tel 0322-624076

Informerat samtycke till att delta i studien
" Effekten av fysisk träning på sjukdomsmekanismer och symtom vid fibromyalgi."

Jag har både skriftligt och muntligt blivit informerad om studien och den databehandling av personuppgifter som studien innebär. Jag har även fått tillfälle att ställa frågor och fått en kopia av patientinformationen.

Jag är medveten om att mitt deltagande i studien är fullt frivilligt och att jag när som helst och utan närmare förklaring kan avbryta mitt deltagande utan att detta påverkar min nuvarande eller kommande behandling.

Alternativ 1: Jag samtycker till att delta i detta forskningsprojekt i sin helhet (dvs. även microdialys/hudbiopsi).

Ort och datum ____________________

____
Patientens namnteckning

____
Namnförtydligande

Alternativ 2: Jag samtycker till att delta i detta forskningsprojekt men inte i microdialys/hudbiopsidelen.

Ort och datum ____________________

____
Patientens namnteckning

____
Namnförtydligande

Jag bekräftar att patienten har fått information om forskningsprojektet och intygar att patienten själv har undertecknat och daterat samtycket.

Ort och datum ______________________

____
Ansvarig sjukgymnast

____
Namnförtydligande

En kopia av denna signerade patientinformation ges till patienten.

Förfågan om deltagande i en studie om effekten av fysisk träning på sjukdomsmekanismer och symtom vid fibromyalgi .

Bakgrund och syfte. Det saknas förståelse för sjukdomsmekanismer vid fibromyalgi och därmed saknas även effektiv behandling. Många former av träning anses förbättra funktion och symtom hos personer med fibromyalgi, men mer kunskaper behövs om effekter av olika träningsformer likväl som om smärtmekanismer hos personer med fibromyalgi. Denna studie syftar till att öka förståelsen för smärtmekanismer hos patienter med fibromyalgi och undersöka effekterna av styrke- respektive avspänningsträning på sjukdomsmekanismer och hälsotillståndet. Eftersom man i tidigare forskning sett att vissa ärftliga (genetiska) faktorer har betydelse vid fibromyalgi kommer ärftliga faktorers inverkan på smärtmekanismer och effekten av träning att undersökas i denna studie. Effekterna av träning kommer att utvärderas avseende psykisk och fysisk hälsa, funktion och livskvalitet. Ökade kunskaper är nödvändiga för att på sikt kunna utveckla mer effektiv behandling. Studien utförs i Göteborg-Alingsås, Stockholm och Linköping.

Vem kan delta?
Vi söker kvinnor med fibromyalgi i åldrarna 20-65 år som är bosatta i eller närheten av Linköping och som är intresserade av att delta i ledarledd träning antigen i form av styrketräning eller avspänningsträning två gånger i veckan under 15 veckor.

Hur går studien till?

Besök 1 (ca 1 timme)
Forskningsstudien inleds med en genomgång av din hälsa/dina sjukdomar, du får besvara ett frågeformulär om alkoholkonsumtion samt genomgå en läkarundersökning för fastställa/bekräfta diagnosen fibromyalgi. Du tillfrågas också om du inom ramen för denna studie kan tänka dig att delta i en mikrodialysundersökning av muskel. Studiedeltagare lottas därefter till någondera av två träningsgrupper: den ena gruppen ska delta i ledarledd styrketräning och den andra i ledarledd avspänningsträning.

Besök 2 (ca 2 timmar)
Vid andra besöket får du besvara olika frågeformulär avseende din smärta, sömn, ev. psykiska symtom, din fysiska aktivitet och din livskvalitet.
Smärtkänslighet för tryck undersöks med s.k. tryckalgometer. Därvid trycker försöksledaren med en stav mot olika delar av din kropp och du får signalera när trycket gör minsta lilla ont, resp. motsvarar en smärta som du skattar som måttlig eller mycket stark. Känsligheten för tryck undersöks i vila och under statiskt muskelarbete (du trycker underbenet mot ett fast motstånd).
Känsligheten för värme och kyla undersöks genom att en metallplatta läggs mot låret och du signalerar när du känner minsta lilla värme/kyla, när värmen/kylan gör minsta lilla ont och när du skulle skatta värmesmärtan som måttlig eller mycket stark, samt hur ont värmen gör när den ges under 2 minuter. Du kan när som helst avbryta stimuleringen.
Vid besöket får Du också genomgå mätningar av din styrka och balans samt utföra ett 6 minuters gångtest.
Vi tar ett blodprov (20 ml)(utom för de försökspersoner som deltar i mikrodialys, där blodprov tas i samband med denna). Blodprovet kommer att användas för analys av ämnen som är av betydelse för reglering av smärta och inflammation. Salivprov tas för analys av vissa gener som har betydelse för smärtreglering.

Slutligen kommer hälften av deltagarna att få registrera EKG under 24 timmar med en liten bärbar EKG-bandspelare. Under EKG registreringen ska du leva som vanligt förutom att du inte kan duscha och bada. Efter 24-timmar kan du ta bort elektroderna och lämna tillbaka apparaten.

De som lottats till avspänningsträning:
De som lottats till avspänningsträning påbörjar nu denna, 2 gånger i veckan under 15 veckor i grupper av 10. Träningen leds av speciellt utbildad sjukgymnast. De första träningspassen tar ca 1,5 timme därefter tar träningspassen ca 1 timme i anspråk. Efter avslutad avspänningsträning upprepas alla mätningar (se besök 2, ovan).
Ett år senare kommer vi att skicka hem samma frågeformulär som besvaras och skickas tillbaka till oss i frankerade kuvert som vi tillhandahåller.

De som lottats till styrketräning:
Besök 3 (ca 6 timmar)
Detta besök gäller endast de försökspersoner som sagt sig vara villiga att delta i microdialysundersökningen.
Mikrodialys
Huden mitt på låret bedövas med bedövningsmedel. Därefter förs två mycket tunna mikrodialyskatetrar (trådtunna slangar) in i lårmuskeln. En vätska cirkulerar långsamt med hjälp av en lite pump och prover tas med regelbundna intervall från vätskan för analys av ämnesomsättningsämnen samt smärt- och inflammationsframkallande substanser. Du kommer även att få utföra ett statiskt muskelarbete med lårmuskeln. Sammantaget tar själva mikrodialysundersökningen 4 timmar och 20 minuter i anspråk och katetrarna avlägsnas. Efter avslutad mikrodialysundersökning sker ny bedövning av ett litet område av huden, och en 4-6 mm stort vävnadsprov tas från huden.

De som lottats till styrketräning påbörjar nu denna, 2 gånger i veckan under 15 veckor i grupper av 5. Träningen leds av speciellt utbildad sjukgymnast och varje pass. De första träningspassen tar ca 2 timmar därefter tar träningspassen ca 1 timme i anspråk. Efter avslutad styrketräning upprepas alla mätningar (besök 2, 4 och 5 samt 3 för de som deltar i microdialysdelen).
Ett år senare kommer vi att skicka hem samma frågeformulär som besvaras och skickas tillbaka till oss i frankerade kuvert som vi tillhandahåller.

Blodprovet lagras tills vidare i en så kallad biobank vid varje ort. Salivprovet förvaras på Karolinska Institutets Biobank i Stockholm. Proverna förvaras kodade, vilket innebär att de inte kan härledas till dig som person, och de kommer att används endast på det sätt som du givit samtycke till. De kan endast bli aktuella för ett nytt forskningsprojekt efter att du blivit tillfrågad och lämnat ett nytt samtycke och/eller godkännande skett av etikprövningsnämnden (Biobankslagen, SFS 2002:297).

Vilka är riskerna?
Vid undersökning av smärtkänslighet utsätts du för kortvarig smärta som du när som helst kan avbryta. Smärtstimuleringarna avbryts alltid vid ett visst tryck (1500 kPa) och håller sig inom temperaturintervallet (0 – 50°C) för att undvika vävnadsskada. Blodprovstagning följer sedvanliga kliniska rutiner och är inte förenat med allvarliga risker. Registrering av 24-timmars EKG innebär inga risker men innebär att du inte kan duscha och bada under registreringen och kan upplevas som lite obekvämt nattetid. Mikrodialys och hudbiopsi utförs under bedövning med lokalt bedövningsmedel och är inte förenat med smärta. När slangarna

är på plats i muskulaturen känns de inte och det känns inte heller när de avlägsnas. En liten risk för infektion eller blödning finns och om tecken på detta skulle uppstå behandlas den på sedvanligt sätt (du kommer att få uppgift om vem du ska kontakta i så fall). Noggrann hygien iakttas för att undvika infektioner. För att kunna bestämma nivåerna av de biokemiska ämnena används en mycket liten dos radioaktiva ämnen i den vätska som genomsköljer de slangar som lagts in i muskulaturen. Strålningen av de radioaktiva ämnena motsvarar 25 % av strålningen vid t ex en röntgenundersökning av en arm. Denna dos är inte förknippad med någon märkbart ökad risk för cancer. Träningsrelaterad smärta kan förekomma vid styrketräning, men även den är övergående. Undersökningsmetoder och träningsprogrammet är beprövade och leder inte till några bestående bieffekter eller vävnadsskada.

Finns det några fördelar?
Du kommer att få en uppfattning om din fysiska funktion och ditt hälsotillstånd. Vi kommer att få kunskap om effekter av två olika former av träning vid fibromyalgi, samt om träningens påverkan på smärta och funktion.

Hantering av data och sekretess
Alla försökspersoner erhåller ett studiespecifikt nummer. All information om dig registreras i en speciell mapp märkt med ditt nummer och all databearbetning sker med avidentifierade data. Alla frågeformulär och ditt försöksprotokoll marks endast med dina initialer och ditt studienummer och alltså inte med ditt fullständiga namn. Dina personuppgifter och ditt kodnummer förvaras separat inlåst i journalskåp. Dina personuppgifter skyddas av den sekretess som anges i journallagen. Originaldata sparas under minst 10 år för att möjliggöra granskning av projektets kvalité. Studiens resultat kommer att presenteras i en vetenskaplig tidskrift på gruppnivå, vilket innebär att det inte går att röja enskild individ.

Hur får jag information om studiens resultat?
Efter att data har analyserats kommer vi att presentera studiens resultat i vetenskapliga artiklar och på olika seminarier och föreläsningar.

Försäkring och ersättning
Försökspersoner som lottas till styrketräning får tillgång till träningslokal (alt. gymkort i 6 månader) och erhåller 500kr efter studiens slut. Personer som lottas till avspänningsträning erhåller 500 kr efter studiens slut. De försökspersoner som deltar i mikrodialys/hudbiopsi får 3 000 kr efter sista undersökningstillfället dvs. efter mikrodialysundersökning/hudbiopsi för andra gången efter avslutad styrketräning. Ersättningen är skattepliktig. Sjukvårdens patientskadeförsäkring gäller för undersökningarna.

Frivillighet
Det är givetvis frivilligt att delta i forskningsprojektet, och Du kan när som helst avbryta Ditt deltagande om Du så önskar utan att ange någon orsak. Frågeformulär och blodprover märks så att det inte är möjligt att spåra dem till den enskilde, och du har rätt att begära att de förstörs. Vill du inte delta, eller vill du avbryta studien kommer detta inte att påverka din sedvanliga behandling.

Ansvarig för studien i Linköping

Prof., Överläkare Björn Gerdle, Smärt och Rehabcentrum, Universitetssjukhuset, 581 85 Linköping.

För mer information om studien kontakta:

Docent, Överläkare Britt Larsson, Smärt och Rehabcentrum, Universitetssjukhuset, Linköping, e-mail: Britt.Larsson@inr.liu.se eller

Sekreterare Natalie Baldew, Smärt och Rehabcentrum, Universitetssjukhuset, Linköping, tfn: 010-1034453.

Informerat samtycke till att delta i studien
" Effekten av fysisk träning på sjukdomsmekanismer och symtom vid fibromyalgi."

Jag har både skriftligt och muntligt blivit informerad om studien och den databehandling av personuppgifter som studien innebär. Jag har även fått tillfälle att ställa frågor och fått en kopia av patientinformationen.

Jag är medveten om att mitt deltagande i studien är fullt frivilligt och att jag när som helst och utan närmare förklaring kan avbryta mitt deltagande utan att detta påverkar min nuvarande eller kommande behandling.

Alternativ 1: Jag samtycker till att delta i detta forskningsprojekt i sin helhet (dvs. även microdialys/hudbiopsi).

Ort och datum ____________________

____
Patientens namnteckning

____
Namnförtydligande

Alternativ 2: Jag samtycker till att delta i detta forskningsprojekt men inte i microdialys/hudbiopsidelen.

Ort och datum ____________________

____
Patientens namnteckning

____
Namnförtydligande

Jag bekräftar att patienten har fått information om forskningsprojektet och intygar att patienten själv har undertecknat och daterat samtycket.

Ort och datum ______________________________

____
Ansvarig sjukgymnast

____
Namnförtydligande

En kopia av denna signerade patientinformation ges till patienten.

Förfågan om deltagande i en studie om effekten av fysisk träning på sjukdomsmekanismer och symtom vid fibromyalgi .

Bakgrund och syfte. Det saknas förståelse för sjukdomsmekanismer vid fibromyalgi och därmed saknas även effektiv behandling. Många former av träning anses förbättra funktion och symtom hos personer med fibromyalgi, men mer kunskaper behövs om effekter av olika träningsformer likväl som om smärtmekanismer hos personer med fibromyalgi. Denna studie syftar till att öka förståelsen för smärtmekanismer hos patienter med fibromyalgi och undersöka effekterna av styrke- respektive avspänningsträning på sjukdomsmekanismer och hälsotillståndet. Eftersom man i tidigare forskning sett att vissa ärftliga (genetiska) faktorer har betydelse vid fibromyalgi kommer ärftliga faktorers inverkan på smärtmekanismer och effekten av träning att undersökas i denna studie. Effekterna av träning kommer att utvärderas avseende psykisk och fysisk hälsa, funktion och livskvalitet. Ökade kunskaper är nödvändiga för att på sikt kunna utveckla mer effektiv behandling. Studien utförs i Göteborg-Alingsås, Stockholm och Linköping.

Vem kan delta?
Vi söker kvinnor med fibromyalgi i åldrarna 20-65 år som är bosatta i eller närheten av Stockholm och som är intresserade av att delta i ledarledd träning antigen i form av styrketräning eller avspänningsträning två gånger i veckan under 15 veckor.

Hur går studien till?

Besök 1 (ca 1 timme)
Forskningsstudien inleds med en genomgång av din hälsa/dina sjukdomar, du får besvara ett frågeformulär om alkoholkonsumtion samt genomgå en läkarundersökning för fastställa/bekräfta diagnosen fibromyalgi. Du tillfrågas också om du inom ramen för denna studie kan tänka dig att delta i en mikrodialysundersökning av muskel. Studiedeltagare lottas därefter till någondera av två träningsgrupper: den ena gruppen ska delta i ledarledd styrketräning och den andra i ledarledd avspänningsträning.

Besök 2 (ca 2 timmar)
Vid andra besöket får du besvara olika frågeformulär avseende din smärta, sömn, ev. psykiska symtom, din fysiska aktivitet och din livskvalitet.
Smärtkänslighet för tryck undersöks med s.k. tryckalgometer. Därvid trycker försöksledaren med en stav mot olika delar av din kropp och du får signalera när trycket gör minsta lilla ont, resp. motsvarar en smärta som du skattar som måttlig eller mycket stark. Känsligheten för tryck undersöks i vila och under statiskt muskelarbete (du trycker underbenet mot ett fast motstånd).
Känsligheten för värme och kyla undersöks genom att en metallplatta läggs mot låret och du signalerar när du känner minsta lilla värme/kyla, när värmen/kylan gör minsta lilla ont och när du skulle skatta värmesmärtan som måttlig eller mycket stark, samt hur ont värmen gör när den ges under 2 minuter. Du kan när som helst avbryta stimuleringen.
Vid besöket får Du också genomgå mätningar av din styrka och balans samt utföra ett 6 minuters gångtest.
Vi tar ett blodprov (20 ml)(utom för de försökspersoner som deltar i mikrodialys, där blodprov tas i samband med denna). Blodprovet kommer att användas för analys av ämnen som är av betydelse för reglering av smärta och inflammation. Salivprov tas för analys av vissa gener som har betydelse för smärtreglering.

Slutligen kommer hälften av deltagarna att få registrera EKG under 24 timmar med en liten bärbar EKG-bandspelare. Under EKG registreringen ska du leva som vanligt förutom att du inte kan duscha och bada. Efter 24-timmar kan du ta bort elektroderna och lämna tillbaka apparaten.

De som lottats till avspänningsträning:
De som lottats till avspänningsträning påbörjar nu denna, 2 gånger i veckan under 15 veckor i grupper av 10. Träningen leds av speciellt utbildad sjukgymnast. De första träningspassen tar ca 2 timmar därefter tar träningspassen ca 1 timme i anspråk. Efter avslutad avspänningsträning upprepas alla mätningar (se besök 2, ovan).
Ett år senare kommer vi att skicka hem samma frågeformulär som besvaras och skickas tillbaka till oss i frankerade kuvert som vi tillhandahåller.

De som lottats till styrketräning:
Besök 3 (ca 6 timmar)
Detta besök gäller endast de försökspersoner som sagt sig vara villiga att delta i microdialysundersökningen.
Mikrodialys
Huden mitt på låret bedövas med bedövningsmedel. Därefter förs två mycket tunna mikrodialyskatetrar (trådtunna slangar) in i lårmuskeln. En vätska cirkulerar långsamt med hjälp av en lite pump och prover tas med regelbundna intervall från vätskan för analys av ämnesomsättningsämnen samt smärt- och inflammationsframkallande substanser. Du kommer även att få utföra ett statiskt muskelarbete med lårmuskeln. Sammantaget tar själva mikrodialysundersökningen 4 timmar och 20 minuter i anspråk och katetrarna avlägsnas. Efter avslutad mikrodialysundersökning sker ny bedövning av ett litet område av huden, och en 4-6 mm stort vävnadsprov tas från huden.

Hjärnavbildningsundersökning
Innan träningen påbörjas kommer alla som lottats till styrketräning att kallas till en hjärnavbildningsundersökning med funktionell magnetisk resonans (fMRI). Magnetresonans (MR) har revolutionerat möjligheter att återge en detaljerad bild av hjärnan och dess funktion, och används idag för att diagnostisera många olika sjukdomar. Tekniken har använts för att studera hjärnans funktion i många smärttillstånd, så som fibromyalgi.

Besök 4 (ca 1 timme)
Vid det fjärde besöket undersöker vi din smärtkänslighet för tryck mot tumnagel för att fastställa vilket tryck som ska användas vid fMRI undersökningen.

Besök 5 (ca 1 timme)
Du kommer att få ligga i en magnetröntgenkamera och samtidigt utföres tryck mot din tumnageln.

De som lottats till styrketräning påbörjar nu denna, 2 gånger i veckan under 15 veckor i grupper av 5. Träningen leds av speciellt utbildad sjukgymnast och varje pass. De första träningspassen tar ca 2 timmar därefter tar träningspassen ca 1 timme i anspråk. Efter avslutad styrketräning upprepas alla mätningar (besök 2, 4 och 5 samt 3 för de som deltar i microdialysdelen).
Ett år senare kommer vi att skicka hem samma frågeformulär som besvaras och skickas tillbaka till oss i frankerade kuvert som vi tillhandahåller.

Blodprovet lagras tills vidare i en så kallad biobank vid varje ort. Salivprovet lagras på Karolinska Institutets Biobank, Stockholm. Proverna förvaras kodade, vilket innebär att de inte kan härledas till dig som person, och de kommer att används endast på det sätt som du givit samtycke till. De kan endast bli aktuella för ett nytt forskningsprojekt efter att du blivit tillfrågad och lämnat ett nytt samtycke och/eller godkännande skett av etikprövningsnämnden (Biobankslagen, SFS 2002:297).

Vilka är riskerna?
Vid undersökning av smärtkänslighet utsätts du för kortvarig smärta som du när som helst kan avbryta. Smärtstimuleringarna avbryts alltid vid ett visst tryck (1500 kPa) och håller sig inom temperaturintervallet (0 – 50°C) för att undvika vävnadsskada. Blodprovstagning följer sedvanliga kliniska rutiner och är inte förenat med allvarliga risker. Registrering av 24-timmars EKG innebär inga risker men innebär att du inte kan duscha och bada under registreringen och kan upplevas som lite obekvämt nattetid. Mikrodialys och hudbiopsi utförs under bedövning med lokalt bedövningsmedel och är inte förenat med smärta. När slangarna är på plats i muskulaturen känns de inte och det känns inte heller när de avlägsnas. En liten risk för infektion eller blödning finns och om tecken på detta skulle uppstå behandlas den på sedvanligt sätt (du kommer att få uppgift om vem du ska kontakta i så fall). Noggrann hygien iakttas för att undvika infektioner. För att kunna bestämma nivåerna av de biokemiska ämnena används en mycket liten dos radioaktiva ämnen i den vätska som genomsköljer de slangar som lagts in i muskulaturen. Strålningen av de radioaktiva ämnena motsvarar 25 % av strålningen vid t ex en röntgenundersökning av en arm. Denna dos är inte förknippad med någon märkbart ökad risk för cancer. fMRI undersökningen är ofarlig men kan upplevas som skrämmande av människor med tendens till klaustrofobi. Träningsrelaterad smärta kan förekomma vid styrketräning, men även den är övergående. Undersökningsmetoder och träningsprogrammet är beprövade och leder inte till några bestående bieffekter eller vävnadsskada.

Finns det några fördelar?
Du kommer att få en uppfattning om din fysiska funktion och ditt hälsotillstånd. Vi kommer att få kunskap om effekter av två olika former av träning vid fibromyalgi, samt om träningens påverkan på smärta och funktion.

Hantering av data och sekretess
Alla försökspersoner erhåller ett studiespecifikt nummer. All information om dig registreras i en speciell mapp märkt med ditt nummer och all databearbetning sker med avidentifierade data. Alla frågeformulär och ditt försöksprotokoll marks endast med dina initialer och ditt studienummer och alltså inte med ditt fullständiga namn. Dina personuppgifter och ditt kodnummer förvaras separat inlåst i journalskåp. Dina personuppgifter skyddas av den sekretess som anges i journallagen. Originaldata sparas under minst 10 år för att möjliggöra granskning av projektets kvalité. Studiens resultat kommer att presenteras i en vetenskaplig tidskrift på gruppnivå, vilket innebär att det inte går att röja enskild individ.

Hur får jag information om studiens resultat?
Efter att data har analyserats kommer vi att presentera studiens resultat i vetenskapliga artiklar och på olika seminarier och föreläsningar.

Försäkring och ersättning
Personer som lottas till avspänningsträning erhåller 500 kr efter studiens slut. Försökspersoner som lottas till styrketräning får tillgång till träningslokal (alt. gymkort i 6 månader) och

erhåller 1000 kr efter studiens slut (pga fMRI undersökning tillkommer). De försökspersoner som även deltar i mikrodialys/hudbiopsi får totalt 3 500 kr efter sista undersökningstillfället dvs. efter mikrodialysundersökning/hudbiopsi och fMRI för andra gången efter avslutad styrketräning. Ersättningen är skattepliktig. Sjukvårdens patientskadeförsäkring gäller för undersökningarna.

Frivillighet

Det är givetvis frivilligt att delta i forskningsprojektet, och Du kan när som helst avbryta Ditt deltagande om Du så önskar utan att ange någon orsak. Frågeformulär och blodprover märks så att det inte är möjligt att spåra dem till den enskilde, och du har rätt att begära att de förstörs. Vill du inte delta, eller vill du avbryta studien kommer detta inte att påverka din sedvanliga behandling.

Ansvarig för studien i Stockholm

Docent Eva Kosek, Institutionen för Klinisk Neurovetenskap, Karolinska Institutet, Retziusväg 8, 171 77 Stockholm.

För mer information om studien kontakta:

Med.Dr. Monika Lövgren, Rehabiliteringsmedicinska Universitetskliniken, Danderyds sjukhus AB, 182 88, Stockholm. E-mail: Monika.Lofgren@ds.se, Tfn: 08-655 78 13.

Med. Dr. Leg.Läk. Indre Ljungar, Rehabiliteringsmedicinska Universitetskliniken, Danderyds sjukhus AB, 182 88, Stockholm. E-mail: Indre.Ljungar@ds.se.

Informerat samtycke till att delta i studien
" Effekten av fysisk träning på sjukdomsmekanismer och symtom vid fibromyalgi."

Jag har både skriftligt och muntligt blivit informerad om studien och den databehandling av personuppgifter som studien innebär. Jag har även fått tillfälle att ställa frågor och fått en kopia av patientinformationen.

Jag är medveten om att mitt deltagande i studien är fullt frivilligt och att jag när som helst och utan närmare förklaring kan avbryta mitt deltagande utan att detta påverkar min nuvarande eller kommande behandling.

Alternativ 1: Jag samtycker till att delta i detta forskningsprojekt i sin helhet (dvs. även microdialys/hudbiopsi).

Ort och datum ______________________

____
Patientens namnteckning

____
Namnförtydligande

Alternativ 2: Jag samtycker till att delta i detta forskningsprojekt men inte i microdialys/hudbiopsidelen.

Ort och datum ______________________

____
Patientens namnteckning

____
Namnförtydligande

Jag bekräftar att patienten har fått information om forskningsprojektet och intygar att patienten själv har undertecknat och daterat samtycket.

Ort och datum ______________________

____
Ansvarig sjukgymnast

____
Namnförtydligande

En kopia av denna signerade patientinformation ges till patienten.

Förfågan till friska kvinnor om deltagande som kontrollpersoner i en studie om effekten av fysisk träning på sjukdomsmekanismer och symtom vid smärtsyndromet fibromyalgi .

Bakgrund och syfte.
Vi söker friska kvinnor som kontrollpersoner i en studie av olika typer av träning för kvinnor med långvarig smärta orsakad av fibromyalgi. Det saknas förståelse för sjukdomsmekanismer vid detta smärttillstånd och därmed saknas även effektiv behandling. Många former av träning förbättrar funktion och symtom hos personer med fibromyalgi, men mer kunskaper behövs om effekterna av olika träningsformer likväl som om smärtmekanismer hos personer med fibromyalgi och friska.
Denna studie syftar till att öka förståelsen av smärtmekanismerna hos patienter med fibromyalgi och undersöka effekterna av styrke- respektive avspänningsträning på sjukdomsmekanismer och hälsotillstånd. Eftersom man i tidigare forskning sett att vissa ärftliga (genetiska) faktorer har betydelse vid fibromyalgi kommer ärftliga faktorers inverkan på smärtmekanismer och effekten av träning att undersökas i denna studie. Ökade kunskaper är nödvändiga för att på sikt kunna utveckla mer effektiv behandling.
Vi söker nu således *friska kvinnor utan smärta* som kan utgöra ett jämförelsematerial till våra patienter.

Vem kan delta?
Vi söker friska kvinnor i åldrarna 20-65 år utan smärta som är bosatta i eller närheten av Göteborg-Alingsås och som är intresserade av att delta som kontrollpersoner i en undersökning av smärtmekanismer och effekter av träning, vilket beskrivs mer i detalj nedan. Undersökningen består av 2 delar och man kan delta antigen bara i del 1 eller i både del 1 och del 2.

Hur går studien till?

Del 1
Besök 1 (ca 2,5 timmar): Besöket inleds med genomgång av din hälsa. Du får besvara olika frågeformulär avseende din smärta, sömn, ev. psykiska symtom, din fysiska aktivitet och din livskvalitet. Smärtkänslighet för tryck undersöks med s.k. tryckalgometer. Därvid trycker försöksledaren med en stav mot olika delar av din kropp och du får signalera när trycket gör minsta lilla ont, resp. motsvarar en smärta som du skattar som måttligt stark eller mycket stark. Känsligheten för tryck undersöks i vila och under statiskt muskelarbete (du trycker underbenet mot ett fast motstånd).
Känsligheten för värme och kyla undersöks genom att en metallplatta läggs mot låret och du signalerar när du känner minsta lilla värme/kyla, när värmen/kylan gör minsta lilla ont och när du skulle skatta värmesmärtan som måttligt stark eller mycket stark, samt hur ont värmen gör när den ges under 2 minuter. Du kan när som helst avbryta stimuleringen.
Du får genomgå mätningar av din styrka och balans samt utföra ett 6 minuters gångtest.
Du ombeds att uppsöka Reumatologen/SU eller ett näraliggande sjukhus för blodprovstagning (utom för de försökspersoner som deltar i microdialysundersökningen (del 2), där blodprov tas i samband med denna). Blodprovet (20 ml) kommer att användas för analys av ämnen som är av betydelse för reglering av smärta och inflammation. Salivprov tas för analys av vissa gener som har betydelse för smärtreglering.

De som även kommer att delta i del 2 får registrera EKG under 24 timmar med en liten bärbar EKG-bandspelare. Under EKG registreringen ska du leva som vanligt förutom att du inte kan duscha och bada. Efter 24-timmar kan du ta bort elektroderna och lämna tillbaka apparaten (besök 2).

Del 2
Besök 2 (ca 6 timmar):
Mikrodialys
Huden mitt på låret bedövas med bedövningsmedel. Därefter förs två mycket tunna mikrodialyskatetrar (trådtunna slangar) in i lårmuskeln. En vätska cirkulerar långsamt med hjälp av en lite pump och prover tas med regelbundna intervall från vätskan för analys av ämnesomsättningsämnen samt smärt- och inflammationsframkallande substanser. Du kommer även att få utföra ett statiskt muskelarbete med lårmuskeln. Sammantaget tar själva mikrodialysundersökningen 4 timmar och 20 minuter i anspråk och katetrarna avlägsnas. Efter avslutad mikrodialys sker ny bedövning av huden, en 4-6 mm vävnadsprov (biopsi) tas från huden och mikrodialyskatetrarna dras ut.

Styrketräning
Efter genomgången mikrodialysundersökning påbörjas styrketräningen. Denna pågår 2 gånger i veckan under 15 veckor i grupper av 5. Träningen leds av speciellt utbildad sjukgymnast. De första träningspassen tar ca 2 timmar därefter tar träningspassen ca 1 timme i anspråk. Efter avslutad styrketräning efter 15 veckor upprepas alla mätningar (besök 1 och 2). Ett år senare kommer vi att skicka hem samma frågeformulär som besvaras och skickas tillbaka till oss i frankerade kuvert som vi tillhandahåller.

Blodprovet lagras tills vidare i en så kallad biobank vid varje ort. Salivprovet förvaras på Karolinska Institutets Biobank, Stockholm. Proverna förvaras kodade, vilket innebär att de inte kan härledas till dig som person, och de kommer att används endast på det sätt som du givit samtycke till. De kan endast bli aktuella för ett nytt forskningsprojekt efter att du blivit tillfrågad och lämnat ett nytt samtycke och/eller godkännande skett av etikprövningsnämnden (Biobankslagen, SFS 2002:297).

Vilka är riskerna?
Vid undersökning av smärtkänslighet utsätts du för kortvarig smärta som du när som helst kan avbryta. Smärtstimuleringarna avbryts alltid vid ett visst tryck (1500 kPa) och håller sig inom temperaturintervallet (0 – 50°C) för att undvika vävnadsskada. Blodprovstagning följer sedvanliga kliniska rutiner och är inte förenat med allvarliga risker. Registrering av 24-timmars EKG innebär inga risker men innebär att du inte kan duscha och bada under registreringen och kan upplevas som lite obekvämt nattetid. Mikrodialys och hudbiopsi utförs under bedövning med lokalt bedövningsmedel och är inte förenat med smärta. När slangarna är på plats i muskulaturen känns de inte och det känns inte heller när de avlägsnas. En liten risk för infektion eller blödning finns och om tecken på detta skulle uppstå behandlas den på sedvanligt sätt (du kommer att få uppgift om vem du ska kontakta i så fall). Noggrann hygien iakttas för att undvika infektioner. För att kunna bestämma nivåerna av de biokemiska ämnena används en mycket liten dos radioaktiva ämnen i den vätska som genomsköljer de slangar som lagts in i muskulaturen. Strålningen av de radioaktiva ämnena motsvarar 25 % av strålningen vid t ex en röntgenundersökning av en arm. Denna dos är inte förknippad med någon märkbart ökad risk för cancer. Träningsrelaterad smärta kan förekomma vid

styrketräning, men även den är övergående. Undersökningsmetoder och träningsprogrammet är beprövade och leder inte till några bestående bieffekter eller vävnadsskada.

Finns det några fördelar?
Du kommer att få en uppfattning om din fysiska funktion och ditt hälsotillstånd. Om du deltar i styrketräningen kommer du ha goda möjligheter att öka din styrka och att grundlägga goda träningsvanor.

Hantering av data och sekretess
Alla försökspersoner erhåller ett studiespecifikt nummer. All information om dig registreras i en speciell mapp märkt med ditt nummer och all databearbetning sker med avidentifierade data. Alla frågeformulär och ditt försöksprotokoll märks endast med dina initialer och ditt studienummer och alltså inte med ditt fullständiga namn. Dina personuppgifter och ditt kodnummer förvaras separat inlåst i journalskåp. Dina personuppgifter skyddas av den sekretess som anges i journallagen. Originaldata sparas under minst 10 år för att möjliggöra granskning av projektets kvalité. Studiens resultat kommer att presenteras i en vetenskaplig tidskrift på gruppnivå, vilket innebär att det inte går att urskilja enskilda individer.

Hur får jag information om studiens resultat?
Efter att data har analyserats kommer vi att presentera studiens resultat i vetenskapliga artiklar och på olika seminarier och föreläsningar.

Försäkring och ersättning
Försökspersoner som deltar i del 1 av studien erhåller 500 kr efter avslutad undersökning. Försökspersoner som deltar i både del 1 och del 2 får tillgång till träningslokal (alt. gymkort i 6 månader) och 3 000 kr betalas ut efter sista undersökningstillfället dvs. efter mircodialysundersökning/hudbiopsi för andra gången efter avslutad styrketräning. Ersättningen är skattepliktig. Sjukvårdens patientskadeförsäkring gäller för undersökningarna.

Frivillighet
Det är givetvis frivilligt att delta i forskningsprojektet, och Du kan när som helst avbryta Ditt deltagande om Du så önskar utan att ange någon orsak. Frågeformulär, blod- och salivprov märks så att det inte är möjligt att spåra dem till den enskilde, och du har rätt att begära att de förstörs. Vill du inte delta, eller vill du avbryta studien kommer detta inte att påverka ev. sjukvårdsbehandling.

Ansvarig för studien i Göteborg-Alingsås

Docent Kaisa Mannerkorpi, SU/Sjukgymnastikenheten och Avd för Reumatologi och Inflammationsforskning, Sahlgrenska Akademin, Göteborgs Universitet.

För mer information om studien kontakta:

Docent Kaisa Mannerkorpi, SU/Sjukgymnastikenheten, tel 031-3421195, och Avd för Reumatologi och Inflammationsforskning, Sahlgrenska Akademin, Göteborgs Universitet, tel. 031-3424692. Email: kaisa.mannerkorpi@rheuma.gu.se

Kontaktperson i Alingsås: Anette Larsson, leg sjukgymnast, NärRehab Mitten Älvsborg, Herrljunga Vårdcentral, tel 0322-624076

Informerat samtycke till att delta i studien
" Effekten av fysisk träning på sjukdomsmekanismer och symtom vid fibromyalgi."

Jag har både skriftligt och muntligt blivit informerad om studien och den databehandling av personuppgifter som studien innebär. Jag har även fått tillfälle att ställa frågor och fått en kopia av patientinformationen.

Jag är medveten om att mitt deltagande i studien är fullt frivilligt och att jag när som helst och utan närmare förklaring kan avbryta mitt deltagande utan att detta påverkar min nuvarande eller kommande behandling.

Alternativ 1: Jag samtycker till att delta i detta forskningsprojekt i sin helhet (omfattande del 1 och del 2 dvs. delta i styrketräning och genomgå microdialys/hudbiopsi undersökningen före och efter träning).

Ort och datum ______________________

____
Patientens namnteckning

____
Namnförtydligande

Alternativ 2: Jag samtycker till att endast delta i del 1 av detta forskningsprojekt (dvs. inte träna och inte genomgå microdialys/hudbiopsi).

Ort och datum ______________________

____
Patientens namnteckning

____
Namnförtydligande

Jag bekräftar att patienten har fått information om forskningsprojektet och intygar att patienten själv har undertecknat och daterat samtycket.

Ort och datum ______________________

____

Ansvarig sjukgymnast

____

Namnförtydligande

En kopia av denna signerade patientinformation ges till patienten.

Förfågan till friska kvinnor om deltagande som kontrollpersoner i en studie om effekten av fysisk träning på sjukdomsmekanismer och symtom vid smärtsyndromet fibromyalgi .

Bakgrund och syfte.
Vi söker friska kvinnor som kontrollpersoner i en studie av olika typer av träning för kvinnor med långvarig smärta orsakad av fibromyalgi. Det saknas förståelse för sjukdomsmekanismer vid detta smärttillstånd och därmed saknas även effektiv behandling. Många former av träning förbättrar funktion och symtom hos personer med fibromyalgi, men mer kunskaper behövs om effekterna av olika träningsformer likväl som om smärtmekanismer hos personer med fibromyalgi och friska.
Denna studie syftar till att öka förståelsen av smärtmekanismerna hos patienter med fibromyalgi och undersöka effekterna av styrke- respektive avspänningsträning på sjukdomsmekanismer och hälsotillstånd. Eftersom man i tidigare forskning sett att vissa ärftliga (genetiska) faktorer har betydelse vid fibromyalgi kommer ärftliga faktorers inverkan på smärtmekanismer och effekten av träning att undersökas i denna studie. Ökade kunskaper är nödvändiga för att på sikt kunna utveckla mer effektiv behandling.
Vi söker nu således *friska kvinnor utan smärta* som kan utgöra ett jämförelsematerial till våra patienter.

Vem kan delta?
Vi söker friska kvinnor i åldrarna 20-65 år utan smärta som är bosatta i eller närheten av Linköping och som är intresserade av att delta som kontrollpersoner i en undersökning av smärtmekanismer och effekter av träning, vilket beskrivs mer i detalj nedan. Undersökningen består av 2 delar och man kan delta antigen bara i del 1 eller i både del 1 och del 2.

Hur går studien till?

Del 1
Besök 1 (ca 2,5 timmar): Besöket inleds med genomgång av din hälsa. Du får besvara olika frågeformulär avseende din smärta, sömn, ev. psykiska symtom, din fysiska aktivitet och din livskvalitet. Smärtkänslighet för tryck undersöks med s.k. tryckalgometer. Därvid trycker försöksledaren med en stav mot olika delar av din kropp och du får signalera när trycket gör minsta lilla ont, resp. motsvarar en smärta som du skattar som måttligt stark eller mycket stark. Känsligheten för tryck undersöks i vila och under statiskt muskelarbete (du trycker underbenet mot ett fast motstånd).
Känsligheten för värme och kyla undersöks genom att en metallplatta läggs mot låret och du signalerar när du känner minsta lilla värme/kyla, när värmen/kylan gör minsta lilla ont och när du skulle skatta värmesmärtan som måttligt stark eller mycket stark, samt hur ont värmen gör när den ges under 2 minuter. Du kan när som helst avbryta stimuleringen.
Du får genomgå mätningar av din styrka och balans samt utföra ett 6 minuters gångtest. Blodprov (20 ml) tas vid detta besök (utom för de försökspersoner som deltar i mikrodialysundersökningen (del 2), där blodprov tas i samband med denna). Blodprovet kommer att användas för analys av ämnen som är av betydelse för reglering av smärta och inflammation. Salivprov tas för analys av vissa gener som har betydelse för smärtreglering.

De som även kommer att delta i del 2 får registrera EKG under 24 timmar med en liten bärbar EKG-bandspelare. Under EKG registreringen ska du leva som vanligt förutom att du inte kan

duscha och bada. Efter 24-timmar kan du ta bort elektroderna och lämna tillbaka apparaten (besök 2).

Del 2
Besök 2 (ca 6 timmar):
Mikrodialys
Huden mitt på låret bedövas med bedövningsmedel. Därefter förs två mycket tunna mikrodialyskatetrar (trådtunna slangar) in i lårmuskeln. En vätska cirkulerar långsamt med hjälp av en lite pump och prover tas med regelbundna intervall från vätskan för analys av ämnesomsättningsämnen samt smärt- och inflammationsframkallande substanser. Du kommer även att få utföra ett statiskt muskelarbete med lårmuskeln. Sammantaget tar själva mikrodialysundersökningen 4 timmar och 20 minuter i anspråk och katetrarna avlägsnas. Efter avslutad mikrodialys sker ny bedövning av huden, en 4-6 mm vävnadsprov (biopsi) tas från huden och mikrodialyskatetrarna dras ut.

Styrketräning
Efter genomgången mikrodialysundersökning påbörjas styrketräningen. Denna pågår 2 gånger i veckan under 15 veckor i grupper av 5. Träningen leds av speciellt utbildad sjukgymnast. De första träningspassen tar ca 2 timmar därefter tar träningspassen ca 1 timme i anspråk. Efter avslutad styrketräning efter 15 veckor upprepas alla mätningar (besök 1 och 2). Ett år senare kommer vi att skicka hem samma frågeformulär som besvaras och skickas tillbaka till oss i frankerade kuvert som vi tillhandahåller.

Blodprovet lagras tills vidare i en så kallad biobank vid varje ort. Salivprovet lagras på Karolinska Institutets Biobank i Stockholm. Proverna förvaras kodade, vilket innebär att de inte kan härledas till dig som person, och de kommer att används endast på det sätt som du givit samtycke till. De kan endast bli aktuella för ett nytt forskningsprojekt efter att du blivit tillfrågad och lämnat ett nytt samtycke och/eller godkännande skett av etikprövningsnämnden (Biobankslagen, SFS 2002:297).

Vilka är riskerna?
Vid undersökning av smärtkänslighet utsätts du för kortvarig smärta som du när som helst kan avbryta. Smärtstimuleringarna avbryts alltid vid ett visst tryck (1500 kPa) och håller sig inom temperaturintervallet (0 – 50°C) för att undvika vävnadsskada. Blodprovstagning följer sedvanliga kliniska rutiner och är inte förenat med allvarliga risker. Registrering av 24-timmars EKG innebär inga risker men innebär att du inte kan duscha och bada under registreringen och kan upplevas som lite obekvämt nattetid. Mikrodialys och hudbiopsi utförs under bedövning med lokalt bedövningsmedel och är inte förenat med smärta. När slangarna är på plats i muskulaturen känns de inte och det känns inte heller när de avlägsnas. En liten risk för infektion eller blödning finns och om tecken på detta skulle uppstå behandlas den på sedvanligt sätt (du kommer att få uppgift om vem du ska kontakta i så fall). Noggrann hygien iakttas för att undvika infektioner. För att kunna bestämma nivåerna av de biokemiska ämnena används en mycket liten dos radioaktiva ämnen i den vätska som genomsköljer de slangar som lagts in i muskulaturen. Strålningen av de radioaktiva ämnena motsvarar 25 % av strålningen vid t ex en röntgenundersökning av en arm. Denna dos är inte förknippad med någon märkbart ökad risk för cancer. Träningsrelaterad smärta kan förekomma vid styrketräning, men även den är övergående. Undersökningsmetoder och träningsprogrammet är beprövade och leder inte till några bestående bieffekter eller vävnadsskada.

Finns det några fördelar?
Du kommer att få en uppfattning om din fysiska funktion och ditt hälsotillstånd. Om du deltar i styrketräningen kommer du ha goda möjligheter att öka din styrka och att grundlägga goda träningsvanor.

Hantering av data och sekretess
Alla försökspersoner erhåller ett studiespecifikt nummer. All information om dig registreras i en speciell mapp märkt med ditt nummer och all databearbetning sker med avidentifierade data. Alla frågeformulär och ditt försöksprotokoll märks endast med dina initialer och ditt studienummer och alltså inte med ditt fullständiga namn. Dina personuppgifter och ditt kodnummer förvaras separat inlåst i journalskåp. Dina personuppgifter skyddas av den sekretess som anges i journallagen. Originaldata sparas under minst 10 år för att möjliggöra granskning av projektets kvalité. Studiens resultat kommer att presenteras i en vetenskaplig tidskrift på gruppnivå, vilket innebär att det inte går att urskilja enskilda individer.

Hur får jag information om studiens resultat?
Efter att data har analyserats kommer vi att presentera studiens resultat i vetenskapliga artiklar och på olika seminarier och föreläsningar.

Försäkring och ersättning
Försökspersoner som deltar i del 1 av studien erhåller 500 kr efter avslutad undersökning. Försökspersoner som deltar i både del 1 och del 2 får tillgång till träningslokal (alt. gymkort i 6 månader) och 3 000 kr betalas ut efter sista undersökningstillfället dvs. efter mircodialysundersökning/hudbiopsi för andra gången efter avslutad styrketräning. Ersättning är skattepliktig. Sjukvårdens patientskadeförsäkring gäller för undersökningarna.

Frivillighet
Det är givetvis frivilligt att delta i forskningsprojektet, och Du kan när som helst avbryta Ditt deltagande om Du så önskar utan att ange någon orsak. Frågeformulär, blod- och salivprov märks så att det inte är möjligt att spåra dem till den enskilde, och du har rätt att begära att de förstörs. Vill du inte delta, eller vill du avbryta studien kommer detta inte att påverka ev. sjukvårdsbehandling.

Ansvarig för studien i Linköping

Prof., Överläkare Björn Gerdle, Smärt och Rehabcentrum, Universitetssjukhuset, 581 85 Linköping.

För mer information om studien kontakta:

Docent, Överläkare Britt Larsson, Smärt och Rehabcentrum, Universitetssjukhuset, Linköping, e-mail: Britt.Larsson@inr.liu.se eller

Sekreterare Natalie Baldew, Smärt och Rehabcentrum, Universitetssjukhuset, Linköping, tfn: 010-1034453.

Informerat samtycke till att delta i studien
" Effekten av fysisk träning på sjukdomsmekanismer och symtom vid fibromyalgi."

Jag har både skriftligt och muntligt blivit informerad om studien och den databehandling av personuppgifter som studien innebär. Jag har även fått tillfälle att ställa frågor och fått en kopia av patientinformationen.

Jag är medveten om att mitt deltagande i studien är fullt frivilligt och att jag när som helst och utan närmare förklaring kan avbryta mitt deltagande utan att detta påverkar min nuvarande eller kommande behandling.

Alternativ 1: Jag samtycker till att delta i detta forskningsprojekt i sin helhet (omfattande del 1 och del 2 dvs. delta i styrketräning och genomgå microdialys/hudbiopsi undersökningen före och efter träning).

Ort och datum ______________________

____
Patientens namnteckning

____
Namnförtydligande

Alternativ 2: Jag samtycker till att endast delta i del 1 av detta forskningsprojekt (dvs. inte träna och inte genomgå microdialys/hudbiopsi).

Ort och datum ______________________

____
Patientens namnteckning

____
Namnförtydligande

Jag bekräftar att patienten har fått information om forskningsprojektet och intygar att patienten själv har undertecknat och daterat samtycket.

Ort och datum ______________________

____
Ansvarig sjukgymnast

____
Namnförtydligande

En kopia av denna signerade patientinformation ges till patienten.

Förfågan till friska kvinnor om deltagande som kontrollpersoner i en studie om effekten av fysisk träning på sjukdomsmekanismer och symtom vid smärtsyndromet fibromyalgi .

Bakgrund och syfte.
Vi söker friska kvinnor som kontrollpersoner i en studie av olika typer av träning för kvinnor med långvarig smärta orsakad av fibromyalgi. Det saknas förståelse för sjukdomsmekanismer vid detta smärttillstånd och därmed saknas även effektiv behandling. Många former av träning förbättrar funktion och symtom hos personer med fibromyalgi, men mer kunskaper behövs om effekterna av olika träningsformer likväl som om smärtmekanismer hos personer med fibromyalgi och friska.
Denna studie syftar till att öka förståelsen av smärtmekanismerna hos patienter med fibromyalgi och undersöka effekterna av styrke- respektive avspänningsträning på sjukdomsmekanismer och hälsotillstånd. Eftersom man i tidigare forskning sett att vissa ärftliga (genetiska) faktorer har betydelse vid fibromyalgi kommer ärftliga faktorers inverkan på smärtmekanismer och effekten av träning att undersökas i denna studie. Ökade kunskaper är nödvändiga för att på sikt kunna utveckla mer effektiv behandling.
Vi söker nu således *friska kvinnor utan smärta* som kan utgöra ett jämförelsematerial till våra patienter.

Vem kan delta?
Vi söker friska kvinnor i åldrarna 20-65 år utan smärta som är bosatta i eller närheten av Stockholm och som är intresserade av att delta som kontrollpersoner i en undersökning av smärtmekanismer och effekter av träning, vilket beskrivs mer i detalj nedan. Undersökningen består av 2 delar och man kan delta antigen bara i del 1 eller i både del 1 och del 2.

Hur går studien till?

Del 1
Besök 1 (ca 2,5 timmar): Besöket inleds med genomgång av din hälsa. Du får besvara olika frågeformulär avseende din smärta, sömn, ev. psykiska symtom, din fysiska aktivitet och din livskvalitet. Smärtkänslighet för tryck undersöks med s.k. tryckalgometer. Därvid trycker försöksledaren med en stav mot olika delar av din kropp och du får signalera när trycket gör minsta lilla ont, resp. motsvarar en smärta som du skattar som måttligt stark eller mycket stark. Känsligheten för tryck undersöks i vila och under statiskt muskelarbete (du trycker underbenet mot ett fast motstånd).
Känsligheten för värme och kyla undersöks genom att en metallplatta läggs mot låret och du signalerar när du känner minsta lilla värme/kyla, när värmen/kylan gör minsta lilla ont och när du skulle skatta värmesmärtan som måttligt stark eller mycket stark, samt hur ont värmen gör när den ges under 2 minuter. Du kan när som helst avbryta stimuleringen.
Du får genomgå mätningar av din styrka och balans samt utföra ett 6 minuters gångtest. Blodprov (20 ml) tas vid detta besök (utom för de försökspersoner som deltar i mikrodialysundersökningen (del 2), där blodprov tas i samband med denna). Blodprovet kommer att användas för analys av ämnen som är av betydelse för reglering av smärta och inflammation. Salivprov tas för analys av vissa gener som har betydelse för smärtreglering.

De som även kommer att delta i del 2 får registrera EKG under 24 timmar med en liten bärbar EKG-bandspelare. Under EKG registreringen ska du leva som vanligt förutom att du inte kan

duscha och bada. Efter 24-timmar kan du ta bort elektroderna och lämna tillbaka apparaten (besök 2).

Del 2
Besök 2 (ca 6 timmar):
Mikrodialys
Huden mitt på låret bedövas med bedövningsmedel. Därefter förs två mycket tunna mikrodialyskatetrar (trådtunna slangar) in i lårmuskeln. En vätska cirkulerar långsamt med hjälp av en lite pump och prover tas med regelbundna intervall från vätskan för analys av ämnesomsättningsämnen samt smärt- och inflammationsframkallande substanser. Du kommer även att få utföra ett statiskt muskelarbete med lårmuskeln. Sammantaget tar själva mikrodialysundersökningen 4 timmar och 20 minuter i anspråk och katetrarna avlägsnas. Efter avslutad mikrodialys sker ny bedövning av huden, en 4-6 mm vävnadsprov (biopsi) tas från huden och mikrodialyskatetrarna dras ut.

Hjärnavbildningsundersökning
Innan träningen påbörjas kommer alla som lottats till styrketräning att kallas till en hjärnavbildningsundersökning med funktionell magnetisk resonans (fMRI). Magnetresonans (MR) har revolutionerat möjligheter att återge en detaljerad bild av hjärnan och dess funktion, och används idag för att diagnostisera många olika sjukdomar. Tekniken har använts för att studera hjärnans funktion i många smärttillstånd, så som fibromyalgi.

Besök 3 (ca 1 timme)
Vid det fjärde besöket undersöker vi din smärtkänslighet för tryck mot tumnagel för att fastställa vilket tryck som ska användas vid fMRI undersökningen.

Besök 4 (ca 1 timme)
Du kommer att få ligga i en magnetröntgenkamera och samtidigt utföres tryck mot din tumnageln.

Styrketräning
Efter genomgången mikrodialys- och fMRIundersökning påbörjas styrketräningen. Denna pågår 2 gånger i veckan under 15 veckor i grupper av 5. Träningen leds av speciellt utbildad sjukgymnast. De första träningspassen tar ca 2 timmar därefter tar träningspassen ca 1 timme i anspråk. Efter avslutad styrketräning efter 15 veckor upprepas alla mätningar (besök 1 - 4). Ett år senare kommer vi att skicka hem samma frågeformulär som besvaras och skickas tillbaka till oss i frankerade kuvert som vi tillhandahåller.

Blodprovet lagras tills vidare i en så kallad biobank vid varje ort. Salivprovet lagras på Karolinska Institutets Biobank i Stockholm. Proverna förvaras kodade, vilket innebär att de inte kan härledas till dig som person, och de kommer att används endast på det sätt som du givit samtycke till. De kan endast bli aktuella för ett nytt forskningsprojekt efter att du blivit tillfrågad och lämnat ett nytt samtycke och/eller godkännande skett av etikprövningsnämnden (Biobankslagen, SFS 2002:297).

Vilka är riskerna?
Vid undersökning av smärtkänslighet utsätts du för kortvarig smärta som du när som helst kan avbryta. Smärtstimuleringarna avbryts alltid vid ett visst tryck (1500 kPa) och håller sig inom temperaturintervallet (0 – 50°C) för att undvika vävnadsskada. Blodprovstagning följer

sedvanliga kliniska rutiner och är inte förenat med allvarliga risker. Registrering av 24-timmars EKG innebär inga risker men innebär att du inte kan duscha och bada under registreringen och kan upplevas som lite obekvämt nattetid. Mikrodialys och hudbiopsi utförs under bedövning med lokalt bedövningsmedel och är inte förenat med smärta. När slangarna är på plats i muskulaturen känns de inte och det känns inte heller när de avlägsnas. En liten risk för infektion eller blödning finns och om tecken på detta skulle uppstå behandlas den på sedvanligt sätt (du kommer att få uppgift om vem du ska kontakta i så fall). Noggrann hygien iakttas för att undvika infektioner. För att kunna bestämma nivåerna av de biokemiska ämnena används en mycket liten dos radioaktiva ämnen i den vätska som genomsköljer de slangar som lagts in i muskulaturen. Strålningen av de radioaktiva ämnena motsvarar 25 % av strålningen vid t ex en röntgenundersökning av en arm. Denna dos är inte förknippad med någon märkbart ökad risk för cancer. fMRI undersökningen är ofarlig men kan upplevas som skrämmande av människor med tendens till klaustrofobi. Träningsrelaterad smärta kan förekomma vid styrketräning, men även den är övergående. Undersökningsmetoder och träningsprogrammet är beprövade och leder inte till några bestående bieffekter eller vävnadsskada.

Finns det några fördelar?
Du kommer att få en uppfattning om din fysiska funktion och ditt hälsotillstånd. Om du deltar i styrketräningen kommer du ha goda möjligheter att öka din styrka och att grundlägga goda träningsvanor.

Hantering av data och sekretess
Alla försökspersoner erhåller ett studiespecifikt nummer. All information om dig registreras i en speciell mapp märkt med ditt nummer och all databearbetning sker med avidentifierade data. Alla frågeformulär och ditt försöksprotokoll märks endast med dina initialer och ditt studienummer och alltså inte med ditt fullständiga namn. Dina personuppgifter och ditt kodnummer förvaras separat inlåst i journalskåp. Dina personuppgifter skyddas av den sekretess som anges i journallagen. Originaldata sparas under minst 10 år för att möjliggöra granskning av projektets kvalité. Studiens resultat kommer att presenteras i en vetenskaplig tidskrift på gruppnivå, vilket innebär att det inte går att urskilja enskilda individer.

Hur får jag information om studiens resultat?
Efter att data har analyserats kommer vi att presentera studiens resultat i vetenskapliga artiklar och på olika seminarier och föreläsningar.

Försäkring och ersättning
Försökspersoner som deltar i del 1 av studien erhåller 500 kr efter avslutad undersökning. Försökspersoner som deltar i både del 1 och del 2 får tillgång till träningslokal (alt. gymkort i 6 månader) och 3 500 kr betalas ut efter sista undersökningstillfället dvs. efter mircodialysundersökning/hudbiopsi för andra gången efter avslutad styrketräning. Ersättningen är skattepliktig. Sjukvårdens patientskadeförsäkring gäller för undersökningarna.

Frivillighet
Det är givetvis frivilligt att delta i forskningsprojektet, och Du kan när som helst avbryta Ditt deltagande om Du så önskar utan att ange någon orsak. Frågeformulär, blod- och salivprov märks så att det inte är möjligt att spåra dem till den enskilde, och du har rätt att begära att de förstörs. Vill du inte delta, eller vill du avbryta studien kommer detta inte att påverka ev. sjukvårdsbehandling.

Ansvarig för studien i Stockholm
Docent Eva Kosek, Institutionen för Klinisk Neurovetenskap, Karolinska Institutet, Retziusväg 8, 171 77 Stockholm.

För mer information om studien kontakta: Med.Dr. Monika Lövgren, Rehabiliteringsmedicinska Universitetskliniken, Danderyds sjukhus AB, 182 88, Stockholm. E-mail: Monika.Lofgren@ds.se, Tfn: 08-655 78 13.
Med. Dr. Leg.Läk. Indre Ljungar, Rehabiliteringsmedicinska Universitetskliniken, Danderyds sjukhus AB, 182 88, Stockholm. E-mail: Indre.Ljungar@ds.se.

Informerat samtycke till att delta i studien
" Effekten av fysisk träning på sjukdomsmekanismer och symtom vid fibromyalgi."

Jag har både skriftligt och muntligt blivit informerad om studien och den databehandling av personuppgifter som studien innebär. Jag har även fått tillfälle att ställa frågor och fått en kopia av patientinformationen.

Jag är medveten om att mitt deltagande i studien är fullt frivilligt och att jag när som helst och utan närmare förklaring kan avbryta mitt deltagande utan att detta påverkar min nuvarande eller kommande behandling.

Alternativ 1: Jag samtycker till att delta i detta forskningsprojekt i sin helhet (omfattande del 1 och del 2 dvs. delta i styrketräning och genomgå microdialys/hudbiopsi undersökningen före och efter träning).

Ort och datum ______________________

____
Patientens namnteckning

____
Namnförtydligande

Alternativ 2: Jag samtycker till att endast delta i del 1 av detta forskningsprojekt (dvs. inte träna och inte genomgå microdialys/hudbiopsi).

Ort och datum ______________________

____
Patientens namnteckning

____
Namnförtydligande

Jag bekräftar att patienten har fått information om forskningsprojektet och intygar att patienten själv har undertecknat och daterat samtycket.

Ort och datum ______________________

____
Ansvarig sjukgymnast

____
Namnförtydligande

En kopia av denna signerade patientinformation ges till patienten.

[illegible]

Stockholms läns landsting

Affektiva mottagningen M59
Fax 585 866 30
Tel 585 866 26, 585 866 34

PATIENTUPPGIFTER:

AUDIT

NAMN: ....

PERS-NR: ....

SIGNATUR: .... DATUM: ....

AUDIT

Här är ett antal frågor om dina alkoholvanor. Vi är tacksamma om du svarar så noggrant och ärligt som möjligt genom att markera det alternativ som gäller för dig. ☐ **Man** ☐ **Kvinna** **Ålder** ☐

Med ett "glas" menas: 50 cl folköl · 33 cl starköl · 1 glas rött el. vitt vin · 1 litet glas starkvin · 4 cl sprit t ex whisky

FRÅGOR	Poäng 0	1	2	3	4	Dina poäng
Hur ofta dricker du alkohol?	Aldrig	1 gång/mån eller mer sällan	2-4 ggr /mån	2-3 ggr /vecka	4 ggr/vecka eller mer	
Hur många "glas" (se exempel) dricker du en typisk dag då du dricker alkohol?	1-2	3-4	5-6	7-9	10 eller fler	
Hur ofta dricker du sex sådana "glas" eller mer vid samma tillfälle?	Aldrig	Mer sällan än 1 gång/mån	Varje månad	Varje vecka	Dagligen eller nästan varje dag	
Hur ofta under det senaste året har du inte kunnat sluta dricka sedan du börjat?	Aldrig	Mer sällan än 1 gång/mån	Varje månad	Varje vecka	Dagligen eller nästan varje dag	
Hur ofta under det senaste året har du låt bli att göra något som du borde för att du drack?	Aldrig	Mer sällan än 1 gång/mån	Varje månad	Varje vecka	Dagligen eller nästan varje dag	
Hur ofta under senaste året har du behövt en "drink" på morgonen efter mycket drickande dagens innan?	Aldrig	Mer sällan än 1 gång/mån	Varje månad	Varje vecka	Dagligen eller nästan varje dag	
Hur ofta under det senaste året har du haft skuldkänslor eller samvetsförebråelser på grund av ditt drickande?	Aldrig	Mer sällan än 1 gång/mån	Varje månad	Varje vecka	Dagligen eller nästan varje dag	
Hur ofta under senaste året har du druckit så att du dagen efter inte kommit ihåg vad du sagt eller gjort?	Aldrig	Mer sällan än 1 gång/mån	Varje månad	Varje vecka	Dagligen eller nästan varje dag	
Har du eller någon annan blivit skadad på grund av ditt drickande?	Nej		Ja, men ej under sista året		Ja, under det senaste året	
Har en släkting eller vän, en läkare (eller någon annan inom sjukvården) oroat sig över ditt drickande eller antytt att du borde minska ned på det?	Nej		Ja, men ej under sista året		Ja, under det senaste året	

Har du besvarat alla frågor? Tack för din medverkan!

Översatt och berarbetat av prof Hans Bergman, Karolinska Institutet, Stockholm.

VAS

Var god markera med ett streck på linjen:

Hur stark är din smärta just nu?

Ingen smärta Värsta tänkbara smärta _____mm

Hur stark har din genomsnittliga smärta varit under senaste veckan?

Ingen smärta Värsta tänkbara smärta _____mm

Hur stark var din smärta när den var som lindrigast (senaste veckan)?

Ingen smärta Värsta tänkbara smärta _____mm

Hur stark var din smärta när den var som värst/starkast (senaste veckan)?

Ingen smärta Värsta tänkbara smärta _____mm

Var god skugga alla de områden på kroppen där du just nu känner att du har smärta (ont/värk).

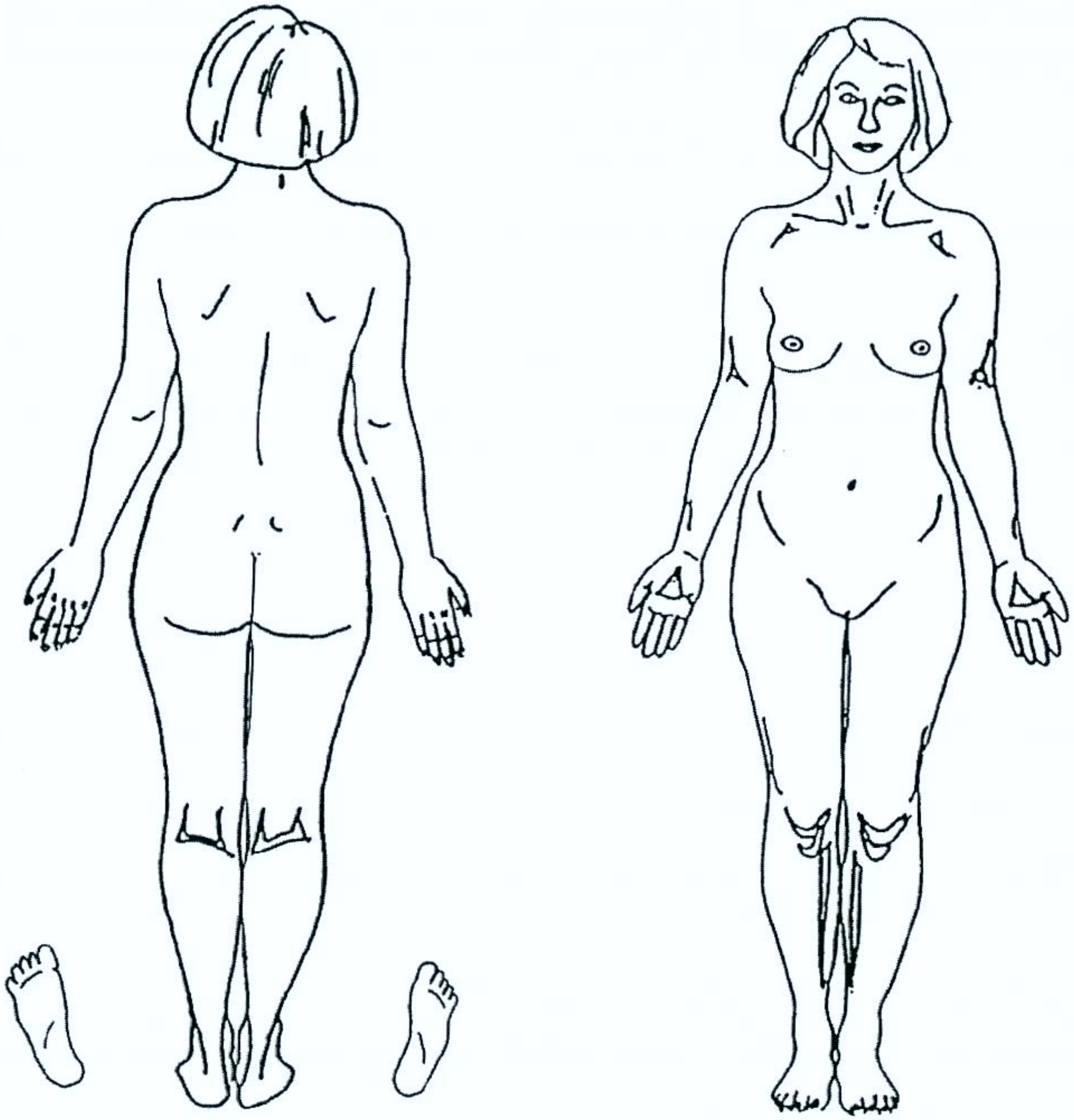

PDI

Skattningsskalorna i detta avsnitt är gjorda för att mäta till vilken grad olika områden av ditt liv störs av långvarig smärta. **Med andra ord, vi vill veta hur mycket din smärta hindrar dig från att göra de saker du normalt skulle göra, eller från att göra dem så bra som du normalt skulle göra.** Här nedanför finns sju typer av aktiviteter beskrivna. **Ringa in den siffra som bäst beskriver den grad av hinder du vanligen upplever**, inte bara när smärtan är som värst. 0 betyder inga hinder alls, och 10 innebär att alla de aktiviteter som du vanligen skulle engagera dig i har blivit helt hindrade av din smärta.

1. *Ansvar för hemmet och familjen.* Aktiviter som har att göra med hemmet och familjen. De innefattar sysslor och uppgifter som du gör hemma, t ex trädgårdsarbete, och ärenden eller tjänster för andra familjemedlemmar, t ex att köra barnen till skolan.	1 2 3 4 5 6 7 8 9 10<br>Inga hinder … helt hindrande
2. *Rekreation.* Hobbies, sport, idrott och andra liknade fritidsaktiviteter.	1 2 3 4 5 6 7 8 9 10<br>Inga hinder … helt hindrande
3. *Social aktivitet.* Aktiviteter med vänner och bekanta utanför familjen, t ex fester, teater, konserter, att gå ut och äta, och liknande.	1 2 3 4 5 6 7 8 9 10<br>Inga hinder … helt hindrande
4. *Sysselsättning.* Aktiviteter som är en del av eller som direkt har att göra med ens arbete. Även oavlönat arbete, t ex föreningsarbete, räknas in här.	1 2 3 4 5 6 7 8 9 10<br>Inga hinder … helt hindrande
5. *Sexualliv.* Frekvens och kvalitet i sexuallivet.	1 2 3 4 5 6 7 8 9 10<br>Inga hinder … helt hindrande
6. *Personlig aktivitet.* Aktiviteter som innefattar det man behöver göra för att vara oberoende i det dagliga livet, t ex att duscha, köra bil, klä på sig, etc.	1 2 3 4 5 6 7 8 9 10<br>Inga hinder … helt hindrande
7. *Livsuppehållande aktivitet.* Grundläggande livsuppehållande handlingar, såsom att äta, sova och andas.	1 2 3 4 5 6 7 8 9 10<br>Inga hinder … helt hindrande

Dessa frågor har ställt samman för att hjälpa oss att förstå hur du mår. Läs varje påstående och sätt ett tydligt kryss i rutan till vänster om det svar som kommer närmast hur du har känt dig under veckan som gått. Sitt inte och fundera för länge. Det första svar som dyker upp för dig på varje påstående är antagligen riktigare än ett svar du tänkt på länge.

HAD

---

1. Jag känner mig spänd eller "uppskruvad"

- ☐ För det mesta
- ☐ Ofta
- ☐ Då och då
- ☐ Inte alls

2. Jag uppskattar samma saker som förut

- ☐ Precis lika mycket
- ☐ Inte lika mycket
- ☐ Bara lite
- ☐ Knappast alls

3. Jag får en slags känsla av rädsla som om någonting förfärligt håller på att hända

- ☐ Alldeles bestämt och rätt illa
- ☐ Ja, men inte så illa
- ☐ Lite, men det oroar mig inte
- ☐ Inte alls

4. Jag kan skratta och se saker från den humoristiska sidan

- ☐ Lika mycket som jag alltid kunnat
- ☐ Inte riktigt lika mycket nu
- ☐ Absolut inte så mycket nu
- ☐ Inte alls

5. Oroande tankar kommer för mig

- ☐ Mycket ofta
- ☐ Ofta
- ☐ Då och då men inte så ofta
- ☐ Bara någon enstaka gång

6. Jag känner mig glad

- ☐ Inte alls
- ☐ Inte ofta
- ☐ Ibland
- ☐ För det mesta

7. Jag kan sitta i lugn och ro och känna mig avspänd

- ☐ Absolut
- ☐ Oftast
- ☐ Inte ofta
- ☐ Inte alls

8. Jag känner mig som om jag gick på "lågt varv"

- ☐ Nästan jämt
- ☐ Mycket ofta
- ☐ Ibland
- ☐ Inte alls

9. Jag får en slags känsla av rädsla som om jag hade "fjärilar i magen"

- ☐ Inte alls
- ☐ Någon gång
- ☐ Rätt ofta
- ☐ Mycket ofta

10. Jag har tappat intresset för mitt utseende

- ☐ Absolut
- ☐ Jag bryr mig inte så mycket om det som jag borde
- ☐ Jag kanske inte bryr mig om det riktigt så mycket
- ☐ Jag bryr mig precis lika mycket om det som förut

11. Jag känner mig rastlös som om jag måste vara på språng

- ☐ Verkligen mycket
- ☐ En hel del
- ☐ Inte så mycket
- ☐ Inte alls

12. Jag ser fram emot saker och ting med glädje

- ☐ Lika mycket som förut
- ☐ Något mindre än jag brukade
- ☐ Klart mindre än jag brukade
- ☐ Nästan inte alls

13. Jag får plötsliga panikkänslor

- ☐ Verkligen ofta
- ☐ Rätt ofta
- ☐ Inte så ofta
- ☐ Inte alls

14. Jag kan njuta av en bra bok, ett bra radio eller TV-program

- ☐ Ofta
- ☐ Ibland
- ☐ Inte så ofta
- ☐ Mycket sällan

HAD: Zigmond & Snaith, 1983. Acta Psychiatr Scand, 67: 361-70; © HRQL Gruppen HB, 2000

PCS

Vi är intresserade av de tankar och känslor du har <u>när du har ont</u>. Använd skalan nedan för att skatta i vilken utsträckning dessa tankar och känslor gäller just för dig.

**När jag har ont…**	**Inte alls**	**I liten utsträckning**	**I måttlig utsträckning**	**I stor utsträckning**	**Hela tiden**
1. Oroar jag mig jämt för att smärtan inte ska gå över.	0	1	2	3	4
2. Känner jag att jag inte orkar fortsätta.	0	1	2	3	4
3. Är det förfärligt och jag tror att det aldrig kommer bli bra	0	1	2	3	4
4. Är det fruktansvärt och jag känner att det överväldigar mig.	0	1	2	3	4
5. Känner jag att jag inte står ut med det längre.	0	1	2	3	4
6. Blir jag rädd att smärtan ska förvärras.	0	1	2	3	4
7. Tänker jag på andra smärtsamma erfarenheter.	0	1	2	3	4
8. Väntar jag otåligt på att smärtan ska försvinna.	0	1	2	3	4
9. Kan jag inte sluta tänka på det.	0	1	2	3	4
10. Tänker jag hela tiden på hur ont det gör.	0	1	2	3	4
11. Tänker jag hela tiden på hur mycket jag vill att smärtan ska gå över.	0	1	2	3	4
12. Finns det ingenting som jag kan göra för att lindra smärtan.	0	1	2	3	4
13. Undrar jag om något allvarligt har hänt mig.	0	1	2	3	4

CPAQ1

CPAQ. Nedan finner du ett antal påståenden. Skatta hur sant varje påstående är för dig genom att ringa in en siffra. Använd skalan nedan för att göra dina val.

Exempel: Om du anser att ett påstående är "alltid sant" ringar du in siffran sex.

**0**	**1**	**2**	**3**	**4**	**5**	**6**
**Aldrig sant**	**Mycket sällan sant**	**Sällan sant**	**Sant ibland**	**Ofta sant**	**Nästan alltid sant**	**Alltid sant**

1. Jag fortsätter att leva som vanligt oavsett hur mycket smärta jag har.	0	1	2	3	4	5	6
2. Mitt liv fungerar bra även fast jag har kronisk smärta.	0	1	2	3	4	5	6
3. Det är OK att uppleva smärta.	0	1	2	3	4	5	6
4. Jag skulle gärna avstå ifrån viktiga saker i mitt liv för att kunna kontrollera min smärta bättre.	0	1	2	3	4	5	6
5. Det är inte nödvändigt för mig att kontrollera min smärta för att kunna hantera mitt liv bra.	0	1	2	3	4	5	6
6. Även om saker har förändrats lever jag ett normalt liv trots min kroniska smärta.	0	1	2	3	4	5	6
7. Jag måste fokusera på att bli av med min smärta.	0	1	2	3	4	5	6
8. Det finns många aktiviteter som jag gör när jag känner smärta.	0	1	2	3	4	5	6
9. Jag lever ett fullvärdigt liv trots att jag har kronisk smärta.	0	1	2	3	4	5	6
10. Att kontrollera smärtan är mindre viktigt än andra mål i mitt liv.	0	1	2	3	4	5	6

CPAQ2

0	1	2	3	4	5	6
Aldrig sant	Mycket sällan sant	Sällan sant	Sant ibland	Ofta sant	Nästan alltid sant	Alltid sant

11. Mina tankar och känslor om smärtan måste förändras innan jag kan gå vidare med mitt liv.	0	1	2	3	4	5	6
12. Trots smärtan lever jag mitt liv som planerat.	0	1	2	3	4	5	6
13. Att hålla min smärta under kontroll har högsta prioritet när jag gör något.	0	1	2	3	4	5	6
14. Innan jag kan göra upp några bestämda planer måste jag få viss kontroll över min smärta.	0	1	2	3	4	5	6
15. När min smärta ökar kan jag fortfarande sköta det jag har ansvar för.	0	1	2	3	4	5	6
16. Om jag kan kontrollera mina negativa tankar kring smärtan får jag bättre kontroll över mitt liv.	0	1	2	3	4	5	6
17. Jag undviker att sätta mig i situationer där smärtan skulle kunna öka.	0	1	2	3	4	5	6
18. Min oro och rädsla över vad smärtan kan göra mig är sann.	0	1	2	3	4	5	6
19. Det är en lättnad att inse att jag inte behöver förändra min smärta för att gå vidare med mitt liv.	0	1	2	3	4	5	6
20. Jag måste kämpa för att göra saker när jag har smärta.	0	1	2	3	4	5	6

FABQ 1/2

Universitetssjukhuset Örebro
Yrkes- och miljömedicinska kliniken
ÖREBRO LÄNS LANDSTING

FABQ

Namn: .... Datum: ....

Nedan följer olika erfarenheter av smärta som andra patienter har delgett oss. Var vänlig och ringa in lämplig siffra från 0-6 för varje påstående för att ange i vilken utsträckning fysiska aktiviteter – som att böja sig, lyfta, promenera eller köra bil – påverkar eller kan tänkas påverka Din ryggsmärta.

	**Instämmer inte alls**						**Instämmer helt**
1 Min smärta orsakades av fysisk aktivitet	0	1	2	3	4	5	6
2 Fysisk aktivitet ökar smärtan	0	1	2	3	4	5	6
3 Fysisk aktivitet kan skada min rygg	0	1	2	3	4	5	6
4 Jag bör inte utföra fysiska aktiviteter som ger ökad smärta	0	1	2	3	4	5	6
5 Jag kan inte utföra fysiska aktiviteter som ger ökad smärta	0	1	2	3	4	5	6

Följande påstående handlar om hur Ditt vanliga arbete påverkar eller kan tänkas påverka Din ryggsmärta.

	**Instämmer inte alls**						**Instämmer helt**
6 Min smärta orsakades av mitt arbete eller av en olycka på arbetet	0	1	2	3	4	5	6
7 Mitt arbete förvärrade smärtan	0	1	2	3	4	5	6
8 Jag har krävt ekonomisk ersättning för min smärta	0	1	2	3	4	5	6
9 Mitt arbete är för tungt för mig	0	1	2	3	4	5	6

FABQ 2/2

Universitetssjukhuset Örebro
Yrkes- och miljömedicinska kliniken

Örebro läns landsting

	Instämmer inte alls						Instämmer helt
10 Mitt arbete ger eller kan tänkas ge ökad smärta	0	1	2	3	4	5	6
11 Mitt arbete kan skada min rygg	0	1	2	3	4	5	6
12 Jag bör inte utföra mitt vanliga arbete med den smärta jag har för närvarande	0	1	2	3	4	5	6
13 Jag kan inte utföra mitt vanliga arbete med den smärta jag har för närvarande	0	1	2	3	4	5	6
14 Jag kan inte utföra mitt vanliga arbete förrän min smärta har behandlats	0	1	2	3	4	5	6
15 Jag tror inte att jag kommer att återgå till mitt vanliga arbete inom tre månader	0	1	2	3	4	5	6
16 Jag tror inte att jag någonsin kommer att kunna återgå till mitt tidigare arbete	0	1	2	3	4	5	6

Namn:

Datum:

Exercise Self-Efficacy Scale

Människor som tränar regelbundet, t ex går på gymnastik, gör övningar hemma, eller motionerar på annat sätt, kan tycka att vissa omständigheter känns som hinder för träning. Man kan t ex ha ont om tid, känna sig hängig eller tycka det är tråkigt. Vi är intresserade av att veta hur Du tror att Du skulle klara av att träna även om det tog emot av olika anledningar.

Här nedan finner Du några situationer beskrivna, som andra människor har ansett vara hinder för träning.
Ringa in den siffra på skalan efter varje påstående som bäst beskriver hur säker Du är på Din förmåga att genomföra ett träningsprogram under olika omständigheter. Om Din förmåga varierar från dag till dag, ringa in den siffra som beskriver hur det vanligen är.

1 betyder att Du inte alls är säker, och 10 betyder att Du är mycket säker på Din förmåga att genomföra ett träningsprogram.

Hur säker är Du på Din förmåga att genomföra ett träningsprogram:

1. När Du har långa arbetsdagar	**1**	**2**	**3**	**4**	**5**	**6**	**7**	**8**	**9**	**10**
2. När Du är fysiskt trött	**1**	**2**	**3**	**4**	**5**	**6**	**7**	**8**	**9**	**10**
3. När det känns tråkigt att träna	**1**	**2**	**3**	**4**	**5**	**6**	**7**	**8**	**9**	**10**
4. När Du har små skador	**1**	**2**	**3**	**4**	**5**	**6**	**7**	**8**	**9**	**10**
5. När andra saker kräver Din tid	**1**	**2**	**3**	**4**	**5**	**6**	**7**	**8**	**9**	**10**
6. När familjeplikter kräver Din tid	**1**	**2**	**3**	**4**	**5**	**6**	**7**	**8**	**9**	**10**

Dzewaltowski (1989)
Yordi & Lent (1993)

EPA 1/2

Namn ______________________ Födelseår ________ Datum __________

Upplevelse av motion

Här nedan finner Du ett antal uttalanden som andra människor har använt när de beskrivit sin upplevelse av att röra på sig eller motionera. En del uttalanden är positiva medan andra är negativa. Ringa in den siffra på skalan som bäst stämmer överens med din upplevelse under de senaste fyra veckorna.

Med motion avser vi här **alla** former av fysisk aktivitet, så som promenader (även till arbete och affär), gymnastik, hemövningar, bassängträning, bollspel, osv.

1. Jag får mer ont *medan* jag motionerar.
   Stämmer helt 1 2 3 4 5 6 7 Stämmer inte alls

2. Jag får mer ont *efter* att jag har motionerat.
   Stämmer helt 1 2 3 4 5 6 7 Stämmer inte alls

3. Jag känner mig rörligare och mjukare medan jag motionerar.
   Stämmer helt 1 2 3 4 5 6 7 Stämmer inte alls

4. Jag känner mig rörligare och mjukare *efter* att jag har motionerat.
   Stämmer helt 1 2 3 4 5 6 7 Stämmer inte alls

5. Jag känner mig tröttare *medan* jag motionerar.
   Stämmer helt 1 2 3 4 5 6 7 Stämmer inte alls

6. Jag blir tröttare *efter* att jag har motionerat.
   Stämmer helt 1 2 3 4 5 6 7 Stämmer inte alls

7. Jag känner ett ökat välbefinnande *medan* jag motionerar.
   Stämmer helt 1 2 3 4 5 6 7 Stämmer inte alls

8. Jag känner ett ökat välbefinnande *efter* att jag har motionerat.
   Stämmer helt 1 2 3 4 5 6 7 Stämmer inte alls

9. Jag känner mig mer tillfreds med mig själv *medan* jag motionerar.
   Stämmer helt 1 2 3 4 5 6 7 Stämmer inte alls

10. Jag känner mig mer tillfreds med mig själv *efter* att jag har motionerat.
    Stämmer helt 1 2 3 4 5 6 7 Stämmer inte alls

EPA2/2

11. Jag känner mig behagligt avspänd *medan* jag motionerar.
Stämmer helt 1 2 3 4 5 6 7 Stämmer inte alls

12. Jag känner mig behagligt avspänd *efter* att jag har motionerat.
Stämmer helt 1 2 3 4 5 6 7 Stämmer inte alls

13. Smärtan lindras något *medan* jag motionerar.
Stämmer helt 1 2 3 4 5 6 7 Stämmer inte alls

14. Smärtan minskar något *efter* att jag har motionerat.
Stämmer helt 1 2 3 4 5 6 7 Stämmer inte alls

15. Det är viktigt för min hälsa att jag rör på mig och motionerar.
Stämmer helt 1 2 3 4 5 6 7 Stämmer inte alls

16. Jag känner att det är möjligt att anpassa min motion till dagsformen/ hur besvärligt jag har för dagen.
Stämmer helt 1 2 3 4 5 6 7 Stämmer inte alls

17. Jag blir arg för att min kropp inte klarar den motion jag vill utföra.
Stämmer helt 1 2 3 4 5 6 7 Stämmer inte alls

18. Jag tror att jag orkar mer tack vare min motion.
Stämmer helt 1 2 3 4 5 6 7 Stämmer inte alls

19. Jag tror att jag vore i ett sämre fysiskt tillstånd om jag inte motionerade.
Stämmer helt 1 2 3 4 5 6 7 Stämmer inte alls

20. Jag tror att jag klarar svårigheter och motgångar bättre för att jag motionerar.
Stämmer helt 1 2 3 4 5 6 7 Stämmer inte alls

21. Jag känner mig motiverad att motionera även när jag har ont.
Stämmer helt 1 2 3 4 5 6 7 Stämmer inte alls

22. Jag är nöjd med antalet tillfällen i veckan jag motionerar.
Stämmer helt 1 2 3 4 5 6 7 Stämmer inte alls

23. Vilken typ/ vilka typer av motion tänkte du på när du besvarade frågorna? Skriv nedan!

____

02·1094

Namn ______________________________ Datum ____________________

Fysisk aktivitet på arbetsplatsen och i hemmet

Vi vill veta hur mycket Du går och lyfter under en vecka. Ringa in det alternativ som bäst motsvarar dina arbetsuppgifter under den senaste månaden, och ange antalet timmar du uppskattningsvis hur ägnat dig åt dessa aktiviteter.

A. förvärvsarbete

1. Mestadels stillasittande arbete.
Övervägande stillasittande arbete, tex. datorarbete. _____ timmar i veckan

2. *Lättare men rörligt arbete.*
Du har ett lättare arbete, där du går eller står ganska mycket, men ej bär eller lyfter saker som väger över 3 kg. Tex. sköterska, affär, lättare industriarbete. _____ timmar i veckan

3. *Något ansträngande fysiskt arbete.*
Du har något tyngre förvärvsarbe med gång, trappgång eller tyngre lyft (över 3 kg). Tex. vård- eller servicearbete, brevbäring. _____ timmar i veckan

4. *Tungt fysiskt arbete.*
Tex. tungt kroppsarbete med tunga lyft, tex i samband med tungt vård- lager- eller jordbruksarbete. _____ timmar i veckan

B. Arbete i hemmet

1. *Lättare hushållsarbete* som innebär stående och gående, tex. matlagning, strykning, damning, undanplockning. _____ timmar i veckan

2. *Något ansträngande hushållsarbete*, såsom damsugning, bäddning, veckostädning, bakning eller matlagning, lyft av objekt som väger över 3 kg, tex. vård av småbarn. _____ timmar i veckan

3. *Tyngre* reparationer eller vård av anhörig hemma. _____ timmar i veckan

Summa ____________________timmar i veckan i hemmet och i arbete.

L1MAE

Namn ______________________________ Datum ______________

Fysisk aktivitet eller motion på fritiden

Med hjälp av detta frågeformulär vill vi veta hur aktiv du <u>i genomsnitt</u> har varit på fritiden <u>under den senaste veckan.</u> Till fritid inberäknas också promenader till arbete, affär, osv. Du kan ringa in flera svarsalternativ om du har ägnat dig både åt lätta och ansträngande aktiviteter. Vi räknar ihop den sammanlagda tiden.

Stillasittande fritidsaktiviteter.

1. Mestadels stillasittande.

Lätt aktivitet som inte höjer andningsfrekvensen.
Ex. Långsam promenad eller cykling, lätt bassängträning eller lugn simning, lätt trädgårdsarbete.

1. Lätt motion, sammanlagt 0,5 – 1,5 timmar i veckan
2. Lätt motion 2-4 timmar i veckan.
3. Lätt motion mer än 4 timmar i veckan. Ange antalet timmar ________

Måttigt ansträngande motion som påverkar andningen något (andas snabbare) och ev. även kroppstemperaturen (svett), men Du kan föra samtal medan Du motionerar.
Ex. Rask promenad el. cyckling, promenad på naturstigar, lätt jogging, måttligt ansträngande trädgårdsarbete, bassängträning, gymnastik osv.

1. Måttligt ansträngande motion 0,5 – 1,5 timmar i veckan.
2. Måttligt ansträngande motion 2-4 timmar i veckan.
3. Måttligt ansträngande motion mer än 4 timmar i veckan.

Ange antalet timmar ________

Ansträngande motion eller träning som märkbart påverkar andningen och kroppstemperaturen.
Ex. Snabb gång, jogging, annan konditions- eller styrketräning, bollspel, tungt trädgårdsarbete, osv.

1. Ansträngande motion 0,5 – 1,5 timmar i veckan.
2. Ansträngande motion 2-4 timmar i veckan.
3. Ansträngande motion mer än 4 timmar i veckan. Ange antalet timmar ________

Summa ____________________ timmar i veckan

Vilka aktiviteter tänkte du när du fyllde i formuläret: ______________________________

PSQI

PSQI

Namn: ....

Följande frågor avser dina vanliga sömnvanor, men endast under den senaste månaden. Ange det svar som stämmer bäst för flertalet av dagar och nätter under senaste månaden. Var snäll och besvara alla frågor.

1. Under den senaste månaden, när har du vanligtvis gått till sängs på kvällen?
VANLIGTVIS GÅTT TILL SÄNGS KLOCKAN:

2. Under den senaste månaden, hur lång tid har det vanligtvis tagit innan du somnat?
ANTAL MINUTER:

3. Under den senaste månaden, när har du vanligtvis stigit upp på morgonen?
VANLIGTVIS STIGIT UPP KLOCKAN:

4. Under den senaste månaden, hur många timmars faktisk sömn har du fått per natt? (Detta kan skilja sig från antalet timmar du tillbringat i sängen.)
SÖMN (TIMMAR) PER NATT:

Markera det svar som stämmer bäst in på dig för var och en av de återstående frågorna. Var snäll och besvara samtliga frågor.

5. Under den senaste månaden, hur ofta har du haft problem med sömnen för att du:	INTE UNDER DEN SENASTE MÅNADEN	MINDRE ÄN EN GÅNG I VECKAN	EN ELLER TVÅ GÅNGER I VECKAN	MINST TRE GÅNGER I VECKAN
(a) inte kan somna inom 30 minuter	☐	☐	☐	☐
(b) vaknar mitt i natten eller tidigt på morgonen	☐	☐	☐	☐
(c) behöver stiga upp för att gå på toaletten	☐	☐	☐	☐
(d) har svårt att andas	☐	☐	☐	☐
(e) har hosta eller snarkar högt	☐	☐	☐	☐
(f) är frusen	☐	☐	☐	☐
(g) tycker det är för varmt	☐	☐	☐	☐
(h) har obehagliga drömmar	☐	☐	☐	☐
(i) har värk	☐	☐	☐	☐
(j) har annan orsak	☐	☐	☐	☐

	MYCKET GOD	GANSKA GOD	GANSKA DÅLIG	MYCKET DÅLIG
6. Under den senaste månaden, hur skulle du allmänt bedöma din sömnkvalitet?	☐	☐	☐	☐

	INTE UNDER DEN SENASTE MÅNADEN	MINDRE ÄN EN GÅNG I VECKAN	EN ELLER TVÅ GÅNGER I VECKAN	MINST TRE GÅNGER I VECKAN
7. Under den senaste månaden, hur ofta har du tagit medicin (på recept eller receptfritt) för att få hjälp med sömnen?	☐	☐	☐	☐
8. Under den senaste månaden, hur ofta har du haft svårt att hålla dig vaken när du kört bil, suttit och ätit eller deltagit i sociala aktiviteter?	☐	☐	☐	☐

	INGET PROBLEM ALLS	BARA MYCKET LITET PROBLEM	EN DEL PROBLEM	ETT MYCKET STORT PROBLEM
9. Under den senaste månaden, hur stort problem har du haft med att bibehålla tillräcklig entusiasm för att få saker gjorda?	☐	☐	☐	☐

MFI 1/2

Genom följande påståenden vill vi få en uppfattning om **hur Du känt Dig de senaste dagarna.** Som ett exempel, tänk på påståendet "jag har känt mig avspänd". Om Du tycker att det stämmer fullständigt med hur Du har känt Dig, sätt då ett kryss längst till vänster, så här:

ja, det stämmer ☐ ☐ ☐ ☐ ☐ nej, det stämmer inte

Ju mindre Du instämmer med påståendet, desto längre till höger placerar Du krysset. Var vänlig och ta ställning till samtliga påståenden.

1. Jag känner mig i form	ja, det stämmer ☐ ☐ ☐ ☐ ☐ nej, det stämmer inte
2. Kroppsligt känner jag mig bara i stånd att göra väldigt lite	ja, det stämmer ☐ ☐ ☐ ☐ ☐ nej, det stämmer inte
3. Jag känner mig mycket aktiv	ja, det stämmer ☐ ☐ ☐ ☐ ☐ nej, det stämmer inte
4. Jag har lust att göra en massa trevliga saker	ja, det stämmer ☐ ☐ ☐ ☐ ☐ nej, det stämmer inte
5. Jag känner mig trött	ja, det stämmer ☐ ☐ ☐ ☐ ☐ nej, det stämmer inte
6. Jag tycker att jag hinner med mycket på en dag	ja, det stämmer ☐ ☐ ☐ ☐ ☐ nej, det stämmer inte
7. När jag gör något kan jag koncentrera mig på det	ja, det stämmer ☐ ☐ ☐ ☐ ☐ nej, det stämmer inte
8. Kroppsligt orkar jag mycket	ja, det stämmer ☐ ☐ ☐ ☐ ☐ nej, det stämmer inte
9. Jag fasar för att behöva göra något	ja, det stämmer ☐ ☐ ☐ ☐ ☐ nej, det stämmer inte

MFI 2/2

10. Jag får väldigt lite gjort under en dag — ja, det stämmer ☐☐☐☐☐ nej, det stämmer inte

11. Jag har lätt för att koncentrera mig — ja, det stämmer ☐☐☐☐☐ nej, det stämmer inte

12. Jag är utvilad — ja, det stämmer ☐☐☐☐☐ nej, det stämmer inte

13. Jag använder mycket kraft för att koncentrera mig på saker — ja, det stämmer ☐☐☐☐☐ nej, det stämmer inte

14. Kroppsligt känner jag mig i dålig form — ja, det stämmer ☐☐☐☐☐ nej, det stämmer inte

15. Jag har massor av planer — ja, det stämmer ☐☐☐☐☐ nej, det stämmer inte

16. Jag blir lätt trött — ja, det stämmer ☐☐☐☐☐ nej, det stämmer inte

17. Jag får inte mycket gjort — ja, det stämmer ☐☐☐☐☐ nej, det stämmer inte

18. Jag har ingen lust att göra något — ja, det stämmer ☐☐☐☐☐ nej, det stämmer inte

19. Mina tankar far lätt iväg — ja, det stämmer ☐☐☐☐☐ nej, det stämmer inte

20. Kroppsligt känner jag mig i utmärkt form — ja, det stämmer ☐☐☐☐☐ nej, det stämmer inte

Fibromyalgi frågeformulär.

FIQ

Anvisning: **För fråga 1 till 10, kryssa i den ruta som stämmer bäst med hur Du hade det under den *senaste veckan*. Om Du vanligtvis inte sysslar med det som frågas efter så stryk eller korsa över den frågan.**

**Klarade Du under senaste veckan i den omfattning Du önskade att:**	**Hela tiden** 0	**Oftast** 1	**Ibland** 2	**Inte alls** 3	
1. Handla?	☐	☐	☐	☐	....
2. Tvätta i maskin?	☐	☐	☐	☐	....
3. Laga mat?	☐	☐	☐	☐	....
4. Diska porslin och kastruller för hand?	☐	☐	☐	☐	....
5. Dammsuga en tjock matta?	☐	☐	☐	☐	....
6. Bädda sängar?	☐	☐	☐	☐	....
7. Gå mer än en kilometer?	☐	☐	☐	☐	....
8. Besöka vänner eller släktingar?	☐	☐	☐	☐	....
9. Arbeta i trädgården?	☐	☐	☐	☐	....
10. Köra bil?	☐	☐	☐	☐	....

Anvisning: Ringa in en siffra i vardera två följande frågor.

.. Summa

....

11. Hur många dagar mådde Du bra av de senaste 7 dagarna?

**0**	**1**	**2**	**3**	**4**	**5**	**6**	**7**
100	86	71	57	43	29	14	0

Må bra

....

12. Hur mycket var Du sjukledig under den gångna veckan på grund av Din fibromyalgi?
Hoppa över frågan om Du är hemarbetande eller "ålderspensionär"

0% 25% 50% 75% 100% (Ringa in!)

Sjukskr

....

Hade Du: Sjukskrivning / sjukbidrag / sjukpension? (Ringa in!)
Om Du var sjukskriven enstaka dagar, i hur många? **1 2 3 4 5** (Ringa in)

Fortsätt på nästa sida

Anvisning: **Markera i de kvarvarande frågorna ett kryss (✗) vid den punkt på linjen som bäst passar in på hur Du mådde *vanligtvis* under den gångna veckan.**

13. Hur mycket påverkade Din värk eller andra symptom på Din fibromyalgi Ditt arbete?

Inga problem med arbete |——————————————| *Stora svårigheter med arbete*

Arbet ....

14. Hur svår har Din värk varit?

Ingen värk |——————————————| *Mycket svår värk*

Värk ....

15. Har Du varit trött under dagarna?

Ingen trötthet |——————————————| *Mycket trött*

Trött ....

16. Hur har Du känt dig då du stiger upp på morgonen?

Vaknar pigg och utvilad |——————————————| *Vaknar mycket trött*

Sömn ....

17. Hur svår har Din stelhet varit?

Ingen stelhet |——————————————| *Mycket stel*

Stel ....

18. Har Du känt Dig spänd, nervös eller orolig under den gångna veckan?

Inte spänd |——————————————| *Mycket spänd*

Spänd ....

19. Har Du känt Dig deppig och nere under den gångna veckan?

Inte nedstämd |——————————————| *Mycket nedstämd*

Dep ....

SF-36 1/5

HÄLSOENKÄT

INSTRUKTION: Detta formulär innehåller frågor om hur Du ser på Din hälsa. Informationen skall hjälpa till att följa hur Du mår och fungerar i Ditt dagliga liv.

Besvara frågorna genom att sätta en ring runt den siffra Du tycker stämmer bäst in på Dig. Om Du är osäker, ringa ändå in den siffra som känns riktigast.

1. I allmänhet, skulle Du vilja säga att Din hälsa är:

(Sätt en ring runt en siffra)

Utmärkt 1

Mycket god 2

God 3

Någorlunda 4

Dålig 5

2. Jämfört med för ett år sedan, hur skulle Du vilja bedöma Ditt allmänna hälsotillstånd nu?

(Sätt en ring runt en siffra)

Mycket bättre nu än för ett år sedan 1

Något bättre nu än för ett år sedan 2

Ungefär detsamma 3

Något sämre nu än för ett år sedan 4

Mycket sämre nu än för ett år sedan 5

Copyright © 1994 Medical Outcomes Trust.
All rights reserved.
(IQOLA SF-36 Standard Swedish Version 1.0)

3. De följande frågorna handlar om aktiviteter som Du kan tänkas utföra under en vanlig dag. Är Du på grund av Ditt hälsotillstånd begränsad i dessa aktiviteter nu? Om så är fallet, hur mycket?

(Sätt en ring runt en siffra på varje rad)

	Ja, mycket begränsad	Ja, lite begränsad	Nej, inte alls begränsad
a. **Ansträngande aktiviteter**, som att springa, lyfta tunga saker, delta i ansträngande sporter	1	2	3
b. **Måttligt ansträngande aktiviteter**, som att flytta ett bord, dammsuga, skogspromenader eller trädgårdsarbete	1	2	3
c. Lyfta eller bära matkassar	1	2	3
d. Gå uppför **flera** trappor	1	2	3
e. Gå uppför **en** trappa	1	2	3
f. Böja Dig eller gå ner på knä	1	2	3
g. Gå **mer än två kilometer**	1	2	3
h. Gå **några hundra meter**	1	2	3
i. Gå **hundra meter**	1	2	3
j. Bada eller klä på Dig	1	2	3

4. Under de senaste fyra veckorna, har Du haft något av följande problem i Ditt arbete eller med andra regelbundna dagliga aktiviteter som en följd av Ditt kroppsliga hälsotillstånd?

(Sätta en ring runt en siffra på varje rad)

	JA	NEJ
a. Skurit ned den **tid** Du normalt ägnat åt arbete eller andra aktiviteter	1	2
b. **Uträttat mindre** än du skulle önskat	1	2
c. Varit hindrad att utföra **vissa** arbetsuppgifter eller andra aktiviteter	1	2
d. Haft **svårigheter** att utföra Ditt arbete eller andra aktiviteter (t.ex. genom att det krävde extra ansträngning)	1	2

Copyright © 1994 Medical Outcomes Trust.
All rights reserved.
(IQOLA SF-36 Standard Swedish Version 1.0)

SF-36 3/5

5. Under de senaste fyra veckorna, har Du haft något av följande problem i Ditt arbete eller med andra regelbundna dagliga aktiviteter som en följd av känslomässiga problem (som t.ex. nedstämdhet eller ängslan)?

(Sätta en ring runt en siffra på varje rad)

	JA	NEJ
a. Skurit ned den **tid** Du normalt ägnat åt arbete eller andra aktiviteter	1	2
b. **Uträttat mindre** än Du skulle önskat	1	2
c. Inte utfört arbete eller andra aktiviteter så **noggrant** som vanligt	1	2

6. Under de senaste fyra veckorna, i vilken utsträckning har Ditt kroppsliga hälsotillstånd eller Dina känslomässiga problem stört Ditt vanliga umgänge med anhöriga, vänner, grannar eller andra?

(Sätt en ring runt en siffra)

Inte alls 1
Lite 2
Måttligt 3
Mycket 4
Väldigt mycket 5

7. Hur mycket värk eller smärta har Du haft under de senaste fyra veckorna?

(Sätt en ring runt en siffra)

Ingen 1
Mycket lätt 2
Lätt 3
Måttlig 4
Svår 5
Mycket svår 6

Copyright © 1994 Medical Outcomes Trust.
All rights reserved.
(IQOLA SF-36 Standard Swedish Version 1.0)

8. Under de senaste fyra veckorna, hur mycket har värken eller smärtan stört Ditt normala arbete (innefattar både arbete utanför hemmet och hushållssysslor)?

(Sätt en ring runt en siffra)

Inte alls 1

Lite 2

Måttligt 3

Mycket 4

Väldigt mycket 5

9. Frågorna här handlar om hur Du känner Dig och hur Du haft det under de senaste fyra veckorna. Ange för varje fråga det svarsalternativ som bäst beskriver hur Du känt Dig. Hur stor del av tiden under de senaste fyra veckorna ...

(Sätt en ring runt en siffra på varje rad)

	**Hela Tiden**	**Största delen av tiden**	**En hel del av tiden**	**En del av tiden**	**Lite av tiden**	**Inget av tiden**
a. Har Du känt Dig riktigt pigg och stark?	1	2	3	4	5	6
b. Har Du känt Dig mycket nervös?	1	2	3	4	5	6
c. Har Du känt Dig så nedstämd att ingenting kunnat muntra upp Dig?	1	2	3	4	5	6
d. Har Du känt Dig lugn och harmonisk?	1	2	3	4	5	6
e. Har Du varit full av energi?	1	2	3	4	5	6
f. Har Du känt Dig dyster och ledsen?	1	2	3	4	5	6
g. Har Du känt Dig utsliten?	1	2	3	4	5	6
h. Har Du känt Dig glad och lycklig?	1	2	3	4	5	6
i. Har Du känt Dig trött?	1	2	3	4	5	6

Copyright © 1994 Medical Outcomes Trust.
All rights reserved.
(IQOLA SF-36 Standard Swedish Version 1.0)

JF. 365/5

10. Under de senaste fyra veckorna, hur stor del av tiden har Ditt kroppsliga hälsotillstånd eller Dina känslomässiga problem stört Dina möjligheter att umgås (t ex hälsa på släkt, vänner, etc.)?

(Sätt en ring runt en siffra)

Hela tiden 1

Största delen av tiden 2

En del av tiden 3

Lite av tiden 4

Inget av tiden 5

11. Välj det svarsalternativ som bäst beskriver hur mycket vart och ett av följande påståenden STÄMMER eller INTE STÄMMER in på Dig.

(Sätt en ring runt en siffra på varje rad)

	**Stämmer precis**	**Stämmer ganska bra**	**Osäker**	**Stämmer inte särskilt bra**	**Stämmer inte alls**
a. Jag verkar ha lite lättare att bli sjuk än andra människor	1	2	3	4	5
b. Jag är lika frisk som vem som helst av dem jag känner	1	2	3	4	5
c. Jag tror min hälsa kommer att bli sämre	1	2	3	4	5
d. Min hälsa är utmärkt	1	2	3	4	5

Copyright © 1994 Medical Outcomes Trust.
All rights reserved.
(IQOLA SF-36 Standard Swedish Version 1.0)

PGIC

PATIENT GLOBAL IMPRESSION OF CHANGE
(PGIC)

Sedan studiens början har min Fibromyalgi totalt sett:

1 Förbättrats väldigt mycket

2 Förbättrats mycket

3 Förbättrats lite grann

4 Ingen förändring

5 Försvårats lite grann

6 Försvårats mycket

7 Försvårats väldigt mycket

Translation by: Karin Jensen, January 2005.
Contact: Karin.B.Jensen@cns.ki.se

Att: Eva Kosek
Institutionen för Klinisk Neurovetenskap
Karolinska Institutet
Retzius väg 8
171 77 Stockholm

Intyg om ekonomiska och personella resurser

Härmed intygar jag att *ST-läkare Jan Bjersing* har ekonomiska och personella resurser att delta i projektet "Effekten av fysisk träning på sjukdomsmekanismer och symtom vid fibromyalgi" och kan använda sin arbetsplats för detta.

Göteborg 2010-06-17

Boel Mörck
Verksamhetschef
Reumatologen SU

Angående etikansökan:

"Effekten av fysisk träning på sjukdomsmekanismer och symtom vid fibromyalgi".

Härmed intygas att Rehabiliteringsmedicinska Universitetskliniken, Danderyds Sjukhus AB, Stockholm ansvarar för forskningspersonernas säkerhet samt att erforderliga resurser finns tillgängliga för att genomföra känselundersökningar, provtagning och träningsinterventionerna för försökspersonerna i Stockholm inom ramen för denna studie.

Stockholm den / ~~2009~~
23/6 2010

Verksamhetschef

Mikael Ryd

Rehabilitetingsmedicinska Universitetskliniken,

Danderyds Sjukhus AB, 182 88 Stockholm.

Angående etikansökan:

"Effekten av fysisk träning på sjukdomsmekanismer och symtom vid fibromyalgi".

Härmed intygas att Institutionen för Klinisk Neurovetenskap, Karolinska Institutet, Stockholm ansvarar för forskningspersonernas säkerhet samt att erforderliga ekonomiska och personella resurser finns tillgängliga för att genomföra känselundersökningar och funktionell magnetisk resonans imaging (fMRI) för försökspersonerna i Stockholm inom ramen för denna studie.

Stockholm den 16/6 2010

Prefekt Prof. Överläkare Jan Hillert

Institutionen för Klinisk Neurovetenskap

Karolinska Institutet

171 77 Stockholm

Bil 9

VÄSTRA GÖTALANDSREGIONEN
PRIMÄRVÅRDEN SÖDRA ÄLVSBORG

NärRehab Mittenälvsborg

Angående etikansökan:

"Effekten av fysisk träning på sjukdomsmekanismer och symtom vid fibromyalgi".

Härmed intygar jag att leg sjukgymnast Anette Larsson och hennes medarbetare i projektet har ekonomiska resurser att delta i studien "Effekten av fysisk träning på sjukdomsmekanismer och symtom vid fibromyalgi", och kan använda sin arbetsplats för undersökningar och träningsinterventioner inom ramen för denna studie.

Alingsås 2010-07-01

Helen Andersson

Verksamhetschef för När Rehab Alingsås

Alingsås lasarett

Tel 0322-226285

Helen.andersson@vgregion.se

Angående etikansökan:

"Effekten av fysisk träning på sjukdomsmekanismer och symtom vid fibromyalgi".

Härmed intygas att Smärt- och rehabiliteringscentrum, Universitetssjukhuset, Linköping ansvarar för forskningspersonernas säkerhet samt att erforderliga ekonomiska och personella resurser finns tillgängliga för att genomföra känselundersökningar, provtagning och träningsinterventionerna för försökspersonerna i Linköping inom ramen för denna studie.

Linköping 2010-06-10

Björn Gerdle

Verksamhetschef, professor

Smärt- och rehabiliteringscentrum

US, 581 85 Linköping

Angående etikansökan:

”Effekten av fysisk träning på sjukdomsmekanismer och symtom vid fibromyalgi”.

Härmed intygar jag att docent Kaisa Mannerkorpi och hennes medarbetare i projektet har ekonomiska resurser att delta i studien ”Effekten av fysisk träning på sjukdomsmekanismer och symtom vid fibromyalgi”, och kan använda sin arbetsplats för undersökningar och träningsinterventioner inom ramen för denna studie.

DATUM 2010-06-09

Verksamhetschef

Department of Dental Medicine
Kaj Fried
Professor, Chairman

Angående etikansökan:
"Effekten av fysisk träning på sjukdomsmekanismer och symtom vid fibromyalgi".
Härmed intygas att enheten för klinisk oral fysiologi, Institutionen för odontologi, Karolinska Institutet, Huddinge ansvarar för forskningspersonernas säkerhet samt att erforderliga ekonomiska och personella resurser finns tillgängliga för att genomföra mikrodialysundersökning och hudbiopsitagning för försökspersonerna i Stockholmsområdet inom ramen för denna studie.

Kaj Fried
Professor, Prefekt

Postal address
PO Box 4064
SE-141 04 HUDDINGE, Sweden
Org. number 202100 2973

Visiting address
Alfred Nobels Allé 8
HUDDINGE

Telephone
+46 8 524 878 53 or
+46 8 524 878 60

Fax
+46 8 711 83 43

Kaj.Fried@ki.se

B:1 10

Meritförteckning

CURRICULUM VITAE

Eva Kosek, MD, Associate Professor
Born October 26, 1960
Associate Professor, Department of Clinical Neuroscience, Karolinska Institute, SE-171 76 Stockholm, Sweden
Phone: +46 (0) 517 76299, Fax: +46 (0) 8 51773266, E-Mail: Eva.Kosek@ki.se

1. University exam
1986 MD, Uppsala University

2. Doctoral degree
1996 PhD in Medical rehabilitation, Karolinska Institute, Stockholm (Advisor: Prof Per Hansson and Prof. Jan Ekholm)

3. Postdoctoral appointment
N/A

4. Associate professor (Docent)
2004 Clinical Pain Research, Karolinska Institute, Stockholm

5. Present employment
Associate Professor (klinisk lektor), Department of Clinical Neuroscience, Karolinska Institute and senior consultant (överläkare) Department of Rheumatology, Karolinska University Hospital. Currently 50% research and 50% work as sensior consultant (at Stockholm Spine Center (currently on leave from Dept. of Rheumatology).

6. Employment history
1987-89 Resident, Gävle Hospital.
1989 Attained Swedish doctors licence
1989-93 Research assistant, Department of Rehabilitation Medicine, Karolinska Institute/Hospital, Stockholm.
1992-96 PhD-student, Department of Rehabilitaiton Medicine, Karolinska Institute.
1996-2004 Postdoctoral research activity at Karolinska Institute
1993- Doctor at Department of Rehabilitation Medicine, Karolinska Hospital.
1998 Specialist in Rehabilitation Medicine.
1998-2001 Specialist doctor at the Department of Rehabilitation Medicine, Karolinska Hospital.
2001 Specialist in pain relief.
2001-2004 Senior consultant (överläkare) at the Department of Rehabilitation Medicine, Karolinska Hospital
2004- Assistant professor, Karolinska Institute
2005-2006 Senior consultant at the Pain Center, Department of Neurosurgery, Karolinska University Hospital
2007-2009 Senior consultant at Stockholm Spine Center, Löwenströmska Hospital, Upplands Väsby.

7. Deductible time
1989-1991 Parental leave with children born 1989 and 1990

8. Doctoral supervisor
Physical therapist Ann-Sofie Leffler, who attained her Ph.D. 2002-02-01, Karolinska Institute.
Psychologist Karin Jensen, who attained her PhD 2009-10-23, Karolinska Institute.
Currently:
Supervisor for MD Diana Kadetoff, registered 2007-11-27 at the Karolinska Institute.
Supervisor for MD Fredrik Lindstedt, to be registered at Karolinska Institute.

9. Awards and Honours
1996 Astras award for pain research 25 000kr
2007 Nanna Schwartz award for good science within the field of rheumatology

Invited speaker at international congresses (2005-):
Invited speaker at American Pain Society 4th Annual Scientific Meeting , Boston, April 2005.
European League Against Rheumatism (EULAR) yearly congress satellite symposium, Barcelona, Spain 2007.
4th Congress of World Institute of Pain (WIP) congress satellite symposium, Budapest, Hungary 2007.
Invited speaker at Latin American Pain Forum, Cancun, Mexiko, April 2008
Invited speaker at 76th Annual Meeting of the Swiss Society of Internal Medicine (SSIM), Lausanne, Switzerland, Maj 2008.
Invited speaker at a satellite symposium at the European College of Neuropsychopharmacology (ECNP), Barcelona, Spain, September 2008
European League Against Rheumatism (EULAR) yearly congress satellite symposium, Copenhagen, Denmark 2009.
Invited speaker at European League Against Rheumatism (EULAR) yearly congress, Rome, Italy, June 2010.
Invited to speaker and organizer/chair of topical workshop on fibromyalgia, 13th World Congress on Pain (International Association for the Study of Pain, IASP), Montreal, Canada, August 2010.

10. Important national and international assignments
Reviewer for several scientific journals. Member of professional societies such as the International Association for the Study of Pain and the Scandinavian Association for the Study of Pain. Member of the Swedish Medical Association, board member in the Swedish Medical Association for Pain Relief (2003-2007). Board member of the Karolinska Institute Center for Pain research since 2005. Dr Kosek was a member of the European League Against Rheumatisms task force providing European guidelines for the treatment of fibromyalgia (Carville et al. 2007) and is working with further development of new classification criteria. Furthermore, Dr Kosek is also a member in an international research network regarding pain research in fibromyalgia, osteoarthritis and rheumatoid arthritis (chaired by Prof. Kidd, London and Prof Dieppe, Bristol).
Dr. Kosek has participated in advisory board meetings for several pharmaceutical companies in the pain area i.e., Pfizer, Lilly, Whyett, Sanofi-Avertis and Pierre Fabre as an expert on chronic pain and fibromyalgia.

11. Research collaborations
Dr. Kosek has established research collaboration with the pharmaceutical company Pierre Fabre Medicaments regarding the effects of serotonin-noradrenalin re-uptake inhibitors on fibromyalgia symptoms. Studies were performed using quantitative sensory testing and assessment of pain related brain activity (functional magnetic imaging, fMRI). These studies are performed in collaboration with Kings College, UK, Ann Harbor University, Michigan, and University of Cologne, Germany.
Dr. Koseks research group has ongoing research collaboration with other research groups namely:
Department of Rheumatology, Karolinska Institute, regarding the importance of inflammation for symptoms in fibromyalgia patients (i.e, assessment of cytokines in blood and cerebrospinal fluid in relation to autonomic response (heart rate variability) and symptoms.
Department of Physiology and Pharmacology, Karolinska Institute, regarding translational studies of inflammation, pain sensitivity and chronic musculoskeletal pain.
Department of Orthopedic Surgery, University of Lund, regarding the effect of physical exercise and surgery on endogenous pain modulation in patients with knee and hip osteoarthritis.
Department of Radiology, Karolinska University Hospital, regarding the influence of pain on intramuscular blood flow (ultrasound Doppler).

12. Major grants with Dr. Kosek as main applicant
* Risk factors for pain development in patients with osteoarthritis and low back pain. Karolinska Institute Foundation (Ugglas Found) 2006: 80 000 SEK
* The effect of static muscle work and mental stress on muscle blood flow, autonomic nervous system function and endogenous pain modulatory mechanisms in healthy subjects and patients with muscle or joint pain. Stockholm County Council (ALF) 2006-2008: 1 100 000 SEK

* The importacne of opioidergic pain modulatory mechanisms in chronic musculoskeletal pain; in relation to symptoms and treatment effects. Stockholm County Council (ALF) 2010: 500 000 SEK
* The importance of central nervous system mechanisms in fibromyalgia patients; implications for development of new treatments. Swedish Research Council (Vetenskapsrådet) 2009-2011 (K2009-53X-21070-01-3): 1 050 000 SEK
* Pain research in rheumatic diseases: Swedish Rheumatism Association (Reumatikerförbundet) 2009-2011: 1 450 000 SEK
* Risk factors for treatment failure in patients with hip- or knee osteoarthritis. Karolinska Institute Foundation (Ugglas Found) 2009: 120 000 SEK
* Chronic pain: from desktop to patient. Swedish Strategic Research Foundation (Stiftelsen för Strategisk forskning, RBa08-0062)(Kosek is clinical director) 2010-1014 (with a half time control 2012): 12 000 000 SEK

13. Scientific publications
27 articles in peer-reviewed international journals and 12 book chapters mainly within the fields of clinical pain, pain mechanisms in chronic musculoskeletal pain, fibromyalgia and rehabilitation.

14. References of interest
* Kosek, E., Ekholm, J. and Hansson, P., Increased pressure pain sensibility in fibromyalgia patients is located deep to the skin but not restricted to muscle tissue, Pain, 63 (1995) 335-339.
* Kosek, E., Ekholm, J. and Hansson, P., Modulation of pressure pain thresholds during and following isometric contraction in patients with fibromyalgia and in healthy controls, Pain, 64 (1996a) 415-423.
* Kosek, E., Ekholm, J. and Hansson, P., Sensory dysfunction in fibromyalgia patients with implications for pathogenic mechanisms, Pain, 68 (1996b) 375-383.
* Kosek, E. and Hansson, P., Modulatory influence on somatosensory perception from vibration and heterotopic noxious conditioning stimulation (HNCS) in fibromyalgia patients and healthy subjects, Pain, 70 (1997) 41-51.
* Berglund, B., Harju, E.-L., Kosek, E. and Lindblom, U., Quantitative and qualitative perceptual analysis of cold dysesthesia and hyperalgesia in fibromyalgia, Pain, 96 (2002) 177-187.
* Kosek, E. and Lundberg, L., Segmental and plurisegmental modulation of pressure pain thresholds during static muscle contractions in healthy individuals. Eur. J Pain 7 (2003) 251-258.
* Elvin, A., Siösteen, A-K., Nilsson, A. and Kosek E. Decreased muscle blood flow in fibromyalgia patients during standardized muscle exercise. A contrast media enhanced colour doppler study. Eur J Pain 10 (2006) 137-144.
* Kadetoff. D.and Kosek, E., The effects of static muscle contraction on blood pressure, heart rate and pressure pain thresholds in fibromyalgia patients. Eur J Pain 2007 Jan;11(1):39-47.
* Carville SF, Arendt-Nielsen L, Bliddal H, Blotman F, Branco JC, Buskila D, DaSilva JAP, Danneskiøld-Samsøe B, Dincer F, Henriksson C, Henriksson KG, Kosek E, Longley K, McCarthy GM, Perrot S, Puszczewicz M, Sarzi-Puttini, Silman A, Späth M and Choy EH., EULAR evidence based recommendations for the management of fibromyalgia syndrome. Ann Rheum Dis, 2008;67(4):536-541.
* Jensen KB, Kosek E, Petzke F, Carville S, Fransson P, Marcus H, Williams SCR, Choy E, Giesecke T, Mainguy Y, Gracely R, Ingvar M. Evidence of dysfunctional pain inhibition in Fibromyalgia reflected in rACC during provoked pain. Pain 2009;144:95-100.
* Jensen, KB, Lonsdorf TB, Schalling M, Kosek E, Ingvar M. Increased sensitivity to thermal pain following a single opiate dose is influenced by the COMT val158met polymorphism. PloS One 2009;4:e6016.
* Kosek E, Jenen KB, Lonsdorf TB, Schalling M, Ingvar M. Genetic variation in the serotonin transporter gene (5-HTTLPR, rs25531) influences the analgesic response to the short acting opioid remifentanil in humans. Molecular Pain 2009;5:37.

Bil 11

Bilaga

Ersättning till försökspersoner för studien "Effekten av fysisk träning på sjukdomsmekanismer och symtom vid fibromyalgi".

Friska försökspersoner:

Friska försökspersoner som endast deltar i del 1: 500 kr efter avslutad studie.

Friska försökspersoner som deltar i del 1 och 2: Tillgång till träningslokal (alt. gymkort i 6 månader) samt 3000 kr efter avslutad studie.

Friska försökspersoner som förutom styrketräning även deltar i mikrodialys/hudbiopsi och funktionell magnetisk hjärnavbildning (fMRI) får totalt 3500 kr efter genomgången studie.

FM patienter:

FM patienter som deltar i avspänningsgrupp: Tillgång till lokal för avspänningsträning samt 500 kr efter genomgången studie.

FM patienter som deltar i styrketräningsgrupp: Tillgång till träningslokal (alt. gymkort i 6 månader) samt 500 kr efter genomgången studie.

FM patienter som även deltar i fMRI undersökning (men inte mikrodialys) får totalt 1000kr efter genomgången studie.

FM patienter som förutom styrketräning även deltar i mikrodialys/hudbiopsi får totalt 3000 kr efter genomgången studie.

FM patienter som förutom styrketräning även deltar i mikrodialys/hudbiopsi och funktionell magnetisk hjärnavbildning (fMRI) får totalt 3500 kr efter genomgången studie.

Bil 12

Manual för styrketräning vid fibromyalgi – förkortad version

K Mannerkorpi, A Larsson, Avd för Reumatologi och Inflammationsforskning, Sahlgrenska Akademin, Göteborgs universitet. 2010-06-04.

Programmet omfattar träning av följande muskelgrupper: knäböjare, knästräckare, höftsträckare (benpress) buk, rygg, armbågsböjare, fotledsträckare och handstyrka. FP ska påminnas om att aktivera djup bukmuskulatur under övningen. Programmet avslutas med stretching. De första träningstillfällena beräknas ta 1,5 timme (inlärning, social trygghet), men förkortas sedan till en timme.

Bedömning av belastning: Maximal muskelstyrka (1RM) bedöms för varje individ. Utifrån detta räknas motståndet enligt nedanstående modell.

Progress: Vecka 1-4 40-60% av 1RM. Vecka 5-7: 50-70% av 1RM. Vecka 8-11: 60-70% av 1RM. Vecka 12-15: 60-80% av 1RM.

Repetitioner: 40% =15-20, 50% = 12-15, 60% = 10-12, 70% = 8-10, 80% = 5-8.

1. Uppvärmning på cykel 10 min: 40-75 watt.

2. Knäböjare. FP sitter i maskinen med stöd för rygg och baksida lår. Ryggstödet ska ställas in så att FP känner sittdynan i knävecket. FP drar in benen under sig och släpper sedan långsamt tillbaks igen. Räkna 1-2 sek för att böja och 1-2 sek för att sträcka. Belastningen provas ut individuellt och bestäms tillsammans med FP. antal repetitioner utifrån "modell för belastning". Vila 30-45 sek, upprepa ytterligare ett set.

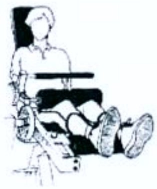

3. Knästräckare. FP sitter i maskinen med rak rygg och stöd för rygg och baksida lår, ställ in ryggstödet så att du känner sittdynan i knävecket. FP sträcker båda benen med god kontroll. Räkna 1-2 sek för att sträcka och 1-2 sek för att böja igen. Böj till ca 90° i knäleden. Belastningen provas ut individuellt bestäms tillsammans med antal repetitioner utifrån "modell för belastning"

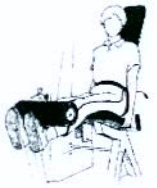

4. Ryggsträckare. FP ligger på mage på golv eller brits. Placera händerna under höfterna. Lyft överkroppen lite från underlaget och dra samtidigt ihop skulderbladen. Fokus på övningen är att aktivera ryggsträckare och skulderbladsmuskulatur. Viktigt att blicken är riktad mot golvet genom hela övningen samt att tårna hålls kvar mot golvet. Upprepa rörelsen så många gånger som FP orkar eller max 20 ggr. Vila 30-45 sek och upprepa sedan ytterligare en gång.

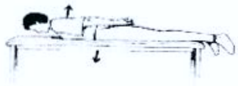

5. Bukmuskler. FP ligger på rygg med armarna utefter sidorna och benen uppdragna i 90° vinkel. Eventuellt behövs en ihoprullad handduk som stöd för ländryggen. FP spänner magen och "doppar" sedan ena hälen i underlaget. Upprepa så många gånger som FP orkar, dock max 20 ggr. Vila sedan 30-45 sek och utför efter det ytterligare ett set med så många som FP orkar (max 20 ggr).

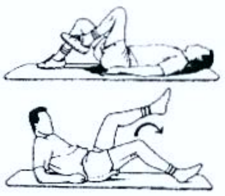

6. Bensträckare. FP sitter i benpressmaskinen, fötterna stadigt placerade på plattan axelbrett isär. Viktigt att FP har stöd för ryggen och att FP håller i handtagen. Tryck ifrån med benen och sträck tills knäna är raka (det är viktigt att inte översträcka knäna med motstånd på). Gå långsamt tillbaks till utgångsläget. Räkna 1-2 sek för att sträcka och 1-2 sek för att böja. Belastningen provas ut individuellt och bestäms tillsammans med antal repetitioner utifrån "Modell för belastning"

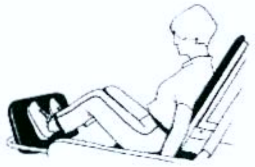

7. Armbågsböjare. FP sitter med rak rygg, sänkta axlar och sträckt nacke. Som motstånd används hantlar. Böj armarna med god kontroll och gå nästan ända upp till full böjning. Sträck sedan ut armen igen. Räkna 1-2 sek för att böja och 1-2 sek för att sträcka ut igen.

Antalet repetitioner bestäms utifrån modell för belastning. Upprepa ytterligare ett set.

8. Tåhävningar. Stå på plant underlag med balansstöd. Gå upp på tå och sänk ner igen. Rörelsen ska vara kontrollerad. Räkna 1-2 sek för att gå upp och 1-2 sek för att gå ner. Upprepa så många gånger FP orkar, max 20 ggr. Vila 30-45 sek. Gör ytterligare ett set.

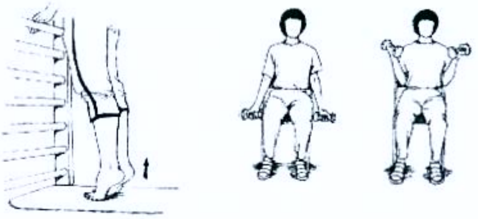

9. Handstyrka. Handstyrkan tränas med hjälp av bollar med olika motstånd. Båda händerna tränas samtidigt. FP kramar bollen så hårt det går och släpper sedan igen. Upprepa så många gånger FP orkar eller max 20 ggr. Vila 30-45 sek. Upprepa ytterligare ett set.

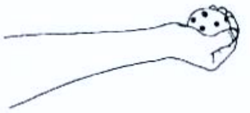

¤¤¤¤

Manual för avslappningsträning vid fibromyalgi – förkortad version
M Löfgren, Rehabiliteringsmedicinska Universitetskliniken Stockholm Danderyds sjukhus, institutionen för kliniska vetenskaper Danderyds sjukhus, avd för Rehabiliteringsmedicin Karolinska Institutet. 2010-06-15

Programmet omfattar cirka 60 minuter; ett liggande avslappningsprogram under 30 minuter, en diskussion under cirka 10 minuter där deltagarnas erfarenheter av att slappna av samlas ihop och ett stretchingprogram under cirka 20 minuter. Muskelgrupperna som stretchas är följande; skulderhöjare, ryggsträckare, höftsträckare, lårmuskulatur samt vadmuskulatur.

Avslappningsprogrammet är ett så kallat autogent avslappningsprogram. Deltagarna guidas av en i avslappning erfaren sjukgymnast. Under programmet går deltagarna igenom hela kroppen, kroppsdel för kroppsdel, med tanken, de uppmanas att koncentrera sig på att slappna av så mycket som möjligt i de enskilda kroppsdelarna för att i slutet av passet känna att hela kroppen är avslappnad och att andningen blir djupare och mer avslappnad.
Exempel på instruktion: "Ta koncentrationen till din högra fot, känn att du slappnar av i foten, hur den vilar tungt mot golvet...... Ta upp koncentrationen till din högra vad, känn hur vadmuskeln slappnar av, hur den blir mer och mer avslappnad."
Under avslappningen ligger deltagarna ner på en mjuk träningsmatta, de har möjlighet att ha kudde och filt, så att de ligger varmt och bekvämt. De kan när som helst byta ställning eller avbryta avslappningen.
Efter avslappningen berättar deltagarna för varandra hur det har gått, sjukgymnasten kan här förklara bakomliggande teorier och förklara kroppsliga, fysiologiska reaktioner. Deltagarna får i uppgift att träna avslappning hemma, även detta följs upp under diskussionen.

Stretchingprogrammet utförs sedan gemensamt, muskelgrupp för muskelgrupp, enligt vedertagna principer. Sjukgymnasten instruerar gruppen och ger individuella instruktioner vid behov.
